# The blood transcriptome of musk deer under heat stress condition reveals the regulatory mechanism of genes to maintain homeostasis metabolism

**DOI:** 10.1186/s12864-025-11577-y

**Published:** 2025-04-24

**Authors:** Xin Shi, Zhuo Cheng, Chengli Zheng, Kaiqing Wang, Jiandong Yang, Hang Jie, Yang Li, Ming Zhang

**Affiliations:** 1 College of Animal Science and Technology, Sichuan Agricultural University, Chengdu, 611130 China; 2 Sichuan Institute of Musk Deer Breeding, Chengdu, 611845 China; 3 Chongqing Institute of Medicinal Plant Cultivation, Yongchuan, Chongqing, 408435 China; 4 Chengdu Yongkang Pharmaceutical Co., Ltd, Wenjiang distrct, Chengdu, 611130 China; 5 Farm Animal Genetic Resources Exploration and Innovation Key Laboratory of Sichuan Province, Sichuan Agricultural University, Yaan, 611130 China

**Keywords:** Heat stress, Cortisol, Corticosterone, Homeostasis metabolism, Musk deer

## Abstract

**Background:**

Heat stress has a significant adverse impact on both livestock and poultry production, posing a considerable challenge to the artificial breeding of forest musk deer. However, there is a lack of studies on the heat stress of forest musk deer, so it is necessary to understand the effects of temperature and humidity index (THI) variation on these animals.

**Results:**

In according to the local climate characteristics, blood samples were collected during four periods (April, June, July and August) for biochemical indicators and transcriptome sequencing. The results showed that blood cortisol and corticosterone concentrations increased significantly in July (THI = 74.89, *P* < 0.05). Moreover, Na^+^ concentration exhibited a negatively correlated with THI (*r* = -0.959, Pr = 0.041). Blood urea nitrogen (BUN) concentration in July (G3) was significantly lower than that in April (G1) and June (G2) (*P* < 0.05). The total antioxidant capacity (T-AOC) in July was significantly decreased (*P* < 0.05), and the creatine kinase (CK) was significantly higher than that in April and August. There was a significant positive correlation between immunoglobulin G (IgG) and THI (*r* = 0.999, Pr = 0.001) attributable to the significant increase of TNF-α in July (*P* < 0.05). The transcriptomic comparison between G1 and G3 revealed the largest number of differentially expressed genes (DEGs) (6410 up-regulated and 472 down-regulated). Among them, *JAK1*, *AP3B1* and *FKBP15* were the most significantly up-regulated immune-related genes in response to heat stress. Trend analysis indicated that the pathways related to immunity and protein metabolism were particularly impacted by heat stress.

**Conclusions:**

Research indicates that heat stress disrupts the normal metabolism of forest musk deer and adversely affects their immune system, which is attributed to the THI exceeding the threshold that forest musk deer can tolerate. The findings of this study provide valuable data support for the scientific breeding of captive forest musk deer and enhance the understanding of the immune dynamics of ruminants under heat stress.

**Supplementary Information:**

The online version contains supplementary material available at 10.1186/s12864-025-11577-y.

## Background

Forest musk deer is a ruminant species native to Southeast Asia. In China, musk secreted by glands in the lower abdomen of male forest musk deer holds great economic value due to its medicinal properties and use as a perfume ingredient. However, this has led to the depletion of musk deer population in the wild, leading to their classification as critically endangered species on the International Union for Conservation of Nature (IUCN) Red List [1] and the Chinese Vertebrate Red List [2]. Therefore, in order to safeguard the conservation of wild forest musk deer and optimize the utilization of musk, China strongly advocates for artificial breeding as an alternative to hunting in the wild, thus promoting the sustainable development of musk deer resources. However, the high mortality rate of captive forest musk deer in summer hinders the increasing of breeding population and restricts the development of forest musk deer farming. Wild forest musk deer mainly inhabit the subtropical monsoon climates of Sichuan, Shaanxi and Hubei provinces, where they typically undertake vertical migrate during summer to avoid the adverse effects of high temperatures [3].

In summary, heat stress triggers emergency and stress responses through the sympathetic-adrenal-medullary axis (SMA) and the hypothalamic-pituitary-adrenal axis (HPA), activating a cascade of hormonal reactions within the endocrine system. Exposure to heat stress activates the HPA axis, leading to a surge in peripheral glucocorticoid levels, exacerbating anxiety and exerting negative effects on the immune system [4, 5]. Elevated plasma glucocorticoid concentrations increase protein hydrolysis and disrupt lipid metabolism [6, 7]. Excessive glucocorticoids can induce the expression of reactive oxygen species (ROS)-related genes, exacerbating oxidative stress and organ damage [4, 8, 9], thereby increasing animal morbidity. Additionally, heat stress mediates a shift in immune responses from cell-mediated to humoral immunity, weakening the immune system and enhancing susceptibility to various pathogens [10]. Numerous studies have previously explored the regulatory mechanisms linking oxidative stress and immune responses [11–13]. A typical manifestation in ruminants is the active reduction of rumination frequency to minimize metabolic heat production, resulting in feed accumulation in the rumen and subsequent subclinical or acute ruminal acidosis [14]. In conclusion, heat stress inflicts multifaceted harm on animals, including organ damage, oxidative stress, endocrine dysregulation, immune suppression, and reproductive disorders, ultimately leading to reduced productivity and increased mortality [15].

Although the closely related species of forest musk deer, such as cattle and sheep, have some adaptations to heat stress during prolonged domestication, heat stress still causes huge losses to cattle and sheep farming [16–19]. This presents an even greater challenge to captive forest musk deer, given their early stage of domestication, and limited thermal tolerance. Forest musk deer lack the ability to undertake vertical migration in artificial environments to mitigate climate change [3], result in lower productivity and higher morbidity compared to cattle and sheep farming. Furthermore, we conducted an analysis of musk deer health status in two farms situated at different altitudes: Dujiangyan (750 m above sea level) and Maerkang (2700 m above sea level) during summer. Our findings revealed that musk deer in the low altitude farm experienced a higher frequency of illness and required more frequent treatment from April to August each year compared to those at the higher altitude farm. Hence, we postulated that heat stress may serve as one of the contributing factors for high mortality in captive forest musk deer. It is necessary to study the regulatory mechanism of coping with elevated temperature-humidity index (THI) in forest musk deer during the early domestication stage, and finally promote the healthy development of captive musk deer population.

## Methods

### Animal

Animals were fed at the Sichuan Musk Deer Research Institute (Chengdu, China). Samples were collected from 11 male forest musk deer (3–5 years old) during the period from April to August 2023. All animals were fed in individual pen with 30m^2^ of outdoor field, and were supplied adequate the diet and water, and the diet consisted of concentrated feed, juicy feed, and dry leaves (Table 1). The attendants change into on-site work clothes when working, and the living area of the forest musk deer is cleaned and disinfected monthly. During the experiment, in order to avoid the influence of artificial stress, only the feeder entered the enclosure in the morning and evening to check, clean and add the diet and water. The caretakers closely observed behavioral changes through direct, close-range monitoring. Collection of samples was performed in accordance with the regulations for the Administration of Affairs Concerning Experimental Animals (Ministry of Science and Technology, China, revised in June 2004) and was approved by the Institutional Animal Care and Use Committee in the College of Animal Science and Technology, Sichuan Agricultural University, Sichuan, China, under permit No: S20151006.

**Table 1:** Composition and dosage of daily diet for adult forest musk deer

Category	Concentrated feed	Juicy feed	Dry leaves
Component	Fish meal: 16–18% Ca: 1.5% P: 1% Other: Dry leaves	Carrots White radishes Lettuce Cabbage	Elm leaves Mulberry leaves Cherry leaves
Feeding quantity /day/g/head	150	500	100

Note The juicy feed needs to be cut into pieces no larger than 1 cm using a vegetable cutter, mixed evenly before fed

### THI recording and blood collection

The experimental period was April (G1), June (G2), July (G3) and August (G4). Enclosure temperature and humidity were recorded daily. Temperature and humidity index (THI) using the following formula:

THI = 0.81×Td+[(0.99×RH) × (Td-14.4)] + 46.3 (Td: Temperature of dry-bulb, ℃; RH: Relative Humidity, %) [20].

Venous blood samples were collected from the hind limbs of musk deer (4mL per administration per month) with an interval of 30 days. 2mL was collected in anticoagulant free collection tubes, left at room temperature for 30 min, centrifuged at 3000 rpm for 15 min, and the aspirated supernatant was stored at -20 ° C. 2mL was collected in EDTA collection tubes, mixed and transferred to 15mL enzyme-free centrifuge tubes, and 3 times the volume of Trizol was mixed and stored in liquid nitrogen.

### Serum biochemical indicators determining

The concentrations of K^+^ (mmol/L), Na^+^ (mmol/L), Cl^−^ (mmol/L), Ca^2+^ (mmol/L), urea nitrogen (mmol/L), triglyceride (mmol/L), Total antioxidant capacity (mM), malondialdehyde (U/mL), glutathione peroxidase (U/mL) and creatine kinase (U/L) in serum of hind limb vein were detected by automatic biochemical analyzer. Concentrations of cortisol, corticosterone, immune proteins (IgA, IgG and IgM), and inflammatory factors (TNF-α) were determined by ELISA. Commercial kits from Nanjing Jiancheng BioEngineering were used for the above assays. The steps were carried out according to the instructions of the kit.

### Total RNA isolation and sequencing

Total RNA was isolated from musk deer blood using Trizol. The NanoDrop ND-1000 (NanoDrop, Wilmington, DE, USA) was used to perform total RNA quality control, and the Bioanalyzer 2100 (Agilent, CA, USA) was used to check RNA integrity. The library construction and sequencing were commissioned by Novogene Co.,Ltd. They used Illumina Novaseq™ 6000 (Novogene Co., Ltd., Beijing, China) for paired-reads sequencing in PE150 mode according to standard operation. All reagents and kits were used in accordance with the manufacturer’s guidelines.

### Data handling procedures

Unqualified sequences in the raw data were filtered using Cutadapt (v1.9, rapid trimming and repairing adapter sequences and low-quality bases in high-throughput sequencing data), and sequence quality was checked using FastQC software (http://www.bioinformatics.babraham.ac.uk/projects/fastqc/, v0.11.9, a tool for rapid assessment of high-throughput sequencing data quality). The Mapped reads were obtained by sequence alignment of clean data with our assembled musk deer genome (unpublished) using Hisat2 (v2.2.1, a tool that supports the alignment of reads for multiple genome types and a large number of parallel processing). StringTie (v2.1.6, a software for transcript assembly and quantification, excelling in accurately identifying and quantifying transcripts, particularly in multi-sample comparisons.) was used to estimate the relative abundance of transcripts with read count values. Data were corrected to relative logarithmic expressions for normalization using the DESeq2 R library. To determine grouping between samples, principal coordinate analysis (PCA) was performed using the princomp function of R (http://www.r-project.org/). All genes were analyzed using the DESeq2 function of R, with fold change (FC) ≥ 2 (|log2FC| ≥ 1) and *p* < 0.05 as the threshold for screening differentially expressed genes. Trend analysis using Mfuzz R library for clustering and visualization.

### Gene ontology and KEGG pathway enrichment analysis

Using the clusterProfiler package in R, GO and KEGG enrichment analyses were performed, and the Benjamini-Hochberg process was applied to adjust the p-values for multiple hypothesis testing to obtain the Q values.

### Statistical analysis

Excel 2010 was used to sort out the original data and eliminate abnormal data. SAS 9.4 was used for one-way analysis of variance. Test data are expressed as Mean ± SD. *p* < 0.05 was considered statistically significant.

## Results

### Changes in blood biochemical indicators of forest musk deer induced by different THI levels

### Variations in blood stress hormones concentrations

**Fig. 1:**
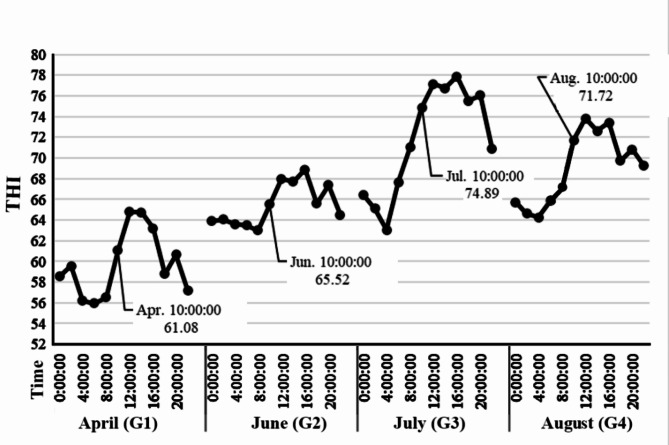
Trends of THI values at different time on the collected-blood day

**Fig. 2:**
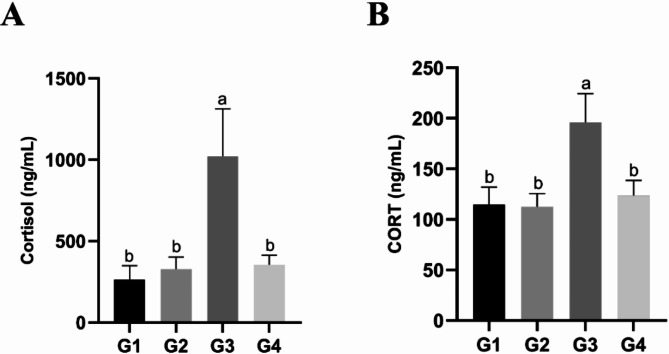
Effect of THI changes on serum cortisol and corticosterone of forest musk deer. (**A**) Cortisol concentration changes at different THI; (**B**) Corticosterone (CORT) concentration changes at different THI. G1: April, G2: June, G3: July, G4: August

We conducted 24-hour monitoring of temperature and humidity within the forest musk deer enclosure (Table S1). Analysis of the mean values of the THI revealed that July had the highest THI (Fig. 1). Blood samples were collected at 10:00 am in April, June, July and August, and serum was separated for biochemical analysis. The results indicated that the cortisol and corticosterone level in G3 (July) significantly increased compared to G1 (April), G2 (June) and G4 (August) (Fig. 2A and B). This indicates that forest musk deer were already experiencing heat stress when the THI was 74.89.

### Variations in blood ion and metabolite concentrations

**Fig. 3:**
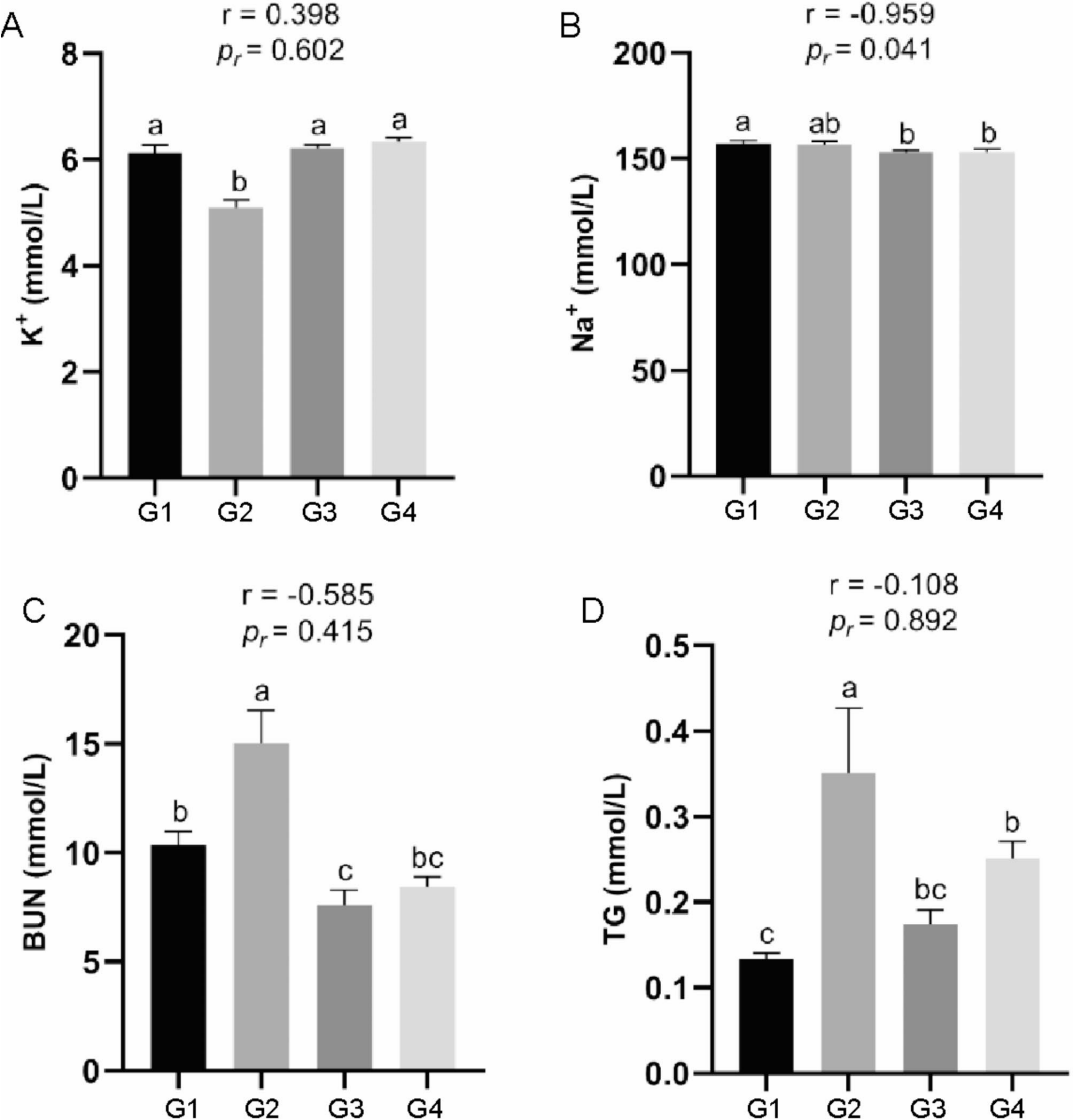
Effects of THI changes on osmotic pressure and metabolic levels of forest musk deer. (**A**) K^+^ concentration of serum; (**B**) Na^+^ concentration of serum; (**C**) BUN concentration of serum; (**D**) TG concentration of serum

Furthermore, we investigated several other indices reflecting the homeostasis of forest musk deer. The potassium ion (K^+^) concentration was significantly lower in G2 compared to the other groups (Fig. 3A). Conversely, the sodium ion (Na^+^) concentration of G1 was significantly higher than that in G3 and G4 (Fig. 3B). Heat stress did not exert any significant effect on chloride ion (Cl^−^) and calcium ion (Ca^2+^) concentrations in the serum (Figure S1A and S1B). G2 had significantly higher blood urea nitrogen (BUN) and triglyceride (TG) levels than the other groups contrast, the BUN levels in G3 and G4 were significantly lower than those in G1 and G2 (Fig. 3C and D). The above results indicate that different THI levels significantly affect the stability of blood biochemical parameters in forest musk deer.

### Variations in blood immune factor concentrations

**Fig. 4:**
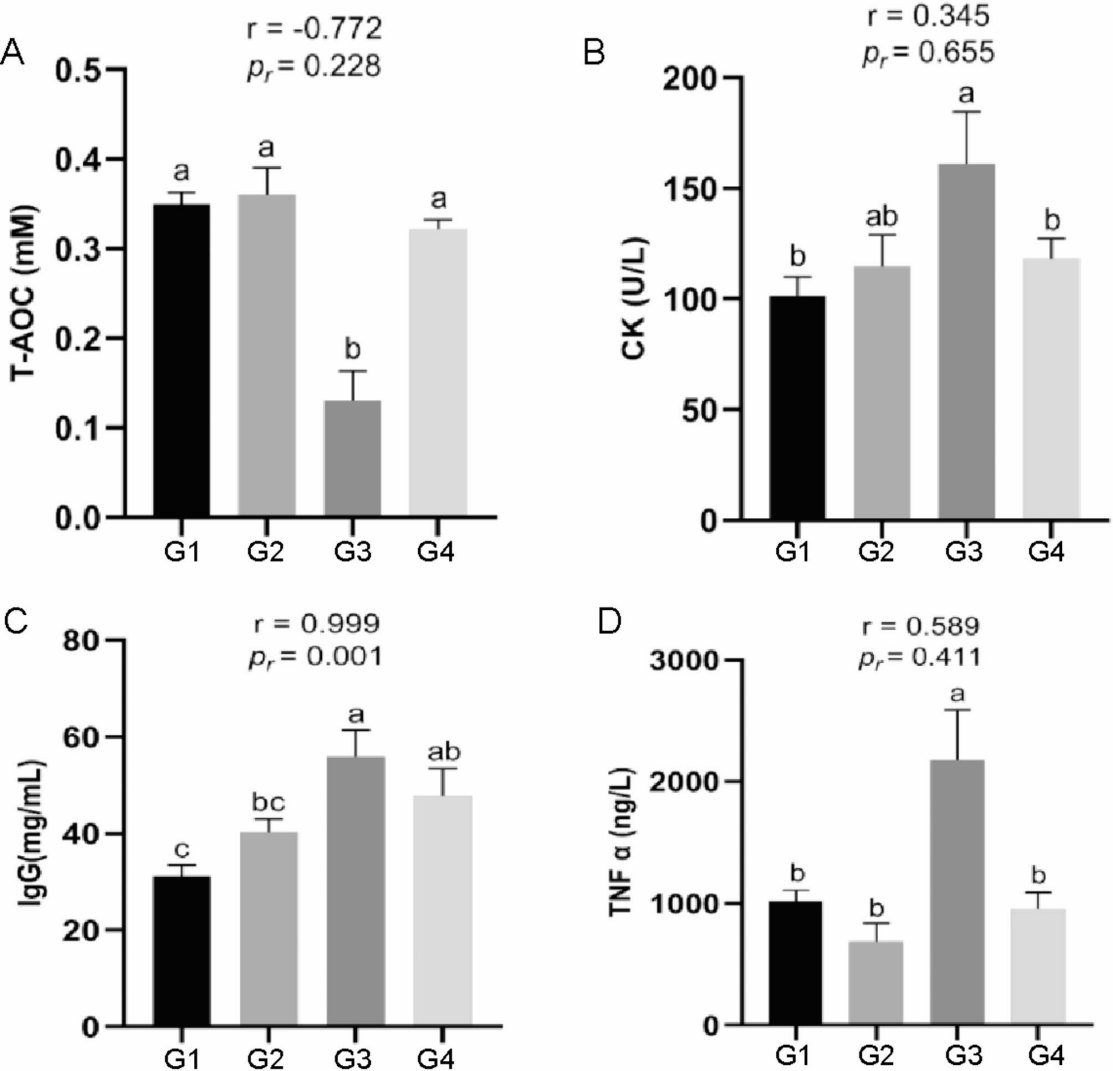
Effects of THI changes on oxidative stress and immune response in forest musk deer. (**A**) T-AOC concentration of serum; (**B**) CK concentration of serum; (**C**) IgG concentration of serum; (**D**) TNF α concentration of serum

Based on the results from Fig. 2, which show that stress hormones (Cortisol and CORT) are significantly higher in G3 compared to G1, G2, and G4, and that there are no significant differences in concentrations among G1, G2, and G4, we define G3 as the heat stress group and G1/G2/G4 as the normal groups. We found that the concentration of T-AOC in the blood of the heat-stress group was significantly lower than that of the normal group (Fig. 4A). CK reached the highest value in the heat-stress group and was significantly higher than that in G1 and G4 (Fig. 4B). There was a positive correlation between IgG and THI values, and the blood IgG concentration in the heat-stress group was significantly higher than that in G1 and G2 (Fig. 4C). Interestingly, contrary to the T-AOC results, the concentration of TNF-α in the heat-stress group was significantly higher than that in the normal group (Fig. 4D). Based on the above results, this suggests that during heat stress, the cells in forest musk deer may be damaged by oxidative stress, and there is a possibility of chronic inflammation in the body.

### Blood transcriptome changes in heat stress conditions

#### Analysis and statistics of RNA sequencing data

**Fig. 5:**
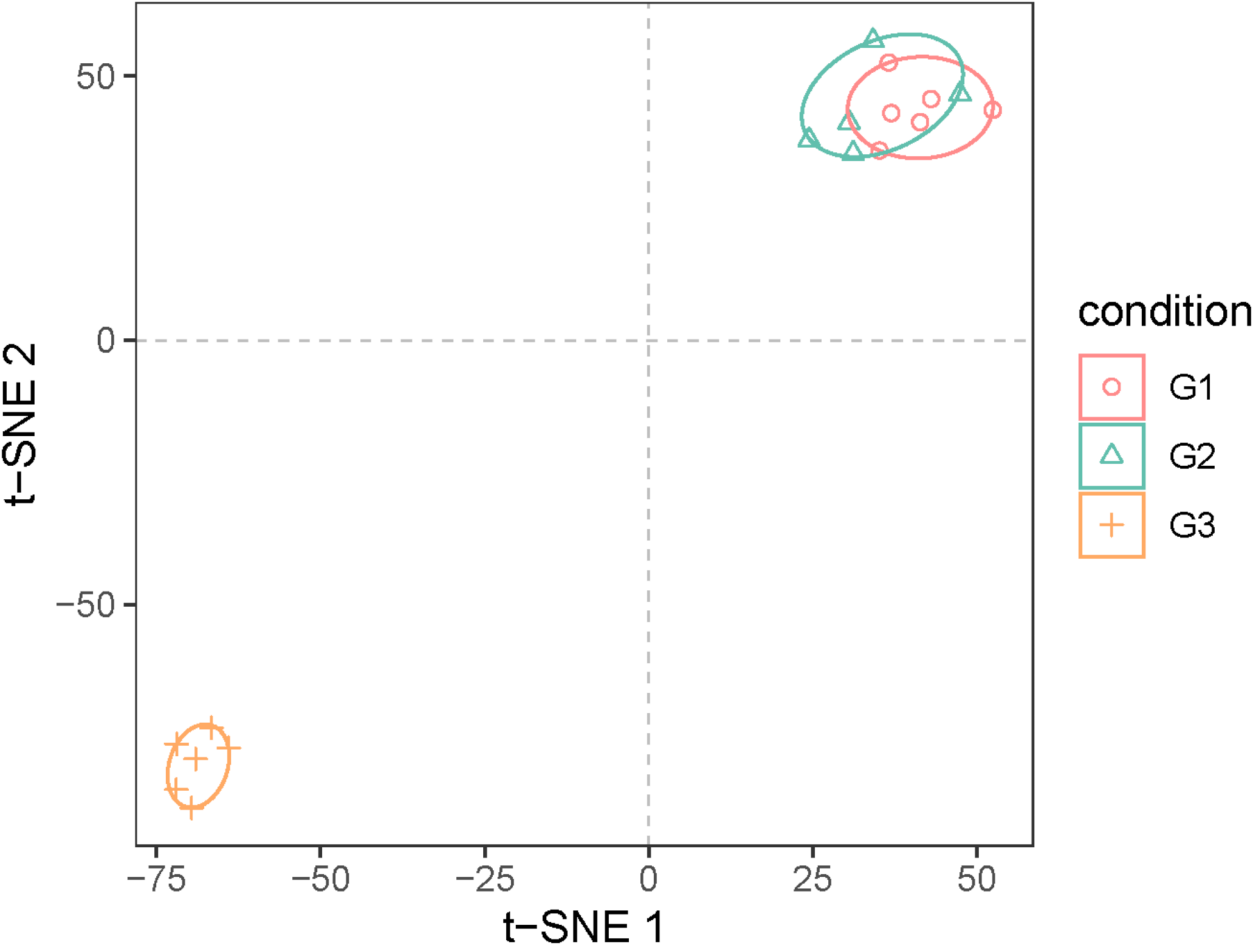
Principal component analysis of gene expression in G1, G2 and G3 groups

Our previous section determined that forest musk deer experience heat stress at G3. To gain a more comprehensive understanding of the molecular regulatory mechanisms involved in the response of forest musk deer to heat stress, RNA sequencing analysis was performed using 18 samples from G1 (*n* = 6), G2 (*n* = 6) and G3(*n* = 6). The same six individuals were selected as biological replicates in three periods. Prior to analysis, raw data underwent trimming. The 18 samples generated an average of 56 million reads each. RNA-seq analysis revealed that the average 94.93% of reads were mapped to the annotated forest musk deer assembly genome (unpublished). Detailed information regarding the raw reads can be found in Supplementary Table S2. Following the assembly process, the abundance read count value of each transcript was estimated. Principal component analysis demonstrated that the heat stress group and the normal group could be completely separated (Fig. 5).

### Identification of DEGs

**Fig. 6:**
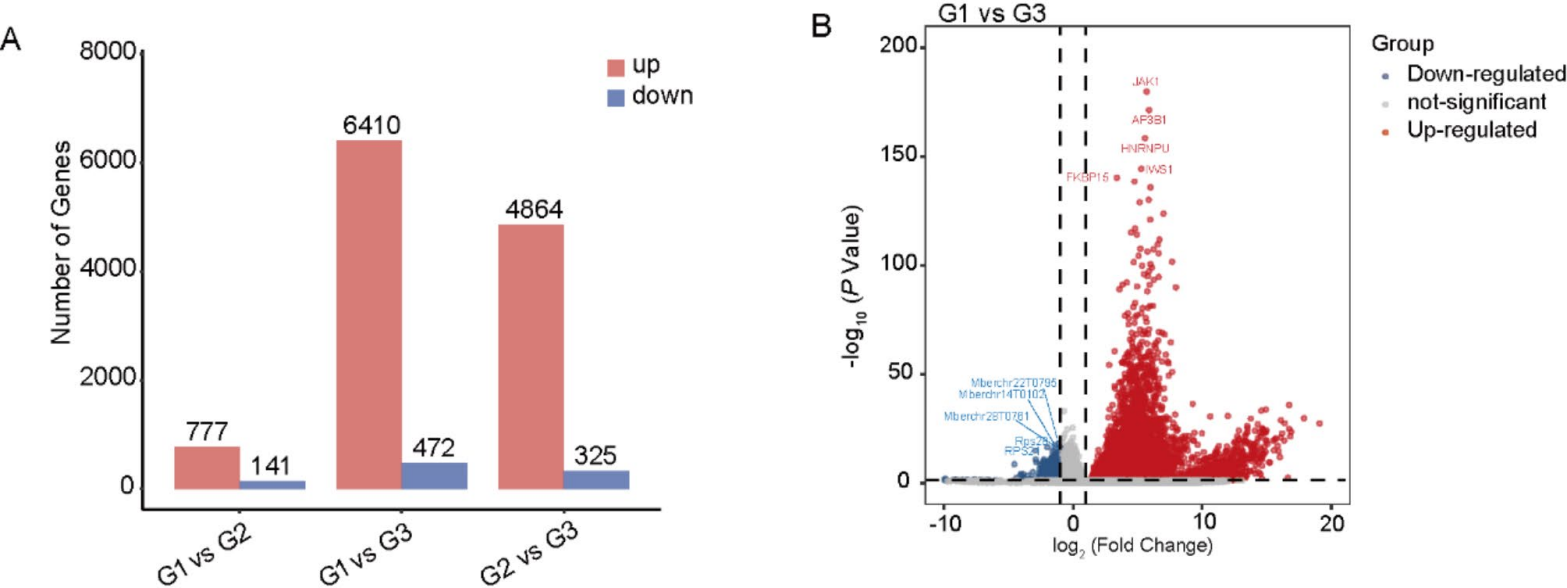
(**A**) The number of DEGs between groups was compared; (**B**) Volcano map of DEGs for G1 vs. G3

The DEGs was detected using DEseq2 library with *P* < 0.05 and|log2FC| ≥ 1 as the upregulated and downregulated genes out of 20,370 genes. We performed pairwise comparisons between G1, G2 and G3. The results revealed that the group without heat stress exhibited the fewest DEGs, whereas, the comparison between the heat stress group and the lowest THI group revealed the highest number of DEGs (Fig. 6A). Specifically, there were 472 up-regulated and 6,410 down-regulated genes in the G1 vs. G3 comparison. The DEGs and top 5 up-regulated and down-regulated genes are illustrated in Fig. 6B. Notably, the top-5 of DEGs in G1 vs. G3 were as follows: up-regulated genes included *JAK1*, *AP3B1*, *HNRNPU*, *IWS1*, *FKBP15*, while down-regulated genes comprised *RPS28*, *RPS21*, *RPL27A*, *RPS12* and *TMEM179B*. Furthermore, we identified 18 heat shock-related protein-coding genes among the DEGs, with HSPBP1 (*HSP70*) being significantly up-regulated in the G3. The expression results of the detected genes and their FDR values were provided in Supplementary Table S3.

### Function and pathway analysis of the DEGs

**Fig. 7:**
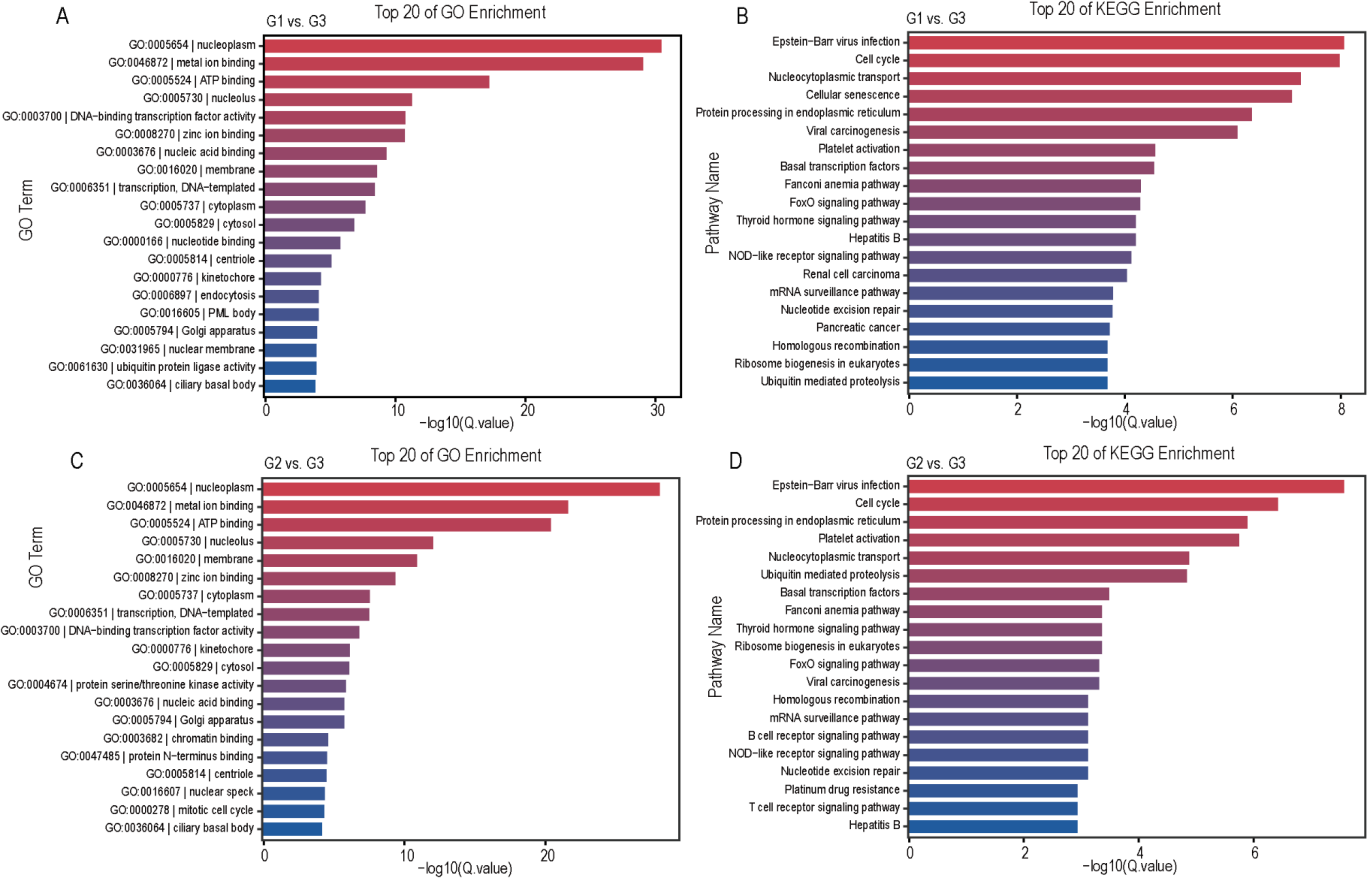
Top20 GO and KEGG enrichment analysis of heat-stressed and normal DEGs. (**A**) Top 20 GO enrichment of G1 vs. G3; (**B**) Top 20 KEGG enrichment of G1 vs. G3; (**C**) Top 20 GO enrichment of G2 vs. G3; (**D**) Top 20 KEGG enrichment of G2 vs. G3

To elucidate the specific biological processes enriched in forest musk deer under heat stress, we performed GO and KEGG pathway analysis for functional annotation using the identified 6882 (G1 vs. G3) and 5189 (G2 vs. G3) DEGs. The results showed 15 of the top 20 GO terms were identical between the two independent heat stress vs. normal comparison groups (Fig. 7A and C). Similarly, KEGG enrichment analysis demonstrated that 17 of the top 20 pathways were shared (Fig. 7B and D). The DEGs in the two comparison groups were predominantly enriched in biological processes related to oxidative stress, such as the FoxO signaling pathway, Immune response like the NOD-like receptor signaling pathway, and energy metabolism including protein processing in the endoplasmic reticulum, thyroid hormone signaling pathway, ribosome biogenesis in eukaryotes, and ubiquitin-mediated proteolysis. Based on the above results, we found that significantly enriched pathways are associated with changes in blood biochemical parameters. For instance, oxidative stress-related pathways may be key factors influencing the concentrations of T-AOC, CK, IgG, and TNF-α in the blood of forest musk deer, while energy metabolism-related pathways may play a critical role in affecting the concentrations of BUN and TG.

### Screening and trend analysis of co-expressed DEGs

**Fig. 8:**
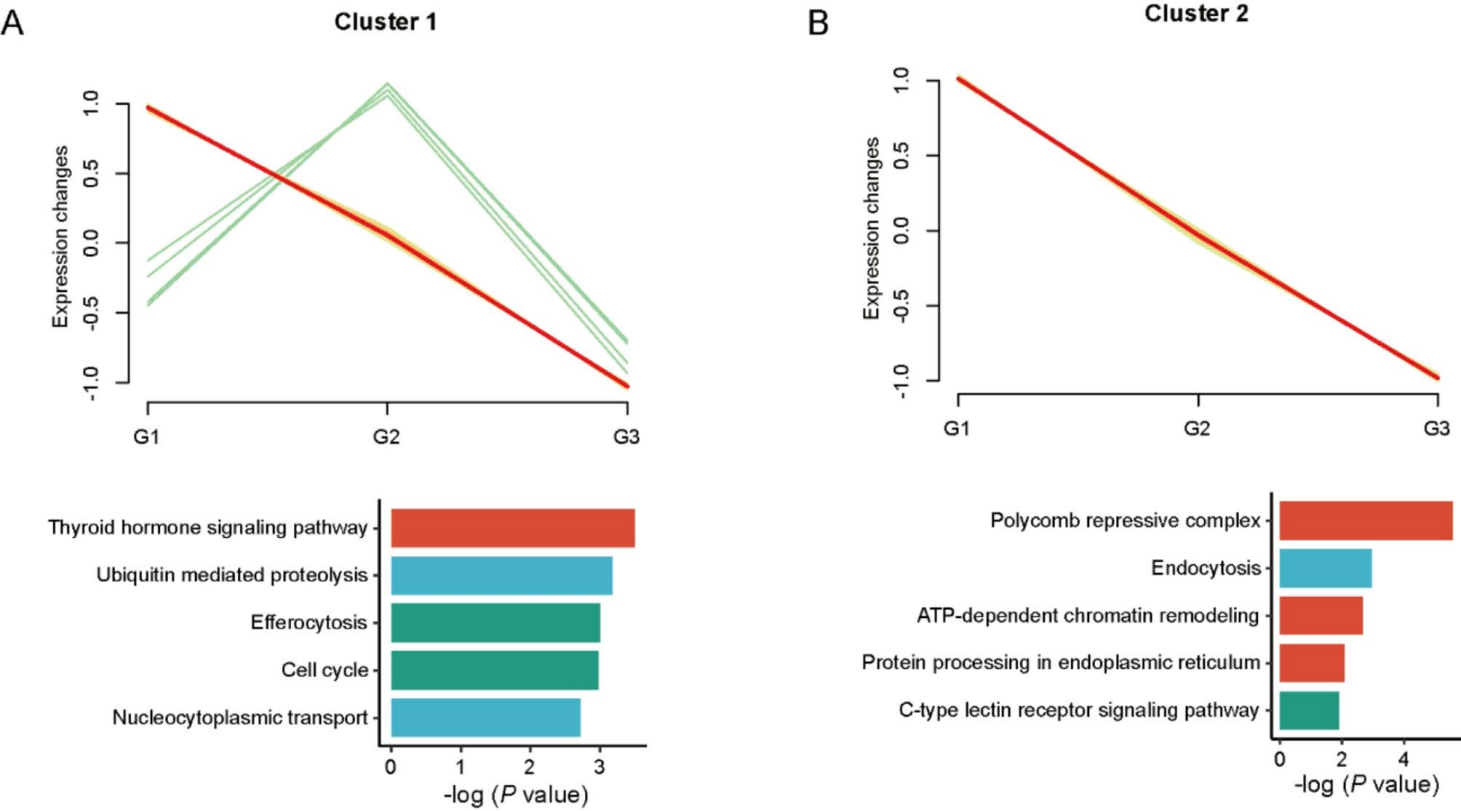
Trend analysis and pathway enrichment of co-expressed DEGs. (**A**) Expression trends and pathway enrichment of DEGs in cluster 1. (**B**) Expression trends and pathway enrichment of DEGs in cluster 2

In response to the chronic heat stress that farmed forest musk deer experience during THI fluctuations, we explored the changing expression patterns of DEGs and their molecular control mechanisms. We started by identifying 374 genes that were co-expressed across different conditions (G1vsG2, G1vsG3, and G2vsG3). Using the Mfuzz R package, we grouped these genes into four clusters based on their expression dynamics: cluster 1 (*n* = 150), cluster 2 (*n* = 146), cluster 3 (*n* = 74), and cluster 4 (*n* = 4) (Fig. 8 and S2). Clusters 1 and 2 showed decreasing expression trends, with genes in cluster 1 primarily enriched in thyroid hormone signaling and ubiquitin mediated proteolysis pathways (Fig. 8A), and genes in cluster 2 mainly associated with polycomb repressive complex and protein processing in endoplasmic reticulum pathways (Fig. 8B). Conversely, cluster 4 genes showed increasing expression, focusing on immune responses (Figure S2B). Notably, within cluster 1, genes *PDK4*, *KLF11*, *CD36*, and *CPT1A* initially increased but then decreased during heat stress, aligning with changes in blood triglyceride levels (Fig. 3D). These findings suggest that heat stress impacts energy metabolism and may trigger inflammation and immune system responses in forest musk deer.

### Gene regulatory network in forest musk deer under heat stress conditions

**Fig. 9:**
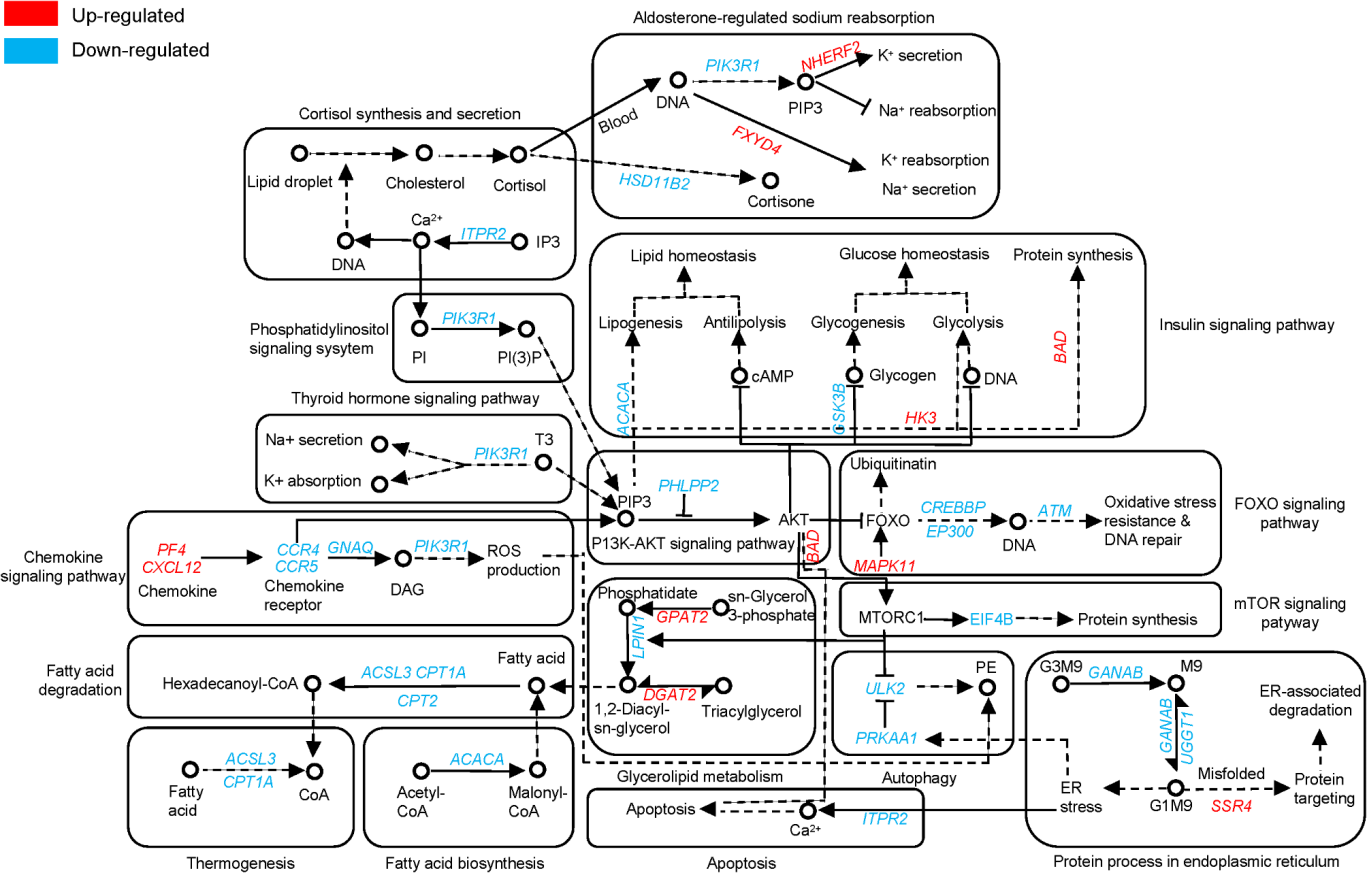
Gene-pathway mapping under heat stress. Red and blue indicate up-regulated and down-regulated genes of forest musk deer under heat stress conditions, respectively

To gain more insight into the mechanisms of gene regulation when forest musk deer were subjected to heat stress, we mapped the network of gene-regulated metabolic pathways based on the DEGs of G1 vs. G3 (Fig. 9). The results showed that high blood concentration of cortisol during heat stress activated the Aldosterone-regulated sodium reabsorption signaling pathway, which regulated intracellular and extracellular Na^+^ and K^+^ concentrations by up-regulating *NHERF2* and *FXYD4* to maintain electrolyte and body fluid homeostasis. The gene regulatory network also showed that *PIK3R1* was down-regulated in Aldosterone-regulated sodium reabsorption and Thyroid hormone signaling pathways to regulate intracellular and extracellular Na^+^ and K^+^ concentrations. Under the stimulation of membrane potential and hormones, the cAMP and Calcium signaling pathways are activated, and the cascade effect affects the downstream metabolic pathways, such as glucose metabolism, fatty acid metabolism, and signaling pathways related to immune pathways to adapt to the heat stress environment.

## Discussion

The aim of this study was to investigate the effects of heat stress on forest musk deer. Blood samples were collected in April, June, and July, which corresponds to the period of highest Temperature-Humidity Index (THI), aligning with local climatic conditions. The experimental process ensured that that THI was the sole influencing factor. The trend of serum physiological indices and transcriptome alterations were analyzed with THI increasing to its peak over the course of the year. The study firstly revealed heat stress affects the normal metabolism of forest musk deer and adversely affects their immune system.

### Effects of heat stress on physiological parameters of forest musk deer

Heat stress has profound effects on livestock and poultry production, disrupting normal hormone secretion in animals and impacting growth, metabolism, reproduction, and immunity [21–23]. In the studies, we first examined a significant increase in cortisol and corticosterone concentrations in the G3 group when THI reached 74.89, mirroring findings from previous studies on heat stress in other species [24–27]. Activation of the hypothalamic-pituitary-adrenal (HPA) axis is a known response to heat stress, leading to heightened circulating concentrations of cortisol, corticosterone, and adrenocorticotropic hormone (ACTH) [28, 29]. Therefore, based on these two stress-related hallmark indicators, combined with the physiological behaviors observed in forest musk deer during G3—such as increased respiratory rate, elevated water intake, and reduced appetite [30, 31]—compared to G1, G2, and G4, we conclude that forest musk deer exhibit heat stress responses when the THI reaches 74.89. Furthermore, the ion concentration in the blood serves as a reliable indicator of alterations in osmotic pressure within the body in response to heat stress. Barnes and colleagues, in their investigation into feed additives for mitigating heat stress in lambs, found that compared to the control group, the heat-stressed lambs showed significantly reduced blood sodium (Na+) levels and increased calcium (Ca2+) levels, while potassium (K+) levels remained statistically unchanged [32]. Arth and coworkers observed that chronic heat stress significantly elevated sodium (Na+) concentrations in pig blood, with a significant decrease in chloride (Cl-) levels during the same period, while potassium (K+) levels showed no significant fluctuation [33]. Our study partially aligns with Barnes et al.‘s findings, particularly the significant decrease in Na + levels in lambs during heat stress (Fig. 3B). We speculate that the discrepancies may be due to differences in species and experimental approaches. However, the consistent response to heat stress across species is evident. Overall, the lack of significant changes in K^+^, Ca^2+^, and Cl^−^ before and after heat stress may indicate thermal adaptation in musk deer exposed to prolonged high temperatures, while the marked decrease in Na^+^ concentration during heat stress may be attributed to increased water intake, perspiration, or urination for heat dissipation [34].

During episodes of heat stress in animals, the body initiates self-regulatory mechanisms to counter its effects, leading to significant alterations in nutrient metabolism [31]. Some studies have demonstrated that heat stress in lambs significantly reduces plasma BUN concentration while increasing TG concentration [35]. Similarly, pigs shown a significant decrease in BUN concentration during heat stress [36], consistent with our findings. However, we found that serum TG concentrations did not change regularly before and after heat stress, differing from findings in other species [35, 37]. We speculated that no significant correlation between THI and BUN and TG concentration might be attributed to self-regulation under prolonged chronic heat stress, which mitigate the impact of THI on protein and lipid metabolism. Consequently, further research involving acute heat stress treatments is warranted to elucidate the effects of heat stress on protein and lipid metabolism.

Moreover, heat stress is recognized as a catalyst for oxidative stress in domestic animals exposed to high temperature and humidity in summer. High temperature exposure leads to the generation of reactive oxygen species (ROS), leading to oxidative stress to damage cells [10]. Huang et al. found a significant reduction in total antioxidant capacity (T-AOC) in broilers exposed to heat stress [38], and Xie et al. found that chronic heat stress increased CK concentration in broilers, and MDA did not change significantly before and after heat stress [39], consistent with our findings (Figure S1C). Oxidative stress can damage the body and promote the occurrence of inflammation. High THI (80.3 ± 1.0) resulted in extremely significant increase in serum TNF-α concentration in dairy cows [40]. Previous studies have shown that high temperatures and excess ammonia elevate immunoglobulin G (IgG) concentration in laying hens [41]. The same results were obtained in our study. The increase of TNF-α and IgG concentrations implies heat stress leads to the occurrence of immune response in forest musk deer. Conversely, other immune proteins, such as IgA and IgM did not change significantly before and after heat stress (Figure S1E and S1F). We proposed that prolonged chronic heat stress might induce adaptive immunity [31]. However, high THI still triggers inflammation in forest musk deer, despite the immune system response to mitigate the negative effects of heat stress. Overall, the changes in plasma biochemical indicators due to heat stress in forest musk deer are not entirely consistent with those reported in other species. We speculate that this discrepancy may be related to species differences and variations in experimental treatment duration. We believe that prolonged exposure to an environment with gradually increasing THI has enhanced the adaptability of forest musk deer to high THI conditions. To gain a more comprehensive understanding of the molecular regulatory mechanisms involved in the response of forest musk deer to heat stress, we further analyzed the transcriptome profile of the blood of forest musk deer.

### Transcriptomic changes in the heat stress environment

In this study, we performed transcriptome analysis across three periods characterized by the continuous rise of THI to its peak, aiming to elucidate the regulatory mechanisms of DEGs in the context of heat stress. We performed comparative analysis between the heat stress and the normal group based on the serum biochemical indicators of forest musk deer examined previously. The comparison between G1 and G3 revealed 6,882 DEGs, with 6,410 up-regulated and 472 down-regulated genes. And the comparison between G2 and G3 identified 5,189 DEGs, comprising 4,864 up-regulated and 325 down-regulated genes. GO and KEGG analysis revealed that the two-comparison analysis had most of the same functions and pathways, prominently enriched in cell cycle regulation, nucleocytoplasmic transport, protein processing in the endoplasmic reticulum, and the thyroid hormone signaling pathway. Notably, we identified 18 genes annotated as heat shock proteins in G1 vs. G3, in which *HSP70* was significantly up-regulated in the heat stress group, consistent with its expression pattern in heat-stressed cattle [42]. Previous studies had linked protein processing in endoplasmic reticulum to regulation of *HSP70* [43]. Furthermore, *HSP70* enters the nucleus as a molecular chaperone under heat stress conditions, while certain regulators of the *HSP70* chaperone system accumulate in the nucleus to participate in nucleocytoplasmic transport, thereby protect cells from heat stress-induced damage, and aiding in their recovery from heat stress [44].

To understand the expression trend of DEGs during the THI rise, we identified co-expressed DEGs after pairwise comparisons of G1, G2, and G3, resulting in four distinct clusters by trend analysis, **o**ne cluster exhibited an upward expression trend and the other three clusters showing a downward expression trend. Clusters exhibiting decreased gene expression were notably enriched in pathways related to energy metabolism, protein metabolism and immune system, such as thyroid hormone signaling pathway, ATP-dependent chromatin remodeling, and protein metabolism, nucleocytoplasmic transport, ubiquitin mediated proteolysis, protein processing in endoplasmic reticulum, efferocytosis, C-type lectin receptor signaling pathway and other signaling pathways. Thyroid hormone plays a pivotal role in thermogenesis regulation in animal. Excessive thyroid hormone can inhibit the expression of HSP gene, exacerbating heat-induced cellular damage [45]. When cattle were exposed to acute heat stress, plasma concentrations of thyroid stimulating hormone (TSH), T3, and T4 decreased by 40%, 45.4%, and 25.9%, respectively [46]. These previous studies corroborate our findings that when forest musk deer are exposed to heat stress, the gene**s** expression enriched in the thyroid hormone signaling pathway **are** significantly down-regulated to reduce heat production (Fig. 8A). ATP-dependent chromatin remodeling is conserved in mammals and functions by altering nucleosome structure to provide cellular regulatory factors access to DNA. Mutations in the SWI-SNF complex of this pathway can lead to the activation of the *HSP70* gene [47]. Based on the significantly enriched pathway information of DEGs in Fig. 8A and B, we propose an intriguing hypothesis: the coordinated action of nucleocytoplasmic transport and ATP-dependent chromatin remodeling facilitates the entry of *HSP70* into the nucleus. Once inside the nucleus, *HSP70* enriches its associated regulatory factors of the molecular chaperone system. Simultaneously, ATP-dependent chromatin remodeling modifies nucleosome structure to allow regulatory factors to access DNA, thereby ensuring proper transcriptional regulation in forest musk deer cells under heat stress [44, 47]. However, the next step requires more in-depth cell experiments or the use of gene-edited mouse studies to validate this hypothesis. In addition, our results revealed that genes showing a downward trend with increasing THI were significantly enriched in protein metabolism pathways—Ubiquitin-mediated proteolysis and Protein processing in the endoplasmic reticulum (Fig. 8A and B). This implies that the concentration of the protein metabolite BUN in the blood would decrease, which aligns with our earlier findings that blood BUN levels in forest musk deer were significantly lower in the heat-stressed group compared to the normal group (Fig. 3C). Meanwhile, as the THI increased to levels causing heat stress in forest musk deer, the expression of genes enriched in pathways related to positive immune regulation, such as Efferocytosis and C-type lectin receptor signaling pathway, were significantly downregulated (Fig. 8A and B). This suggests a negative impact on the immune system of forest musk deer, potentially increasing their susceptibility to pathogens [10]. Additionally, genes in cluster 4 exhibited a consistently upregulated trend and were enriched in GO terms related to humoral immunity, such as Humoral immune response and Complement component C1 complex (Figure S2B). Our results have confirmed that forest musk deer experience oxidative stress and inflammation during heat stress (Fig. 4). Therefore, our findings align with those of Surinder et al., indicating that heat stress shifts the immune response from cell-mediated to humoral immunity [10]. This may impose an excessive burden on the humoral immune system of forest musk deer.

## Conclusions

In conclusion, this study analyzed the alterations in physiological and biochemical parameters and the molecular regulatory mechanisms of forest musk deer under different THI conditions. These analyses confirmed that forest musk deer experienced heat stress when the THI reaches 74.89. While heat stress minimally impact on internal homeostasis, it inevitably affect protein and lipid metabolism, induces oxidative stress, and adversely impacts the immune system of forest musk deer. Transcriptome analysis of blood showed that DEGs were enriched in signaling pathways related to cell metabolism, oxidative stress, and immune regulation. Notably, these genes enriched in protein metabolism, innate immunity, and antioxidation-related pathways were inhibited, whereas these genes related to humoral immunity displayed an upward trend with THI increasing. These findings provide theoretical support for the scientific management of forest musk deer to relieve heat stress damage, which is beneficial to the development of the forest musk deer breeding industry.

## Electronic supplementary material

Below is the link to the electronic supplementary material.

Supplementary Material 1: Figure S1: Effects of THI changes on Cl^−^(A), Ca^2+^(B), MDA(C), GSH-Px(D), IgG(E) and TNF-α(F) in serum of forestry musk deer; Figure S2: The down-regulated DEGs (A) and up-reulated DEGs (D) with THI-increasing

Supplementary Material 2: Table S1: The Td, RH and THI every 2 h interval in April, June, July and AguestTable S2: Sequencing data quality; Table S3: The DEGs and and their FDR values

## Data Availability

Sequence data that support the findings of this study have been deposited in the NCBI with the primary accession no.PRJNA1246736. (https://dataview.ncbi.nlm.nih.gov/object/PRJNA1246736).
