# Supplementary material for: The blood transcriptome of musk deer under heat stress condition reveals the regulatory mechanism of genes to maintain homeostasis metabolism
Source: BMC Genomics. 2025 Apr 24;26:400. doi: 10.1186/s12864-025-11577-y (PMC12023374; doi:10.1186/s12864-025-11577-y)
Supplement: Supplementary file 2 — Supplementary Material 2: Table S1: The Td, RH and THI every 2 h interval in April, June, July and AguestTable S2: Sequencing data quality; Table S3: The DEGs and and their FDR values [file 12864_2025_11577_MOESM2_ESM.pdf]

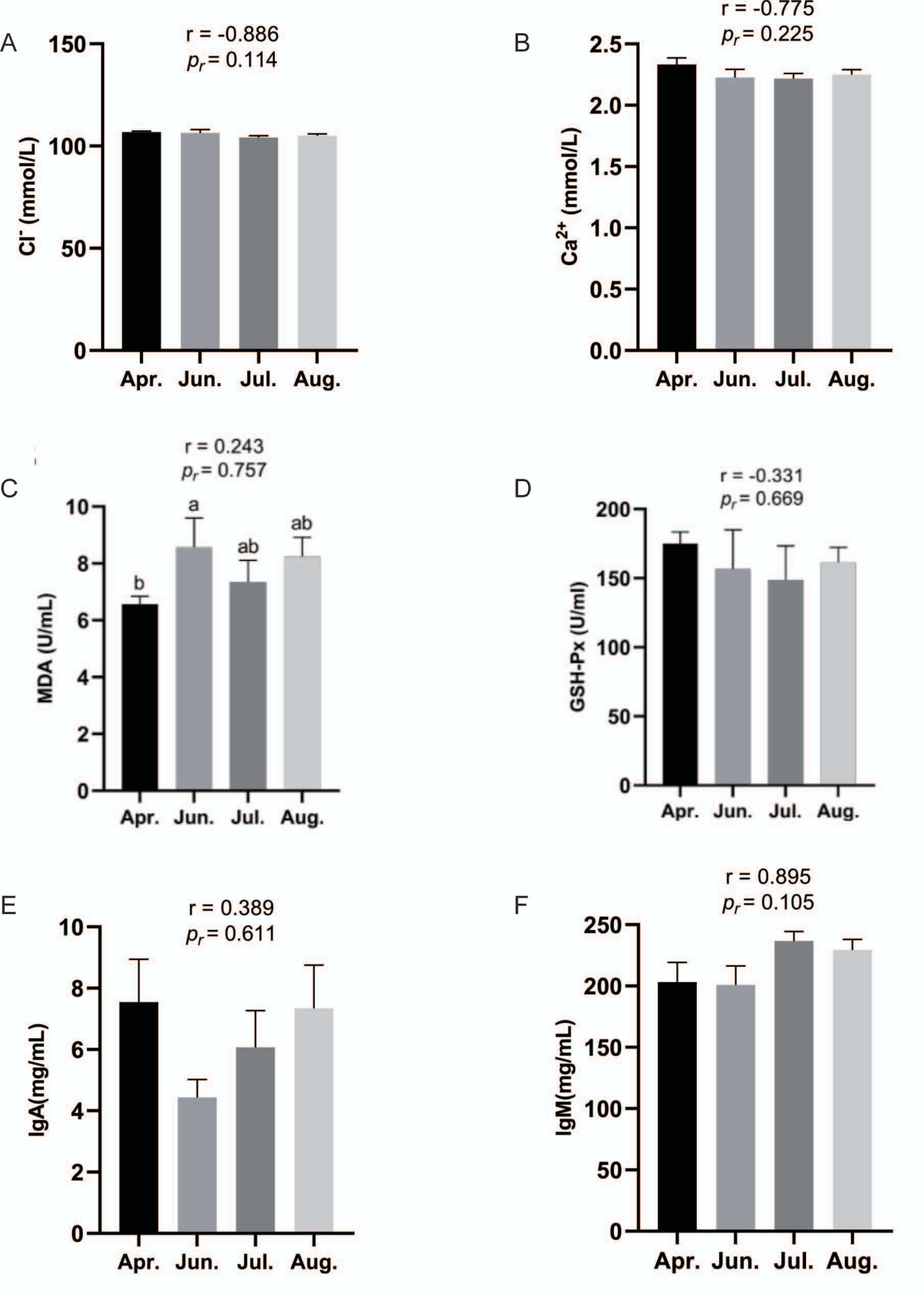

Figure S1 The effects of THI changes on the osmotic pressure, oxidative stress, and immune levels in forest musk deer. (A) Cl^- concentration of serum; (B) Ca^{2+} concentration of serum; (C) MDA concentration of serum; (D) GSH-Px concentration of serum; (E) IgA concentration of serum; (F) IgM concentration of serum.

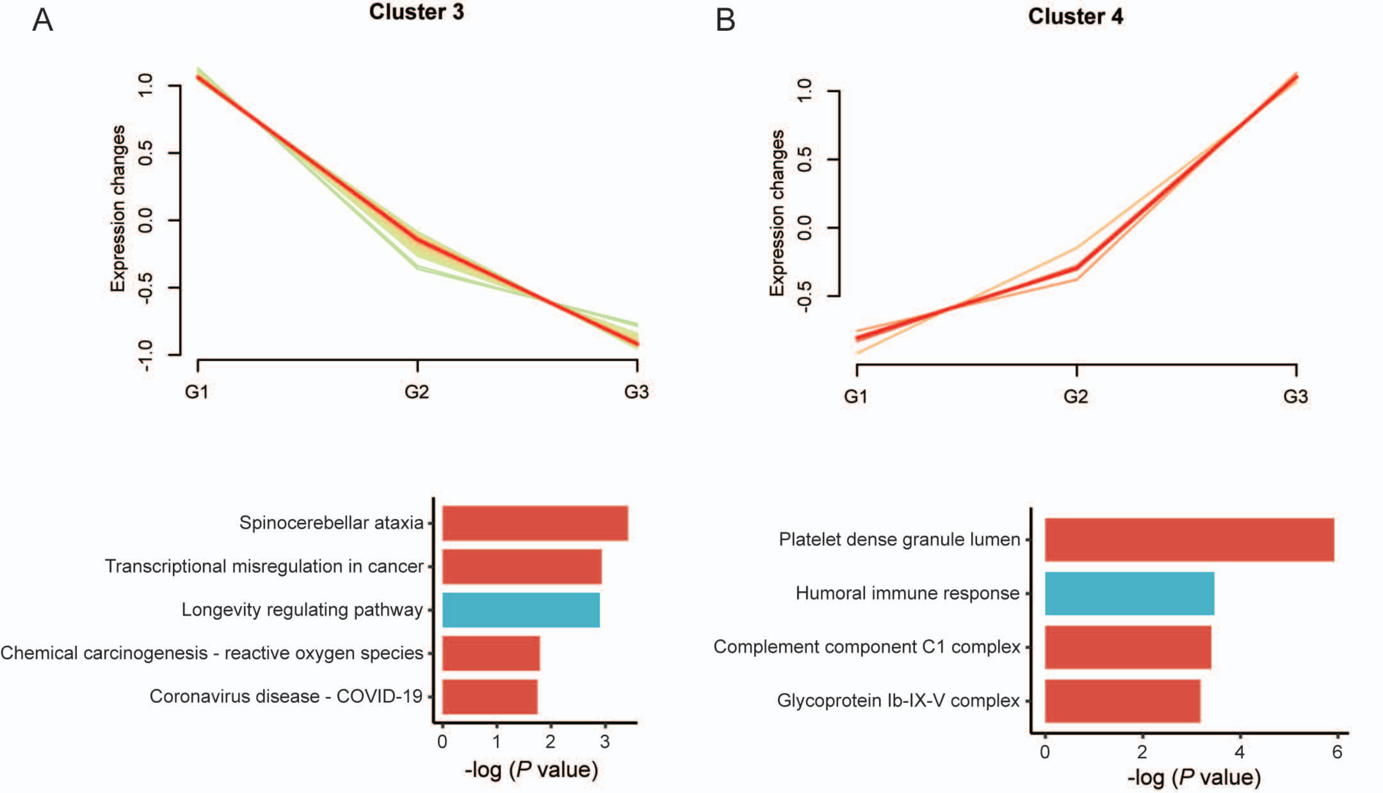

Figure S2 Trend analysis and pathway enrichment of co-expressed DEGs.

(A) Expression trends and pathway enrichment of DEGs in cluster 3; (B) Expression trends and pathway enrichment of DEGs in cluster 4.

Table S1:The Td, RH, and THI at 2-hours interval in April, June, July and August

April

Date	Time	Td(℃)	RH(%)	THI
2022/4/1	0:00:00	4.4693	99.8591	40.0586
2022/4/1	1:00:00	4.7875	99.4240	40.6726
2022/4/1	2:00:00	4.3408	99.8404	39.8294
2022/4/1	3:00:00	4.4634	99.7285	40.0609
2022/4/1	4:00:00	4.7316	99.4668	40.5681
2022/4/1	5:00:00	4.9756	96.5038	41.2839
2022/4/1	6:00:00	5.0181	95.9211	41.4133
2022/4/1	7:00:00	4.7671	96.4050	40.9252
2022/4/1	8:00:00	4.7671	94.7161	41.0870
2022/4/1	9:00:00	5.8024	91.9752	43.1309
2022/4/1	10:00:00	5.1724	92.5705	41.9923
2022/4/1	11:00:00	5.9610	91.5395	43.4403
2022/4/1	12:00:00	7.0409	86.8504	45.6374
2022/4/1	13:00:00	6.8603	88.4094	45.2188
2022/4/1	14:00:00	6.7125	89.7373	44.8680
2022/4/1	15:00:00	7.0011	90.6166	45.2935
2022/4/1	16:00:00	6.8576	91.2569	45.0004
2022/4/1	17:00:00	5.2654	96.6985	41.7777
2022/4/1	18:00:00	5.5734	95.6347	42.4155
2022/4/1	19:00:00	6.3249	96.2573	43.6856
2022/4/1	20:00:00	6.8915	92.6026	44.9578
2022/4/1	21:00:00	7.0393	92.6476	45.2097
2022/4/1	22:00:00	5.8868	93.3354	43.1608
2022/4/1	23:00:00	6.2077	89.3526	44.0421
2022/4/2	0:00:00	6.3115	86.0940	44.4803
2022/4/2	1:00:00	6.6179	83.2491	45.2100
2022/4/2	2:00:00	6.4281	85.2544	44.7408
2022/4/2	3:00:00	5.6132	83.5478	43.5421
2022/4/2	4:00:00	5.6723	82.3510	43.7429
2022/4/2	5:00:00	5.9325	83.2469	44.0902
2022/4/2	6:00:00	5.4637	83.1732	43.3308
2022/4/2	7:00:00	5.3186	89.5503	42.5176
2022/4/2	8:00:00	5.3009	87.1043	42.7089
2022/4/2	9:00:00	8.7777	77.0954	49.0848
2022/4/2	10:00:00	8.1461	81.7319	47.8020
2022/4/2	11:00:00	7.6446	82.4292	46.9430
2022/4/2	12:00:00	10.0592	75.9703	51.1498
2022/4/2	13:00:00	10.2441	75.6621	51.4515
2022/4/2	14:00:00	9.9334	77.2311	50.8970
2022/4/2	15:00:00	10.1302	74.1895	51.3367

June

Date	Time	Td(℃)	RH(%)	THI
2022/6/1	0:00:00	17.9708	86.6927	63.8830
2022/6/1	1:00:00	18.1998	84.7328	64.1921
2022/6/1	2:00:00	18.1160	84.4879	64.0449
2022/6/1	3:00:00	17.5430	87.8982	63.2061
2022/6/1	4:00:00	17.8257	86.0334	63.6188
2022/6/1	5:00:00	18.2681	83.5319	64.2592
2022/6/1	6:00:00	17.7682	85.3042	63.4992
2022/6/1	7:00:00	17.2457	91.7026	62.8121
2022/6/1	8:00:00	17.3865	90.5787	63.0213
2022/6/1	9:00:00	19.0626	85.0224	65.6279
2022/6/1	10:00:00	18.9503	86.6986	65.5171
2022/6/1	11:00:00	18.8465	87.6268	65.3845
2022/6/1	12:00:00	20.5360	83.5848	67.9749
2022/6/1	13:00:00	20.4844	83.8369	67.9054
2022/6/1	14:00:00	20.3425	84.2697	67.6980
2022/6/1	15:00:00	21.0671	80.2170	68.6237
2022/6/1	16:00:00	21.2940	78.4274	68.8663
2022/6/1	17:00:00	18.9116	85.8908	65.4168
2022/6/1	18:00:00	19.0271	85.8678	65.6077
2022/6/1	19:00:00	18.9589	88.7376	65.6226
2022/6/1	20:00:00	20.1291	84.7410	67.3736
2022/6/1	21:00:00	19.9754	85.5871	67.1665
2022/6/1	22:00:00	18.2031	93.6278	64.5284
2022/6/1	23:00:00	17.5919	94.5359	63.4951
2022/6/2	0:00:00	16.8151	96.1767	62.1775
2022/6/2	1:00:00	16.5270	94.7979	61.6413
2022/6/2	2:00:00	17.0785	92.3683	62.5423
2022/6/2	3:00:00	16.7974	91.8993	62.0466
2022/6/2	4:00:00	17.2887	88.6267	62.7994
2022/6/2	5:00:00	17.1865	85.1915	62.5338
2022/6/2	6:00:00	17.7698	82.8977	63.4226
2022/6/2	7:00:00	17.8230	85.0907	63.5828
2022/6/2	8:00:00	17.5596	88.2652	63.2454
2022/6/2	9:00:00	20.6398	80.5131	67.9564
2022/6/2	10:00:00	19.6931	87.0420	66.7743
2022/6/2	11:00:00	19.3228	89.2242	66.2606
2022/6/2	12:00:00	21.1209	81.4652	68.7925
2022/6/2	13:00:00	20.7521	83.3062	68.3113
2022/6/2	14:00:00	20.4177	84.4006	67.8295
2022/6/2	15:00:00	20.6220	84.5073	68.1722

July

Date	Time	Td(℃)	RH(%)	THI
2022/7/1	0:00:00	19.6206	56.8820	65.1075
2022/7/1	1:00:00	19.5093	57.3831	64.9798
2022/7/1	2:00:00	19.1674	57.8664	64.5313
2022/7/1	3:00:00	17.7531	61.6262	62.6987
2022/7/1	4:00:00	17.3833	62.6871	62.2043
2022/7/1	5:00:00	18.4455	60.6798	63.6444
2022/7/1	6:00:00	19.3824	60.4474	64.9548
2022/7/1	7:00:00	19.6571	64.4156	65.5465
2022/7/1	8:00:00	19.8910	54.2566	65.3372
2022/7/1	9:00:00	21.4875	50.7106	67.2407
2022/7/1	10:00:00	20.5538	55.8839	66.3285
2022/7/1	11:00:00	19.2948	64.8165	65.0412
2022/7/1	12:00:00	19.2491	67.8934	65.1213
2022/7/1	13:00:00	19.1588	71.8980	65.1743
2022/7/1	14:00:00	19.3733	71.7397	65.4929
2022/7/1	15:00:00	20.3866	67.5510	66.7870
2022/7/1	16:00:00	21.6724	62.2638	68.3100
2022/7/1	17:00:00	19.0180	79.0381	65.2832
2022/7/1	18:00:00	18.7578	81.5940	64.9781
2022/7/1	19:00:00	18.5670	85.5059	64.8290
2022/7/1	20:00:00	19.2298	82.1685	65.7688
2022/7/1	21:00:00	19.2212	83.4999	65.8178
2022/7/1	22:00:00	17.7338	88.0493	63.5317
2022/7/1	23:00:00	17.6569	86.0227	63.3379
2022/7/2	0:00:00	17.4499	85.1049	62.9667
2022/7/2	1:00:00	16.3980	87.8628	61.2816
2022/7/2	2:00:00	17.2575	83.5293	62.6048
2022/7/2	3:00:00	16.9307	84.4968	62.0936
2022/7/2	4:00:00	16.7796	83.6538	61.8254
2022/7/2	5:00:00	16.2588	85.2093	61.0001
2022/7/2	6:00:00	16.4426	86.8207	61.3360
2022/7/2	7:00:00	16.9914	90.7809	62.3521
2022/7/2	8:00:00	17.5741	88.5835	63.2797
2022/7/2	9:00:00	19.1395	84.9043	65.7494
2022/7/2	10:00:00	18.4917	90.0277	64.8855
2022/7/2	11:00:00	17.9262	90.0717	63.9250
2022/7/2	12:00:00	19.0583	85.1932	65.6286
2022/7/2	13:00:00	18.9798	84.8083	65.4816
2022/7/2	14:00:00	18.7740	86.5484	65.2165
2022/7/2	15:00:00	19.3109	84.4317	66.0096

August

Date	Time	Td(℃)	RH(%)	THI
2022/8/1	0:00:00	19.4784	83.2417	66.2259
2022/8/1	1:00:00	19.2235	82.8633	65.7916
2022/8/1	2:00:00	19.6644	81.3005	66.4295
2022/8/1	3:00:00	19.5267	82.1561	66.2503
2022/8/1	4:00:00	19.8745	81.4386	66.7763
2022/8/1	5:00:00	20.5465	81.6086	67.8727
2022/8/1	6:00:00	20.4379	83.1940	67.7911
2022/8/1	7:00:00	20.3922	85.8224	67.8712
2022/8/1	8:00:00	19.5649	86.5241	66.5337
2022/8/1	9:00:00	21.5270	77.7148	69.1861
2022/8/1	10:00:00	21.2674	80.4816	68.9629
2022/8/1	11:00:00	21.1126	82.2377	68.8301
2022/8/1	12:00:00	21.1814	83.0695	68.9973
2022/8/1	13:00:00	20.6508	83.6593	68.1674
2022/8/1	14:00:00	19.7966	87.5274	66.9730
2022/8/1	15:00:00	19.7143	87.6667	66.8424
2022/8/1	16:00:00	20.0799	88.1759	67.4841
2022/8/1	17:00:00	19.7337	86.7437	66.8265
2022/8/1	18:00:00	20.2729	85.5936	67.6599
2022/8/1	19:00:00	20.5540	81.7401	67.8928
2022/8/1	20:00:00	21.0599	77.2106	68.4153
2022/8/1	21:00:00	20.3922	78.8672	67.4617
2022/8/1	22:00:00	18.9187	84.0700	65.3481
2022/8/1	23:00:00	18.7817	84.1482	65.1264
2022/8/2	0:00:00	18.5865	85.4921	64.8608
2022/8/2	1:00:00	18.5199	84.8548	64.7247
2022/8/2	2:00:00	18.7096	83.2540	64.9702
2022/8/2	3:00:00	17.7646	87.0686	63.5512
2022/8/2	4:00:00	17.8855	88.0274	63.7861
2022/8/2	5:00:00	18.2898	83.8180	64.3056
2022/8/2	6:00:00	18.2898	85.0386	64.3521
2022/8/2	7:00:00	17.8216	89.1951	63.7176
2022/8/2	8:00:00	17.6555	89.6510	63.4508
2022/8/2	9:00:00	21.5980	77.3082	69.2694
2022/8/2	10:00:00	21.0223	81.2565	68.6195
2022/8/2	11:00:00	20.6992	82.4160	68.1697
2022/8/2	12:00:00	22.8796	74.2644	71.0341
2022/8/2	13:00:00	23.3107	73.9675	71.6743
2022/8/2	14:00:00	23.0951	74.8948	71.4212
2022/8/2	15:00:00	23.9972	72.3967	72.5844

2022/4/2	16:00:00	9.9420	77.0556	50.9184	2022/6/2	16:00:00	20.2194	86.4480	67.6201	2022/7/2	16:00:00	19.3867	85.0465	66.1645	2022/8/2	16:00:00	23.9816	72.9391	72.6118
2022/4/2	17:00:00	7.1204	86.0566	45.8278	2022/6/2	17:00:00	18.3127	93.1785	64.7016	2022/7/2	17:00:00	18.0671	92.0603	64.2360	2022/8/2	17:00:00	20.5368	81.9456	67.8773
2022/4/2	18:00:00	7.8913	85.3632	47.1539	2022/6/2	18:00:00	18.5863	90.6412	65.0716	2022/7/2	18:00:00	18.3315	90.8832	64.6459	2022/8/2	18:00:00	20.4820	84.1636	67.9210
2022/4/2	19:00:00	8.8992	85.8094	48.7976	2022/6/2	19:00:00	19.5980	88.6795	66.6988	2022/7/2	19:00:00	18.7653	88.6673	65.2928	2022/8/2	19:00:00	20.9217	89.1547	68.9637
2022/4/2	20:00:00	9.5131	82.3170	49.9868	2022/6/2	20:00:00	20.0877	87.7419	67.4730	2022/7/2	20:00:00	18.5713	91.8767	65.0964	2022/8/2	20:00:00	20.9766	87.8568	68.9725
2022/4/2	21:00:00	9.2276	84.6572	49.4021	2022/6/2	21:00:00	19.5303	92.6571	66.7848	2022/7/2	21:00:00	18.3111	92.3019	64.6653	2022/8/2	21:00:00	21.2271	90.4054	69.5645
2022/4/2	22:00:00	7.5215	89.4312	46.2630	2022/6/2	22:00:00	18.3466	95.9446	64.8672	2022/7/2	22:00:00	17.3156	97.0860	63.0852	2022/8/2	22:00:00	19.1671	95.2181	66.2772
2022/4/2	23:00:00	7.7005	90.4407	46.4991	2022/6/2	23:00:00	18.6955	93.8470	65.3929	2022/7/2	23:00:00	17.9456	95.4331	64.1438	2022/8/2	23:00:00	19.2321	93.1687	66.2941
2022/4/3	0:00:00	7.4640	92.0024	45.9878	2022/6/3	0:00:00	18.5863	93.5730	65.1919	2022/7/3	0:00:00	18.2074	94.5621	64.5707	2022/8/3	0:00:00	18.9053	93.2044	65.7294
2022/4/3	1:00:00	6.8823	93.1246	44.9029	2022/6/3	1:00:00	19.2018	89.8031	66.0831	2022/7/3	1:00:00	18.4412	92.2324	64.8868	2022/8/3	1:00:00	18.3812	93.1665	64.8198
2022/4/3	2:00:00	6.8619	92.5489	44.9108	2022/6/3	2:00:00	19.3405	87.8728	66.2251	2022/7/3	2:00:00	18.0950	91.4086	64.2605	2022/8/3	2:00:00	18.2753	93.2896	64.6410
2022/4/3	3:00:00	6.3840	94.6038	43.9219	2022/6/3	3:00:00	18.9503	87.0454	65.5326	2022/7/3	3:00:00	16.5238	95.2796	61.6456	2022/8/3	3:00:00	18.0737	93.7722	64.3088
2022/4/3	4:00:00	6.6071	92.0002	44.5135	2022/6/3	4:00:00	18.9029	85.7309	65.3955	2022/7/3	4:00:00	16.9957	89.6426	62.3307	2022/8/3	4:00:00	18.1038	91.4805	64.2782
2022/4/3	5:00:00	5.9728	84.7153	44.0330	2022/6/3	5:00:00	19.2653	85.6213	65.9912	2022/7/3	5:00:00	17.1129	83.6121	62.3703	2022/8/3	5:00:00	19.0273	90.2253	65.8057
2022/4/3	6:00:00	6.4593	85.3948	44.7813	2022/6/3	6:00:00	19.6738	85.7699	66.6761	2022/7/3	6:00:00	18.1042	80.2239	63.8710	2022/8/3	6:00:00	19.0311	91.1775	65.8554
2022/4/3	7:00:00	6.0615	91.0551	43.6530	2022/6/3	7:00:00	19.9619	81.8511	66.9401	2022/7/3	7:00:00	17.9235	83.6233	63.6983	2022/8/3	7:00:00	19.8971	87.0432	67.1154
2022/4/3	8:00:00	5.8674	96.8056	42.8326	2022/6/3	8:00:00	19.0626	85.2413	65.6379	2022/7/3	8:00:00	17.8730	88.8091	63.7916	2022/8/3	8:00:00	19.9987	81.7197	66.9925
2022/4/3	9:00:00	9.1239	83.8663	49.2728	2022/6/3	9:00:00	21.2004	80.1022	68.8299	2022/7/3	9:00:00	21.5272	76.2127	69.0811	2022/8/3	9:00:00	23.4784	74.6288	71.9921
2022/4/3	10:00:00	8.4111	86.6663	47.9364	2022/6/3	10:00:00	20.6667	82.6684	68.1323	2022/7/3	10:00:00	21.5719	79.8610	69.4083	2022/8/3	10:00:00	23.1478	77.3643	71.7157
2022/4/3	11:00:00	7.7612	91.4509	46.5358	2022/6/3	11:00:00	20.7731	81.7409	68.2475	2022/7/3	11:00:00	21.2520	82.8759	69.0996	2022/8/3	11:00:00	22.3705	79.7501	70.6779
2022/4/3	12:00:00	8.3574	88.7110	47.7235	2022/6/3	12:00:00	22.8518	73.3923	70.9186	2022/7/3	12:00:00	23.2216	76.0773	71.7202	2022/8/3	12:00:00	24.6444	74.9082	73.8262
2022/4/3	13:00:00	8.1902	89.3363	47.4026	2022/6/3	13:00:00	22.7034	74.3754	70.7709	2022/7/3	13:00:00	23.6963	75.0266	72.3659	2022/8/3	13:00:00	24.4063	74.7854	73.4446
2022/4/3	14:00:00	8.5573	88.8410	48.0535	2022/6/3	14:00:00	22.7394	75.8027	70.9438	2022/7/3	14:00:00	23.2630	76.6242	71.8326	2022/8/3	14:00:00	23.8445	75.2661	72.6184
2022/4/3	15:00:00	7.3118	93.1430	45.6455	2022/6/3	15:00:00	22.8706	76.9821	71.2469	2022/7/3	15:00:00	24.4451	73.1638	73.3442	2022/8/3	15:00:00	24.2800	74.6384	73.2344
2022/4/3	16:00:00	7.3070	91.4955	45.7535	2022/6/3	16:00:00	22.5540	78.1759	70.8450	2022/7/3	16:00:00	24.4467	74.1837	73.4476	2022/8/3	16:00:00	24.3977	74.5322	73.4063
2022/4/3	17:00:00	6.0335	94.9115	43.2841	2022/6/3	17:00:00	20.8440	87.5038	68.7275	2022/7/3	17:00:00	22.6502	81.3712	71.2570	2022/8/3	17:00:00	22.9022	79.4018	71.4992
2022/4/3	18:00:00	6.8501	95.8334	44.6434	2022/6/3	18:00:00	20.2904	85.9509	67.7096	2022/7/3	18:00:00	22.5115	82.1549	71.0955	2022/8/3	18:00:00	21.7001	81.6426	69.7416
2022/4/3	19:00:00	7.4478	94.3775	45.7955	2022/6/3	19:00:00	20.5333	85.7869	68.1032	2022/7/3	19:00:00	22.7690	84.9495	71.7438	2022/8/3	19:00:00	21.8335	89.9684	70.5664
2022/4/3	20:00:00	7.7478	92.9004	46.4167	2022/6/3	20:00:00	20.7403	84.3011	68.3540	2022/7/3	20:00:00	23.0528	85.5616	72.2646	2022/8/3	20:00:00	22.0399	88.9947	70.8442
2022/4/3	21:00:00	7.6623	94.2997	46.1749	2022/6/3	21:00:00	21.1563	81.9816	68.8841	2022/7/3	21:00:00	22.0051	87.5634	70.6783	2022/8/3	21:00:00	22.6495	84.4103	71.5027
2022/4/3	22:00:00	7.0882	93.8626	45.2057	2022/6/3	22:00:00	19.1545	86.7453	65.8600	2022/7/3	22:00:00	19.4206	95.1176	66.7166	2022/8/3	22:00:00	21.1099	88.5387	69.2415
2022/4/3	23:00:00	7.1844	92.3985	45.4783	2022/6/3	23:00:00	19.7372	83.5604	66.6656	2022/7/3	23:00:00	19.1190	93.5175	66.1143	2022/8/3	23:00:00	21.4711	85.5146	69.6404
2022/4/4	0:00:00	6.6829	92.8887	44.5756	2022/6/4	0:00:00	19.2282	87.9045	66.0379	2022/7/4	0:00:00	18.2842	94.5302	64.7037	2022/8/4	0:00:00	21.5529	85.3375	69.7633
2022/4/4	1:00:00	6.3485	91.4760	44.1106	2022/6/4	1:00:00	18.9546	89.7684	65.6614	2022/7/4	1:00:00	18.1515	93.4650	64.4328	2022/8/4	1:00:00	21.3943	85.3310	69.5004
2022/4/4	2:00:00	6.8764	89.0875	45.1951	2022/6/4	2:00:00	19.1320	89.5779	65.9539	2022/7/4	2:00:00	17.6865	93.3099	63.6210	2022/8/4	2:00:00	20.7777	87.7467	68.6315
2022/4/4	3:00:00	6.6356	86.3440	44.9998	2022/6/4	3:00:00	18.2622	88.6787	64.4441	2022/7/4	3:00:00	16.9661	92.3125	62.3472	2022/8/4	3:00:00	20.1799	87.9303	67.6385
2022/4/4	4:00:00	6.3216	88.8665	44.2743	2022/6/4	4:00:00	18.5181	85.6248	64.7528	2022/7/4	4:00:00	16.9366	87.9099	62.1876	2022/8/4	4:00:00	20.7970	84.5850	68.4652
2022/4/4	5:00:00	6.4818	91.3110	44.3523	2022/6/4	5:00:00	18.3493	93.6230	64.7822	2022/7/4	5:00:00	18.8449	77.3786	64.9353	2022/8/4	5:00:00	21.4658	78.3199	69.1313
2022/4/4	6:00:00	6.5420	89.7784	44.5754	2022/6/4	6:00:00	18.5213	93.8546	65.0903	2022/7/4	6:00:00	18.8304	77.9139	64.9357	2022/8/4	6:00:00	21.5690	76.2960	69.1522
2022/4/4	7:00:00	6.6431	88.6959	44.8307	2022/6/4	7:00:00	18.5138	93.3464	65.0567	2022/7/4	7:00:00	19.4502	80.0700	66.0226	2022/8/4	7:00:00	20.3546	86.8599	67.8695
2022/4/4	8:00:00	6.5259	93.9575	44.2204	2022/6/4	8:00:00	18.7740	93.4419	65.5120	2022/7/4	8:00:00	19.7668	81.9551	66.6294	2022/8/4	8:00:00	20.3046	88.6426	67.8894
2022/4/4	9:00:00	7.4360	87.6163	46.2440	2022/6/4	9:00:00	19.3099	91.8352	66.3645	2022/7/4	9:00:00	24.0559	72.5186	72.6856	2022/8/4	9:00:00	21.4641	85.7733	69.6468
2022/4/4	10:00:00	7.1591	90.5652	45.5669	2022/6/4	10:00:00	18.1305	93.1389	64.3845	2022/7/4	10:00:00	23.9951	75.4307	72.8682	2022/8/4	10:00:00	21.0733	83.2590	68.8333
2022/4/4	11:00:00	6.9329	93.9805	44.9268	2022/6/4	11:00:00	18.0138	94.2708	64.2225	2022/7/4	11:00:00	23.6355	78.6745	72.6035	2022/8/4	11:00:00	21.1938	86.1986	69.2266

2022/4/4	12:00:00	7.0189	95.2527	44.9829
2022/4/4	13:00:00	6.7732	98.2009	44.3284
2022/4/4	14:00:00	6.8587	97.1610	44.5588
2022/4/4	15:00:00	6.7377	96.0709	44.4277
2022/4/4	16:00:00	7.4225	92.7161	45.8669
2022/4/4	17:00:00	6.6754	98.1517	44.1578
2022/4/4	18:00:00	6.8780	99.6439	44.4071
2022/4/4	19:00:00	7.4446	99.4886	45.4357
2022/4/4	20:00:00	7.5483	97.0475	45.7886
2022/4/4	21:00:00	7.7080	94.0838	46.2690
2022/4/4	22:00:00	5.9432	95.1581	43.1053
2022/4/4	23:00:00	5.2992	92.6835	42.2011
2022/4/5	0:00:00	5.1794	89.5386	42.2824
2022/4/5	1:00:00	5.1692	85.1701	42.6662
2022/4/5	2:00:00	5.4181	83.1179	43.2611
2022/4/5	3:00:00	4.9918	80.2005	42.8380
2022/4/5	4:00:00	4.8644	78.3741	42.8069
2022/4/5	5:00:00	5.5981	78.0356	44.0002
2022/4/5	6:00:00	5.0434	81.9093	42.7618
2022/4/5	7:00:00	5.7761	86.1944	43.5817
2022/4/5	8:00:00	7.4419	86.9851	46.2977
2022/4/5	9:00:00	11.5294	69.0781	53.6453
2022/4/5	10:00:00	10.6064	76.4199	51.9875
2022/4/5	11:00:00	9.5147	85.6598	49.8263
2022/4/5	12:00:00	13.3786	71.7080	56.3800
2022/4/5	13:00:00	13.0738	72.7084	55.9031
2022/4/5	14:00:00	12.4169	75.1751	54.8487
2022/4/5	15:00:00	12.8502	73.6678	55.5459
2022/4/5	16:00:00	12.7158	75.7198	55.3039
2022/4/5	17:00:00	9.9936	91.3529	50.3696
2022/4/5	18:00:00	10.2114	94.0637	50.6292
2022/4/5	19:00:00	11.0160	91.6123	52.1136
2022/4/5	20:00:00	9.4599	93.5490	49.3461
2022/4/5	21:00:00	8.0052	95.0415	46.7255
2022/4/5	22:00:00	6.4947	96.7216	43.9486
2022/4/5	23:00:00	6.2270	92.9640	43.7811
2022/4/6	0:00:00	6.2550	91.4568	43.9516
2022/4/6	1:00:00	6.1400	87.4229	44.0860
2022/4/6	2:00:00	5.9948	85.4237	44.0100
2022/4/6	3:00:00	5.8556	85.2077	43.7979
2022/4/6	4:00:00	6.7108	81.0201	45.5327
2022/4/6	5:00:00	7.1694	72.4527	46.8889
2022/4/6	6:00:00	7.4253	72.6880	47.2634
2022/4/6	7:00:00	8.2289	75.0728	48.3458

2022/6/4	12:00:00	18.2875	94.6809	64.7151
2022/6/4	13:00:00	18.0268	95.7608	64.2978
2022/6/4	14:00:00	17.7666	95.8763	63.8442
2022/6/4	15:00:00	18.7089	91.4766	65.3162
2022/6/4	16:00:00	18.6955	91.2660	65.2843
2022/6/4	17:00:00	17.6736	88.1126	63.4324
2022/6/4	18:00:00	17.6408	89.4573	63.4198
2022/6/4	19:00:00	17.9456	90.0843	63.9584
2022/6/4	20:00:00	18.3482	88.8263	64.5949
2022/6/4	21:00:00	18.0241	91.3625	64.1372
2022/6/4	22:00:00	16.9914	93.5276	62.4214
2022/6/4	23:00:00	17.2075	91.4694	62.7402
2022/6/5	0:00:00	16.9484	92.2548	62.3152
2022/6/5	1:00:00	17.2204	89.9638	62.7209
2022/6/5	2:00:00	17.7279	85.9396	63.4531
2022/6/5	3:00:00	17.5833	85.8215	63.2093
2022/6/5	4:00:00	16.6216	89.5886	61.6945
2022/6/5	5:00:00	17.8628	85.0208	63.6461
2022/6/5	6:00:00	17.6365	86.3787	63.3152
2022/6/5	7:00:00	17.7741	84.1743	63.4717
2022/6/5	8:00:00	17.2237	84.5968	62.5788
2022/6/5	9:00:00	20.3334	71.8075	66.9564
2022/6/5	10:00:00	19.3142	75.9541	65.6062
2022/6/5	11:00:00	18.4810	77.0588	64.3490
2022/6/5	12:00:00	20.3559	71.1698	66.9534
2022/6/5	13:00:00	20.4430	68.9584	66.9540
2022/6/5	14:00:00	19.8958	70.6543	66.2287
2022/6/5	15:00:00	21.1117	66.6699	67.8011
2022/6/5	16:00:00	20.8424	67.1219	67.4338
2022/6/5	17:00:00	20.0522	71.9782	66.5383
2022/6/5	18:00:00	20.1753	72.3042	66.7442
2022/6/5	19:00:00	20.4591	71.0941	67.1052
2022/6/5	20:00:00	20.4194	73.5920	67.1928
2022/6/5	21:00:00	19.9399	74.9235	66.5275
2022/6/5	22:00:00	18.6482	78.6466	64.6780
2022/6/5	23:00:00	18.6691	76.7528	64.6321
2022/6/6	0:00:00	18.0375	78.0473	63.6866
2022/6/6	1:00:00	18.0595	76.2203	63.6561
2022/6/6	2:00:00	18.1278	74.5545	63.7022
2022/6/6	3:00:00	17.3968	74.5698	62.5709
2022/6/6	4:00:00	17.5134	73.2579	62.7116
2022/6/6	5:00:00	18.2444	70.6864	63.7372
2022/6/6	6:00:00	18.7164	74.8243	64.6248
2022/6/6	7:00:00	19.8673	70.2882	66.1660

2022/7/4	12:00:00	25.4438	71.0197	74.6431
2022/7/4	13:00:00	25.1240	71.1700	74.1751
2022/7/4	14:00:00	25.0073	72.7971	74.1685
2022/7/4	15:00:00	25.2336	70.4187	74.2609
2022/7/4	16:00:00	25.9126	70.3566	75.2771
2022/7/4	17:00:00	22.5690	82.4045	71.2090
2022/7/4	18:00:00	22.9937	80.8807	71.7704
2022/7/4	19:00:00	23.1592	81.6128	72.1002
2022/7/4	20:00:00	23.1033	80.7831	71.9387
2022/7/4	21:00:00	22.1266	80.2286	70.3242
2022/7/4	22:00:00	20.2490	85.9788	67.6424
2022/7/4	23:00:00	21.0956	80.5003	68.6881
2022/7/5	0:00:00	21.3455	79.3536	69.0114
2022/7/5	1:00:00	21.5229	77.3608	69.1548
2022/7/5	2:00:00	20.9892	79.9761	68.4831
2022/7/5	3:00:00	20.4651	79.6789	67.6259
2022/7/5	4:00:00	20.9725	76.5584	68.2355
2022/7/5	5:00:00	21.5923	65.0514	68.3930
2022/7/5	6:00:00	21.0053	70.5256	67.8951
2022/7/5	7:00:00	21.7493	75.2726	69.3604
2022/7/5	8:00:00	21.7820	75.1010	69.3989
2022/7/5	9:00:00	25.7588	63.0713	74.2294
2022/7/5	10:00:00	25.5293	64.4922	74.0561
2022/7/5	11:00:00	24.9020	68.7411	73.5873
2022/7/5	12:00:00	25.5309	68.8575	74.5376
2022/7/5	13:00:00	25.8120	69.9816	75.0834
2022/7/5	14:00:00	26.8952	63.7944	75.9486
2022/7/5	15:00:00	26.8124	63.6281	75.8089
2022/7/5	16:00:00	27.7752	59.5467	76.6565
2022/7/5	17:00:00	24.8606	69.8207	73.6370
2022/7/5	18:00:00	25.3460	67.4684	74.1118
2022/7/5	19:00:00	26.5028	65.7522	75.6166
2022/7/5	20:00:00	25.4997	72.7366	74.9156
2022/7/5	21:00:00	24.4746	74.5743	73.5296
2022/7/5	22:00:00	20.6338	82.6305	68.0766
2022/7/5	23:00:00	20.4817	81.2599	67.7470
2022/7/6	0:00:00	19.5421	85.7837	66.4583
2022/7/6	1:00:00	18.9739	89.3102	65.6737
2022/7/6	2:00:00	18.6809	87.7159	65.1105
2022/7/6	3:00:00	17.9144	86.3703	63.7777
2022/7/6	4:00:00	17.5107	85.0807	63.0664
2022/7/6	5:00:00	20.3382	64.5830	66.5422
2022/7/6	6:00:00	21.1193	64.7295	67.6840
2022/7/6	7:00:00	21.9847	70.7711	69.3906

2022/8/4	12:00:00	22.0055	84.1192	70.4211
2022/8/4	13:00:00	22.2495	85.4602	70.9257
2022/8/4	14:00:00	22.5651	83.2789	71.2729
2022/8/4	15:00:00	23.3446	78.8771	72.1591
2022/8/4	16:00:00	24.0671	75.5473	72.9913
2022/8/4	17:00:00	20.3944	87.8355	67.9933
2022/8/4	18:00:00	21.3749	86.5091	69.5492
2022/8/4	19:00:00	21.9249	84.7879	70.3382
2022/8/4	20:00:00	22.5167	79.3036	70.8761
2022/8/4	21:00:00	21.7496	82.1379	69.8575
2022/8/4	22:00:00	19.8907	83.9131	66.9359
2022/8/4	23:00:00	20.7669	79.7700	68.1142
2022/8/5	0:00:00	19.2902	87.2343	66.1099
2022/8/5	1:00:00	18.9940	86.3763	65.5756
2022/8/5	2:00:00	19.9154	80.3644	66.7842
2022/8/5	3:00:00	18.3312	87.8430	64.5283
2022/8/5	4:00:00	18.2683	85.5744	64.3368
2022/8/5	5:00:00	18.9763	82.0238	65.3508
2022/8/5	6:00:00	18.5387	83.5605	64.7032
2022/8/5	7:00:00	19.0644	84.4510	65.6048
2022/8/5	8:00:00	19.7998	82.8200	66.7288
2022/8/5	9:00:00	22.9478	72.6742	71.0057
2022/8/5	10:00:00	22.0205	74.4139	69.7179
2022/8/5	11:00:00	22.2522	76.0602	70.2035
2022/8/5	12:00:00	23.7505	72.1716	72.1870
2022/8/5	13:00:00	23.6870	72.0000	72.0746
2022/8/5	14:00:00	23.6343	70.8392	71.8888
2022/8/5	15:00:00	25.2841	68.0197	74.0796
2022/8/5	16:00:00	25.3643	69.2782	74.3344
2022/8/5	17:00:00	23.5150	76.9463	72.2568
2022/8/5	18:00:00	23.7972	76.8491	72.6914
2022/8/5	19:00:00	23.1742	81.9314	72.1519
2022/8/5	20:00:00	24.2466	77.5983	73.4700
2022/8/5	21:00:00	24.5380	72.5539	73.4258
2022/8/5	22:00:00	21.1728	85.0906	69.1179
2022/8/5	23:00:00	19.9557	89.1965	67.3308
2022/8/6	0:00:00	19.7348	89.2450	66.9593
2022/8/6	1:00:00	19.9052	85.5681	67.0491
2022/8/6	2:00:00	19.2510	86.5618	66.0123
2022/8/6	3:00:00	18.8601	85.4223	65.3110
2022/8/6	4:00:00	18.7478	83.5705	65.0461
2022/8/6	5:00:00	18.7295	82.7125	64.9798
2022/8/6	6:00:00	18.6516	82.4766	64.8430
2022/8/6	7:00:00	19.9047	82.4460	66.8795

2022/4/6	8:00:00	9.0927	74.6097	49.7121
2022/4/6	9:00:00	12.4362	61.3999	55.1527
2022/4/6	10:00:00	12.1847	68.3102	54.6414
2022/4/6	11:00:00	11.8175	75.7627	53.9019
2022/4/6	12:00:00	14.7870	64.9799	58.4978
2022/4/6	13:00:00	14.8063	68.4018	58.5381
2022/4/6	14:00:00	14.7128	68.9578	58.4005
2022/4/6	15:00:00	15.2498	67.7338	59.1924
2022/4/6	16:00:00	14.6225	73.3830	58.2735
2022/4/6	17:00:00	12.2567	77.6853	54.5454
2022/4/6	18:00:00	12.5056	77.6760	54.9386
2022/4/6	19:00:00	13.4275	72.2005	56.4494
2022/4/6	20:00:00	11.6541	74.7359	53.6753
2022/4/6	21:00:00	10.6833	76.5567	52.1029
2022/4/6	22:00:00	8.2837	80.0562	48.1271
2022/4/6	23:00:00	8.0778	79.5733	47.8276
2022/4/7	0:00:00	7.1930	79.4612	46.4219
2022/4/7	1:00:00	6.9506	76.5883	46.2480
2022/4/7	2:00:00	6.9463	74.0243	46.4315
2022/4/7	3:00:00	6.2642	71.5444	45.5799
2022/4/7	4:00:00	6.3571	67.9973	46.0051
2022/4/7	5:00:00	6.7302	62.1264	47.0068
2022/4/7	6:00:00	6.9565	60.9483	47.4166
2022/4/7	7:00:00	7.9612	68.2207	48.3699
2022/4/7	8:00:00	10.9763	72.7556	52.6927
2022/4/7	9:00:00	15.3476	53.7273	59.2120
2022/4/7	10:00:00	14.5268	64.1028	58.1190
2022/4/7	11:00:00	13.8371	71.6605	57.0772
2022/4/7	12:00:00	16.8447	64.2801	61.4717
2022/4/7	13:00:00	16.1201	66.2929	60.4570
2022/4/7	14:00:00	15.9970	66.3551	60.2774
2022/4/7	15:00:00	17.0876	62.1450	61.7672
2022/4/7	16:00:00	17.2027	63.1854	61.9596
2022/4/7	17:00:00	13.4420	79.9222	56.3949
2022/4/7	18:00:00	13.6345	82.1251	56.6854
2022/4/7	19:00:00	14.5026	78.9811	58.0926
2022/4/7	20:00:00	15.8862	73.2274	60.2131
2022/4/7	21:00:00	15.1406	74.6548	59.0785
2022/4/7	22:00:00	14.1107	82.7268	57.4564
2022/4/7	23:00:00	14.1209	82.8939	57.4725
2022/4/8	0:00:00	13.6904	82.9172	56.7702
2022/4/8	1:00:00	13.8710	80.6662	57.0775
2022/4/8	2:00:00	13.5264	79.2896	56.5357
2022/4/8	3:00:00	12.4895	80.4594	54.8592

2022/6/6	8:00:00	19.7937	63.2504	65.6824
2022/6/6	9:00:00	22.3207	55.7336	68.7255
2022/6/6	10:00:00	21.7729	62.2138	68.4498
2022/6/6	11:00:00	21.1649	67.0497	67.9046
2022/6/6	12:00:00	23.1829	60.8501	70.3423
2022/6/6	13:00:00	22.6179	64.7591	69.8607
2022/6/6	14:00:00	22.6636	65.9738	70.0258
2022/6/6	15:00:00	22.4981	68.5631	69.9900
2022/6/6	16:00:00	22.3857	71.6505	70.0655
2022/6/6	17:00:00	20.1839	70.4288	66.6508
2022/6/6	18:00:00	20.5199	68.4505	67.0382
2022/6/6	19:00:00	20.9698	65.1998	67.4975
2022/6/6	20:00:00	20.6532	66.9464	67.1441
2022/6/6	21:00:00	19.6190	71.9580	65.8776
2022/6/6	22:00:00	18.2595	74.5870	63.9073
2022/6/6	23:00:00	18.1939	72.9322	63.7443
2022/6/7	0:00:00	17.0592	75.5367	62.0733
2022/6/7	1:00:00	16.3716	77.7366	61.0442
2022/6/7	2:00:00	16.0120	76.4067	60.4555
2022/6/7	3:00:00	15.1863	78.2823	59.1759
2022/6/7	4:00:00	15.2648	76.6270	59.2869
2022/6/7	5:00:00	15.2734	79.8958	59.3272
2022/6/7	6:00:00	15.3519	81.3207	59.4656
2022/6/7	7:00:00	17.6128	81.2944	63.1164
2022/6/7	8:00:00	19.0239	66.8631	64.7407
2022/6/7	9:00:00	22.5986	50.1221	68.6510
2022/6/7	10:00:00	22.7421	50.1319	68.8393
2022/6/7	11:00:00	22.3088	51.6522	68.3917
2022/6/7	12:00:00	23.5307	46.8069	69.5703
2022/6/7	13:00:00	23.3011	49.7245	69.5338
2022/6/7	14:00:00	23.3103	51.0290	69.6603
2022/6/7	15:00:00	22.9819	52.9953	69.3945
2022/6/7	16:00:00	23.0501	52.2412	69.4214
2022/6/7	17:00:00	21.3827	53.2771	67.2795
2022/6/7	18:00:00	20.6371	60.7765	66.7421
2022/6/7	19:00:00	20.6371	57.6216	66.5486
2022/6/7	20:00:00	20.4118	59.5636	66.3524
2022/6/7	21:00:00	19.8985	62.6986	65.8032
2022/6/7	22:00:00	18.3052	67.2887	63.6991
2022/6/7	23:00:00	17.9886	67.7989	63.2496
2022/6/8	0:00:00	17.7461	67.6145	62.8845
2022/6/8	1:00:00	18.1085	65.0004	63.3257
2022/6/8	2:00:00	17.5607	67.6607	62.6116
2022/6/8	3:00:00	17.0651	70.1391	61.9424

2022/7/6	8:00:00	23.4893	63.8427	71.0430
2022/7/6	9:00:00	27.6542	50.3073	75.2789
2022/7/6	10:00:00	26.8952	55.1786	74.8866
2022/7/6	11:00:00	26.2249	59.5786	74.4906
2022/7/6	12:00:00	28.9352	51.5456	77.1321
2022/7/6	13:00:00	28.1746	55.1467	76.6174
2022/7/6	14:00:00	28.0966	56.7571	76.7293
2022/7/6	15:00:00	29.2846	52.7383	77.7687
2022/7/6	16:00:00	29.3211	53.1858	77.8833
2022/7/6	17:00:00	26.6157	65.3578	75.7340
2022/7/6	18:00:00	26.7291	62.2789	75.5248
2022/7/6	19:00:00	27.7918	59.2743	76.6438
2022/7/6	20:00:00	26.7458	66.6079	76.0758
2022/7/6	21:00:00	25.7916	71.9174	75.2702
2022/7/6	22:00:00	22.5851	78.1162	70.8896
2022/7/6	23:00:00	22.5927	77.0603	70.8163
2022/7/7	0:00:00	21.9460	77.8670	69.8591
2022/7/7	1:00:00	22.1815	75.9863	70.0873
2022/7/7	2:00:00	21.9240	76.8604	69.7497
2022/7/7	3:00:00	20.8112	78.9093	68.1309
2022/7/7	4:00:00	20.6070	79.4041	67.8360
2022/7/7	5:00:00	21.9772	72.1672	69.4833
2022/7/7	6:00:00	21.9105	74.7563	69.5731
2022/7/7	7:00:00	22.0987	75.1253	69.8927
2022/7/7	8:00:00	22.1341	72.2269	69.7272
2022/7/7	9:00:00	26.3937	59.0949	74.6696
2022/7/7	10:00:00	25.7916	61.4926	74.0991
2022/7/7	11:00:00	25.0589	64.1264	73.3363
2022/7/7	12:00:00	27.5209	56.8242	75.9482
2022/7/7	13:00:00	27.6838	55.9286	76.0544
2022/7/7	14:00:00	27.7101	56.6628	76.1867
2022/7/7	15:00:00	27.8402	55.6844	76.2353
2022/7/7	16:00:00	27.9617	55.3324	76.3536
2022/7/7	17:00:00	25.1535	59.4195	72.9740
2022/7/7	18:00:00	25.7201	55.7590	73.3576
2022/7/7	19:00:00	26.6377	57.8559	74.8605
2022/7/7	20:00:00	25.8567	64.6586	74.5491
2022/7/7	21:00:00	24.7396	67.7520	73.2445
2022/7/7	22:00:00	21.9804	75.6119	69.7452
2022/7/7	23:00:00	22.7480	72.6172	70.6954
2022/7/8	0:00:00	22.3234	73.1526	70.0879
2022/7/8	1:00:00	22.1519	73.2491	69.8322
2022/7/8	2:00:00	21.6977	74.0372	69.1915
2022/7/8	3:00:00	20.4457	76.5675	67.4101

2022/8/6	8:00:00	20.5852	77.9194	67.7111
2022/8/6	9:00:00	23.9741	67.3870	72.0765
2022/8/6	10:00:00	23.6403	72.1463	72.0167
2022/8/6	11:00:00	22.4560	77.9183	70.6693
2022/8/6	12:00:00	24.5412	70.3641	73.2119
2022/8/6	13:00:00	24.4681	71.1938	73.1840
2022/8/6	14:00:00	24.1929	71.1626	72.7641
2022/8/6	15:00:00	25.4390	68.1537	74.3238
2022/8/6	16:00:00	25.6282	68.9430	74.6922
2022/8/6	17:00:00	24.2423	73.1031	73.0272
2022/8/6	18:00:00	23.4693	77.1063	72.1993
2022/8/6	19:00:00	23.2252	81.2177	72.1727
2022/8/6	20:00:00	23.7225	79.5322	72.8205
2022/8/6	21:00:00	23.2510	79.4511	72.0603
2022/8/6	22:00:00	19.4122	95.7503	66.7330
2022/8/6	23:00:00	19.8987	94.4284	67.5169
2022/8/7	0:00:00	19.4547	95.3246	66.7866
2022/8/7	1:00:00	20.0842	90.8814	67.6424
2022/8/7	2:00:00	19.8638	89.2913	67.1803
2022/8/7	3:00:00	18.6414	92.1581	65.2286
2022/8/7	4:00:00	19.2106	87.4223	65.9856
2022/8/7	5:00:00	19.1628	88.4545	65.9537
2022/8/7	6:00:00	19.6751	84.5355	66.6144
2022/8/7	7:00:00	21.3077	82.6402	69.1743
2022/8/7	8:00:00	21.4082	79.7811	69.1409
2022/8/7	9:00:00	25.5148	70.3942	74.6819
2022/8/7	10:00:00	25.4067	72.9712	74.7987
2022/8/7	11:00:00	24.8347	74.9408	74.1248
2022/8/7	12:00:00	26.8668	68.1967	76.4490
2022/8/7	13:00:00	26.4399	70.2606	76.0602
2022/8/7	14:00:00	26.3711	70.8087	76.0213
2022/8/7	15:00:00	26.3007	72.0606	76.0618
2022/8/7	16:00:00	26.1754	72.4962	75.9216
2022/8/7	17:00:00	23.7101	82.8362	73.1038
2022/8/7	18:00:00	23.6634	83.5809	73.0955
2022/8/7	19:00:00	23.7865	85.5224	73.4767
2022/8/7	20:00:00	24.2294	81.9680	73.8662
2022/8/7	21:00:00	23.5908	86.6153	73.2514
2022/8/7	22:00:00	21.5405	93.2948	70.3018
2022/8/7	23:00:00	21.5308	93.6149	70.3075
2022/8/8	0:00:00	21.2814	93.8015	69.8869
2022/8/8	1:00:00	20.6782	93.8858	68.8435
2022/8/8	2:00:00	20.5196	91.4605	68.4217
2022/8/8	3:00:00	19.0897	94.9535	66.1294

2022/4/8	4:00:00	12.5529	78.5611	54.9967	2022/6/8	4:00:00	16.6807	70.9957	61.3832	2022/7/8	4:00:00	20.1441	76.0273	66.9067	2022/8/8	4:00:00	18.8510	95.0650	65.7165
2022/4/8	5:00:00	11.1214	74.3870	52.8612	2022/6/8	5:00:00	17.7150	76.5313	63.1271	2022/7/8	5:00:00	21.6530	72.2682	68.9964	2022/8/8	5:00:00	20.3557	89.7171	68.0385
2022/4/8	6:00:00	10.7822	73.4343	52.3712	2022/6/8	6:00:00	17.6806	80.6993	63.2067	2022/7/8	6:00:00	21.1606	74.6699	68.4049	2022/8/8	6:00:00	20.2686	88.5849	67.8253
2022/4/8	7:00:00	11.1983	75.6412	52.9398	2022/6/8	7:00:00	17.5107	83.5779	63.0208	2022/7/8	7:00:00	21.4106	79.2395	69.1073	2022/8/8	7:00:00	21.0518	87.2720	69.0607
2022/4/8	8:00:00	11.1166	83.3829	52.5573	2022/6/8	8:00:00	17.5032	84.9565	63.0502	2022/7/8	8:00:00	22.1486	68.9702	69.5009	2022/8/8	8:00:00	21.2475	87.8622	69.4280
2022/4/8	9:00:00	15.2041	72.2750	59.1589	2022/6/8	9:00:00	19.2771	74.6585	65.4864	2022/7/8	9:00:00	26.6538	59.1086	75.0342	2022/8/8	9:00:00	25.8405	72.0430	75.3588
2022/4/8	10:00:00	14.7956	75.4072	58.5465	2022/6/8	10:00:00	18.9546	79.2181	65.1903	2022/7/8	10:00:00	25.8271	64.9050	74.5340	2022/8/8	10:00:00	25.3755	74.6050	74.9278
2022/4/8	11:00:00	14.6391	76.9528	58.3060	2022/6/8	11:00:00	19.2196	80.8009	65.6876	2022/7/8	11:00:00	25.4347	66.8776	74.1786	2022/8/8	11:00:00	24.6891	78.3635	74.2459
2022/4/8	12:00:00	16.7146	69.9079	61.4100	2022/6/8	12:00:00	20.3264	76.3500	67.2103	2022/7/8	12:00:00	27.9767	58.1417	76.7504	2022/8/8	12:00:00	25.9475	75.4519	75.9100
2022/4/8	13:00:00	16.3700	72.0814	60.9338	2022/6/8	13:00:00	20.4887	75.7201	67.4268	2022/7/8	13:00:00	27.7590	59.8022	76.6676	2022/8/8	13:00:00	25.7320	75.9600	75.6311
2022/4/8	14:00:00	16.2824	74.5490	60.8452	2022/6/8	14:00:00	19.8377	78.9736	66.5852	2022/7/8	14:00:00	27.6848	60.4911	76.6539	2022/8/8	14:00:00	25.7486	74.4884	75.4925
2022/4/8	15:00:00	16.9366	74.1083	61.8471	2022/6/8	15:00:00	19.5614	80.2119	66.2081	2022/7/8	15:00:00	28.5906	55.9522	77.2944	2022/8/8	15:00:00	26.6157	71.6032	76.4866
2022/4/8	16:00:00	16.7425	76.2321	61.5958	2022/6/8	16:00:00	19.5270	82.2333	66.2547	2022/7/8	16:00:00	28.0789	56.3649	76.6521	2022/8/8	16:00:00	26.9888	70.0403	76.8592
2022/4/8	17:00:00	13.1404	88.5799	55.8002	2022/6/8	17:00:00	18.1160	79.0457	63.8471	2022/7/8	17:00:00	25.0751	67.7352	73.7395	2022/8/8	17:00:00	24.3332	80.5359	73.8942
2022/4/8	18:00:00	14.0070	88.6899	57.2616	2022/6/8	18:00:00	18.6471	82.6055	64.8410	2022/7/8	18:00:00	25.5578	62.5998	73.8891	2022/8/8	18:00:00	25.0562	77.4963	74.7370
2022/4/8	19:00:00	15.5621	82.3236	59.8162	2022/6/8	19:00:00	18.7180	80.2825	64.8583	2022/7/8	19:00:00	25.5293	69.0002	74.5508	2022/8/8	19:00:00	25.4680	78.9972	75.5503
2022/4/8	20:00:00	16.4781	78.0603	61.2188	2022/6/8	20:00:00	19.5673	75.3183	65.9694	2022/7/8	20:00:00	25.5368	69.2393	74.5883	2022/8/8	20:00:00	24.6299	80.9947	74.4174
2022/4/8	21:00:00	16.1985	78.9325	60.7915	2022/6/8	21:00:00	18.6218	80.7137	64.7217	2022/7/8	21:00:00	24.6569	72.8463	73.6370	2022/8/8	21:00:00	23.8881	85.2093	73.6157
2022/4/8	22:00:00	14.1091	80.5464	57.4610	2022/6/8	22:00:00	16.8022	86.7345	61.9343	2022/7/8	22:00:00	21.8852	79.7567	69.9022	2022/8/8	22:00:00	21.1663	94.4185	69.7280
2022/4/8	23:00:00	14.4999	77.4308	58.0874	2022/6/8	23:00:00	17.0979	85.2312	62.3882	2022/7/8	23:00:00	22.2793	77.6463	70.3689	2022/8/8	23:00:00	21.5373	92.8794	70.2671
2022/4/9	0:00:00	14.4779	76.0717	58.0522	2022/6/9	0:00:00	18.0004	78.8690	63.6568	2022/7/9	0:00:00	21.0305	80.3309	68.5725	2022/8/9	0:00:00	21.4593	92.1488	70.0815
2022/4/9	1:00:00	14.6907	74.6913	58.3816	2022/6/9	1:00:00	18.1187	77.8150	63.8066	2022/7/9	1:00:00	21.1811	78.0154	68.6597	2022/8/9	1:00:00	20.7857	92.8456	68.9652
2022/4/9	2:00:00	13.7796	77.0886	56.9541	2022/6/9	2:00:00	18.0595	77.0113	63.6844	2022/7/9	2:00:00	21.0542	77.7518	68.4418	2022/8/9	2:00:00	20.7105	91.8017	68.7703
2022/4/9	3:00:00	12.0766	81.2308	54.1779	2022/6/9	3:00:00	17.5521	81.2729	63.0176	2022/7/9	3:00:00	19.6157	82.1002	66.3919	2022/8/9	3:00:00	19.3740	95.3336	66.6456
2022/4/9	4:00:00	12.0530	76.9195	54.2418	2022/6/9	4:00:00	17.2650	82.6235	62.5919	2022/7/9	4:00:00	18.4175	86.5216	64.6214	2022/8/9	4:00:00	19.4940	94.6638	66.8224
2022/4/9	5:00:00	11.1848	85.5107	52.6003	2022/6/9	5:00:00	16.6157	86.6322	61.6209	2022/7/9	5:00:00	19.6620	79.7067	66.3433	2022/8/9	5:00:00	20.6551	91.2323	68.6401
2022/4/9	6:00:00	10.8371	85.5075	52.0243	2022/6/9	6:00:00	16.0373	90.4206	60.7161	2022/7/9	6:00:00	19.1750	81.7822	65.6617	2022/8/9	6:00:00	21.2050	88.7813	69.4182
2022/4/9	7:00:00	11.1359	88.1864	52.4316	2022/6/9	7:00:00	15.7502	92.0402	60.2475	2022/7/9	7:00:00	20.9800	79.7617	68.4546	2022/8/9	7:00:00	21.8211	90.6780	70.5972
2022/4/9	8:00:00	13.3264	84.2668	56.1617	2022/6/9	8:00:00	15.4750	92.1179	59.7746	2022/7/9	8:00:00	21.9089	68.6348	69.1182	2022/8/9	8:00:00	21.8302	81.9105	69.9717
2022/4/9	9:00:00	17.6306	66.8751	62.6902	2022/6/9	9:00:00	16.4662	90.0273	61.4396	2022/7/9	9:00:00	26.2265	56.9910	74.1910	2022/8/9	9:00:00	25.9163	72.3613	75.5104
2022/4/9	10:00:00	17.4650	68.3196	62.4897	2022/6/9	10:00:00	17.1527	91.2370	62.6399	2022/7/9	10:00:00	26.0163	60.5537	74.3103	2022/8/9	10:00:00	25.2621	76.3528	74.9393
2022/4/9	11:00:00	17.3876	71.5702	62.4693	2022/6/9	11:00:00	16.6334	92.7219	61.7824	2022/7/9	11:00:00	25.5976	63.5102	74.0465	2022/8/9	11:00:00	24.7810	79.9300	74.5519
2022/4/9	12:00:00	20.2979	61.8810	66.3272	2022/6/9	12:00:00	17.7902	86.6745	63.5810	2022/7/9	12:00:00	27.0371	60.8269	75.7832	2022/8/9	12:00:00	26.9952	73.9763	77.3579
2022/4/9	13:00:00	18.9400	64.3598	64.5058	2022/6/9	13:00:00	17.7150	87.8891	63.4948	2022/7/9	13:00:00	27.0269	61.4689	75.8487	2022/8/9	13:00:00	27.3619	71.8231	77.6480
2022/4/9	14:00:00	18.9293	63.5962	64.4564	2022/6/9	14:00:00	17.4650	90.9020	63.1650	2022/7/9	14:00:00	27.0979	63.2553	76.1732	2022/8/9	14:00:00	26.4163	70.5201	76.0553
2022/4/9	15:00:00	19.5093	62.4655	65.2347	2022/6/9	15:00:00	18.7046	86.8071	65.1118	2022/7/9	15:00:00	27.1452	63.0283	76.2126	2022/8/9	15:00:00	26.4560	69.3888	75.9808
2022/4/9	16:00:00	19.4975	62.1573	65.2024	2022/6/9	16:00:00	18.9427	84.3269	65.3989	2022/7/9	16:00:00	27.0032	62.6102	75.9571	2022/8/9	16:00:00	27.0044	68.0378	76.6336
2022/4/9	17:00:00	16.0297	70.2808	60.3871	2022/6/9	17:00:00	17.0059	90.0384	62.3581	2022/7/9	17:00:00	24.9213	67.9005	73.5290	2022/8/9	17:00:00	22.7479	77.0745	71.0616
2022/4/9	18:00:00	16.7662	67.6900	61.4365	2022/6/9	18:00:00	17.2323	88.5111	62.7010	2022/7/9	18:00:00	24.8283	67.5254	73.3526	2022/8/9	18:00:00	23.1349	74.1914	71.4224
2022/4/9	19:00:00	17.4575	65.4473	62.3928	2022/6/9	19:00:00	17.4398	89.1980	63.0712	2022/7/9	19:00:00	24.4703	75.7070	73.6354	2022/8/9	19:00:00	23.0312	78.2641	71.6084
2022/4/9	20:00:00	17.3258	64.6551	62.1782	2022/6/9	20:00:00	18.2165	84.1681	64.1985	2022/7/9	20:00:00	24.5160	76.3380	73.7695	2022/8/9	20:00:00	22.2442	85.0771	70.8872
2022/4/9	21:00:00	14.3402	75.8354	57.8374	2022/6/9	21:00:00	18.6186	83.2672	64.8220	2022/7/9	21:00:00	24.0355	76.7090	73.0523	2022/8/9	21:00:00	21.9792	89.6369	70.7894
2022/4/9	22:00:00	11.6407	80.2403	53.5017	2022/6/9	22:00:00	17.2145	88.4522	62.6694	2022/7/9	22:00:00	21.3455	82.1844	69.2048	2022/8/9	22:00:00	19.7219	92.8955	67.1282
2022/4/9	23:00:00	11.6939	79.0798	53.6187	2022/6/9	23:00:00	17.4145	86.9000	62.9609	2022/7/9	23:00:00	21.6192	78.8585	69.4128	2022/8/9	23:00:00	20.6218	90.5332	68.5402

2022/4/10	0:00:00	11.5058	78.7748	53.3279	2022/6/10	0:00:00	17.3596	86.0327	62.8443	2022/7/10	0:00:00	21.2967	78.2252	68.8568	2022/8/10	0:00:00	20.4944	88.2149	68.1840
2022/4/10	1:00:00	12.0718	73.9718	54.3406	2022/6/10	1:00:00	17.0726	85.6877	62.3583	2022/7/10	1:00:00	21.1074	76.2879	68.4292	2022/8/10	1:00:00	21.0825	84.4808	68.9286
2022/4/10	2:00:00	12.1594	71.5896	54.5296	2022/6/10	2:00:00	17.3097	82.8433	62.6707	2022/7/10	2:00:00	21.2284	75.5525	68.5692	2022/8/10	2:00:00	20.7406	82.6039	68.2487
2022/4/10	3:00:00	10.9542	74.1430	52.6111	2022/6/10	3:00:00	16.9484	82.4811	62.0729	2022/7/10	3:00:00	20.1366	78.0504	67.0090	2022/8/10	3:00:00	19.8138	84.4865	66.8402
2022/4/10	4:00:00	10.7425	73.6206	52.3032	2022/6/10	4:00:00	17.1097	81.7813	62.3167	2022/7/10	4:00:00	19.7996	78.6845	66.5092	2022/8/10	4:00:00	19.5020	84.3932	66.3222
2022/4/10	5:00:00	9.9614	69.3737	51.2898	2022/6/10	5:00:00	17.1898	83.0590	62.4812	2022/7/10	5:00:00	21.7256	79.1376	69.6023	2022/8/10	5:00:00	20.8476	85.5168	68.6075
2022/4/10	6:00:00	10.0662	67.8176	51.5141	2022/6/10	6:00:00	16.9441	87.2913	62.1849	2022/7/10	6:00:00	21.6767	79.0886	69.5208	2022/8/10	6:00:00	20.0261	89.6193	67.4734
2022/4/10	7:00:00	12.0868	66.8039	54.5311	2022/6/10	7:00:00	16.9307	88.4768	62.1916	2022/7/10	7:00:00	22.5185	81.4378	71.0495	2022/8/10	7:00:00	20.6186	92.0693	68.6286
2022/4/10	8:00:00	13.5694	71.5547	56.6713	2022/6/10	8:00:00	16.2948	89.0287	61.1296	2022/7/10	8:00:00	22.9464	72.0090	70.9475	2022/8/10	8:00:00	20.4186	89.0667	68.1068
2022/4/10	9:00:00	18.9562	55.9020	64.1514	2022/6/10	9:00:00	18.1321	82.9665	64.0159	2022/7/10	9:00:00	25.3062	66.2282	73.9196	2022/8/10	9:00:00	24.4434	76.3592	73.6579
2022/4/10	10:00:00	18.5224	63.5422	63.8684	2022/6/10	10:00:00	17.6069	86.7802	63.2786	2022/7/10	10:00:00	24.6493	67.8440	73.1201	2022/8/10	10:00:00	24.0182	80.3172	73.3671
2022/4/10	11:00:00	18.2595	65.2671	63.5553	2022/6/10	11:00:00	17.2887	89.8651	62.8343	2022/7/10	11:00:00	24.2747	69.4939	72.7255	2022/8/10	11:00:00	24.4934	78.7995	73.9789
2022/4/10	12:00:00	21.0865	60.6153	67.3658	2022/6/10	12:00:00	18.3719	85.0990	64.4900	2022/7/10	12:00:00	26.5490	63.5543	75.4207	2022/8/10	12:00:00	26.3915	72.7742	76.2846
2022/4/10	13:00:00	20.4210	66.4804	66.7745	2022/6/10	13:00:00	18.2106	86.4348	64.2733	2022/7/10	13:00:00	26.1969	64.8856	75.0689	2022/8/10	13:00:00	25.7921	75.1222	75.6310
2022/4/10	14:00:00	19.4695	71.5111	65.6278	2022/6/10	14:00:00	18.3955	85.1451	64.5308	2022/7/10	14:00:00	26.0400	65.3886	74.8987	2022/8/10	14:00:00	25.5803	74.5937	75.2437
2022/4/10	15:00:00	20.4398	68.1056	66.8986	2022/6/10	15:00:00	18.2224	86.9728	64.3131	2022/7/10	15:00:00	26.2958	66.8393	75.4418	2022/8/10	15:00:00	26.4104	69.2154	75.8918
2022/4/10	16:00:00	19.9017	71.9065	66.3053	2022/6/10	16:00:00	18.2057	86.1655	64.2552	2022/7/10	16:00:00	26.1507	67.3332	75.2854	2022/8/10	16:00:00	26.2405	68.5142	75.5560
2022/4/10	17:00:00	16.2824	82.2822	60.9859	2022/6/10	17:00:00	17.2382	89.1416	62.7284	2022/7/10	17:00:00	24.1311	75.5964	73.0958	2022/8/10	17:00:00	24.2165	69.3398	72.6236
2022/4/10	18:00:00	17.4414	79.2379	62.7785	2022/6/10	18:00:00	17.4069	88.6194	62.9987	2022/7/10	18:00:00	24.5144	74.5434	73.5881	2022/8/10	18:00:00	24.8863	66.9319	73.3770
2022/4/10	19:00:00	18.6852	75.7438	64.6151	2022/6/10	19:00:00	17.6720	88.2666	63.4346	2022/7/10	19:00:00	24.8961	78.3970	74.5776	2022/8/10	19:00:00	24.6955	69.1739	73.3235
2022/4/10	20:00:00	19.6991	71.0612	65.9529	2022/6/10	20:00:00	18.3364	84.3365	64.4019	2022/7/10	20:00:00	25.0030	77.2715	74.6296	2022/8/10	20:00:00	24.0773	75.1603	72.9703
2022/4/10	21:00:00	18.8438	74.5017	64.8083	2022/6/10	21:00:00	18.1160	84.1774	64.0337	2022/7/10	21:00:00	23.8785	81.3464	73.2391	2022/8/10	21:00:00	23.0699	78.8055	71.7159
2022/4/10	22:00:00	16.6834	82.9366	61.6519	2022/6/10	22:00:00	16.9425	85.4935	62.1377	2022/7/10	22:00:00	22.8679	80.6920	71.5521	2022/8/10	22:00:00	19.7471	87.4010	66.8834
2022/4/10	23:00:00	16.5431	82.3842	61.4116	2022/6/10	23:00:00	17.0269	82.5064	62.2012	2022/7/10	23:00:00	23.1431	81.0028	72.0216	2022/8/10	23:00:00	19.7622	86.2026	66.8456
2022/4/11	0:00:00	16.0045	83.6115	60.5550	2022/6/11	0:00:00	16.4071	84.9484	61.2404	2022/7/11	0:00:00	23.1625	82.5700	72.1881	2022/8/11	0:00:00	19.1596	86.2484	65.8453
2022/4/11	1:00:00	15.5949	84.1872	59.8907	2022/6/11	1:00:00	16.3431	84.1640	61.1200	2022/7/11	1:00:00	23.3576	81.9086	72.4473	2022/8/11	1:00:00	19.3252	83.5617	65.9910
2022/4/11	2:00:00	15.1896	84.3889	59.2261	2022/6/11	2:00:00	16.7915	79.7818	61.7549	2022/7/11	2:00:00	23.2716	84.0329	72.4935	2022/8/11	2:00:00	19.3273	81.8259	65.9106
2022/4/11	3:00:00	14.2752	85.8011	57.7192	2022/6/11	3:00:00	16.0787	82.2332	60.6542	2022/7/11	3:00:00	23.1076	80.9927	71.9636	2022/8/11	3:00:00	18.3984	82.8936	64.4475
2022/4/11	4:00:00	14.1344	85.2337	57.4872	2022/6/11	4:00:00	16.7119	77.3721	61.5735	2022/7/11	4:00:00	23.1356	80.0254	71.9254	2022/8/11	4:00:00	18.7929	80.6973	64.9963
2022/4/11	5:00:00	15.3788	76.0958	59.4607	2022/6/11	5:00:00	17.3983	70.0314	62.4406	2022/7/11	5:00:00	22.1562	85.2978	70.7587	2022/8/11	5:00:00	19.2278	80.4701	65.6853
2022/4/11	6:00:00	14.6273	78.0464	58.2894	2022/6/11	6:00:00	17.0651	71.0974	61.9673	2022/7/11	6:00:00	22.5615	83.8715	71.3146	2022/8/11	6:00:00	19.4724	79.9156	66.0506
2022/4/11	7:00:00	13.7839	85.3029	56.9071	2022/6/11	7:00:00	16.3964	82.6206	61.1776	2022/7/11	7:00:00	22.3572	83.2207	70.9286	2022/8/11	7:00:00	20.4481	79.0003	67.5585
2022/4/11	8:00:00	13.7393	87.1772	56.8202	2022/6/11	8:00:00	15.8701	85.6926	60.3643	2022/7/11	8:00:00	21.5719	85.6972	69.8201	2022/8/11	8:00:00	21.4373	71.6487	68.6244
2022/4/11	9:00:00	18.0020	72.0290	63.4185	2022/6/11	9:00:00	17.7413	82.4548	63.3617	2022/7/11	9:00:00	24.5945	75.0113	73.7591	2022/8/11	9:00:00	25.9819	60.8917	74.3005
2022/4/11	10:00:00	17.5967	76.2772	62.9337	2022/6/11	10:00:00	17.8773	83.0454	63.6030	2022/7/11	10:00:00	24.3650	76.8942	73.5876	2022/8/11	10:00:00	25.4976	64.5642	74.0180
2022/4/11	11:00:00	16.7517	78.5468	61.6630	2022/6/11	11:00:00	17.6085	85.5255	63.2420	2022/7/11	11:00:00	24.2747	76.6862	73.4255	2022/8/11	11:00:00	24.9213	67.4016	73.4772
2022/4/11	12:00:00	18.6041	75.9953	64.4988	2022/6/11	12:00:00	19.3099	80.4738	65.8172	2022/7/11	12:00:00	25.8223	72.7499	75.4106	2022/8/11	12:00:00	26.6754	61.0776	75.3027
2022/4/11	13:00:00	18.2251	77.5978	63.9667	2022/6/11	13:00:00	19.3260	79.4487	65.7936	2022/7/11	13:00:00	26.5738	69.6829	76.1923	2022/8/11	13:00:00	26.5453	63.0189	75.3512
2022/4/11	14:00:00	19.5077	72.5805	65.7394	2022/6/11	14:00:00	19.4486	78.2122	65.9280	2022/7/11	14:00:00	26.1421	70.4893	75.6382	2022/8/11	14:00:00	26.6453	63.0106	75.4936
2022/4/11	15:00:00	19.7474	68.9125	65.9133	2022/6/11	15:00:00	18.1692	81.5403	64.0238	2022/7/11	15:00:00	26.4055	70.7899	76.0710	2022/8/11	15:00:00	26.7754	61.9855	75.5550
2022/4/11	16:00:00	19.8807	68.7021	66.1009	2022/6/11	16:00:00	17.6569	82.7750	63.2346	2022/7/11	16:00:00	25.9083	76.1320	75.9260	2022/8/11	16:00:00	26.9711	61.6554	75.7927
2022/4/11	17:00:00	17.8139	74.2616	63.2064	2022/6/11	17:00:00	16.5635	88.7650	61.5787	2022/7/11	17:00:00	23.5113	85.3394	73.0045	2022/8/11	17:00:00	24.0397	69.6584	72.3892
2022/4/11	18:00:00	17.7564	77.9959	63.2400	2022/6/11	18:00:00	16.2991	89.2971	61.1418	2022/7/11	18:00:00	24.4198	83.1510	74.2917	2022/8/11	18:00:00	24.3090	76.6962	73.4803
2022/4/11	19:00:00	17.6069	80.1850	63.0721	2022/6/11	19:00:00	16.9543	88.1238	62.2227	2022/7/11	19:00:00	24.6907	83.6927	74.7891	2022/8/11	19:00:00	23.7961	77.2336	72.7253

2022/4/11	20:00:00	18.1085	79.2510	63.8426	2022/6/11	20:00:00	17.4112	87.4410	62.9714	2022/7/11	20:00:00	25.0278	81.9039	75.1540	2022/8/11	20:00:00	23.4499	79.6294	72.3938
2022/4/11	21:00:00	17.0844	83.4879	62.3204	2022/6/11	21:00:00	16.7904	88.8483	61.9637	2022/7/11	21:00:00	24.6225	81.6127	74.4677	2022/8/11	21:00:00	22.9521	83.1164	71.8918
2022/4/11	22:00:00	14.4612	93.6825	58.0291	2022/6/11	22:00:00	15.8018	92.4618	60.3420	2022/7/11	22:00:00	23.1270	82.7139	72.1427	2022/8/11	22:00:00	20.5503	85.3109	68.1026
2022/4/11	23:00:00	13.7484	96.3740	56.7721	2022/6/11	23:00:00	15.6793	94.0388	60.1498	2022/7/11	23:00:00	23.9274	77.3481	72.9427	2022/8/11	23:00:00	20.4745	84.3302	67.9186
2022/4/12	0:00:00	12.1874	98.7876	53.9644	2022/6/12	0:00:00	15.8846	92.1068	60.4798	2022/7/12	0:00:00	23.8710	78.0917	72.9232	2022/8/12	0:00:00	19.7208	87.0623	66.8216
2022/4/12	1:00:00	12.6475	93.9372	54.8734	2022/6/12	1:00:00	15.7959	92.4187	60.3312	2022/7/12	1:00:00	23.7909	79.2198	72.9008	2022/8/12	1:00:00	19.4499	85.9834	66.3152
2022/4/12	2:00:00	13.5291	84.4461	56.4933	2022/6/12	2:00:00	15.9808	91.3323	60.6337	2022/7/12	2:00:00	24.1978	75.5275	73.1930	2022/8/12	2:00:00	19.4574	83.3389	66.1964
2022/4/12	3:00:00	12.7658	79.9322	55.3119	2022/6/12	3:00:00	15.8626	89.6844	60.4078	2022/7/12	3:00:00	23.5780	77.4393	72.4004	2022/8/12	3:00:00	18.4247	85.9937	64.6126
2022/4/12	4:00:00	12.3707	80.1073	54.6756	2022/6/12	4:00:00	16.0695	88.2117	60.7355	2022/7/12	4:00:00	23.3442	79.3127	72.1968	2022/8/12	4:00:00	18.3532	85.2445	64.4648
2022/4/12	5:00:00	12.0928	80.3815	54.2237	2022/6/12	5:00:00	16.6807	88.5137	61.7710	2022/7/12	5:00:00	22.8469	80.8469	71.5312	2022/8/12	5:00:00	20.2035	80.2457	67.2401
2022/4/12	6:00:00	11.4751	79.9260	53.2453	2022/6/12	6:00:00	16.7323	86.8624	61.8206	2022/7/12	6:00:00	23.2764	75.6082	71.7648	2022/8/12	6:00:00	20.4428	81.0353	67.6708
2022/4/12	7:00:00	11.5343	84.3346	53.2130	2022/6/12	7:00:00	16.3270	90.5066	61.2117	2022/7/12	7:00:00	22.6916	81.0619	71.2986	2022/8/12	7:00:00	22.0141	78.1609	69.9888
2022/4/12	8:00:00	12.1653	83.8248	54.2626	2022/6/12	8:00:00	16.0787	93.2759	60.8328	2022/7/12	8:00:00	22.3325	82.4798	70.8303	2022/8/12	8:00:00	22.5608	74.4683	70.5579
2022/4/12	9:00:00	15.7099	70.2919	59.9057	2022/6/12	9:00:00	19.1841	86.1212	65.8801	2022/7/12	9:00:00	23.8016	79.1418	72.9107	2022/8/12	9:00:00	26.6711	57.7732	74.8966
2022/4/12	10:00:00	15.4083	75.8140	59.5042	2022/6/12	10:00:00	18.1455	88.9863	64.2584	2022/7/12	10:00:00	23.6952	78.6344	72.6946	2022/8/12	10:00:00	26.7808	60.6601	75.4008
2022/4/12	11:00:00	14.7794	81.2222	58.5407	2022/6/12	11:00:00	18.3433	86.8434	64.5102	2022/7/12	11:00:00	23.3533	79.7549	72.2504	2022/8/12	11:00:00	26.2324	63.3146	74.9371
2022/4/12	12:00:00	17.6381	72.4391	62.8772	2022/6/12	12:00:00	20.9150	79.3103	68.3216	2022/7/12	12:00:00	25.8610	73.1970	75.5204	2022/8/12	12:00:00	27.7527	57.5957	76.3680
2022/4/12	13:00:00	16.9721	75.3183	61.9321	2022/6/12	13:00:00	21.1236	80.5285	68.7349	2022/7/12	13:00:00	25.3815	76.5560	75.1482	2022/8/12	13:00:00	27.7570	58.4756	76.4899
2022/4/12	14:00:00	16.7425	75.7550	61.5850	2022/6/12	14:00:00	19.8614	82.4086	66.8071	2022/7/12	14:00:00	25.1020	78.3952	74.9040	2022/8/12	14:00:00	27.2705	59.4550	75.9385
2022/4/12	15:00:00	17.5682	72.6237	62.7762	2022/6/12	15:00:00	20.3350	79.8162	67.4259	2022/7/12	15:00:00	25.0218	77.6170	74.6955	2022/8/12	15:00:00	26.7162	61.7489	75.4421
2022/4/12	16:00:00	17.6187	71.5614	62.8200	2022/6/12	16:00:00	19.8646	82.1124	66.7965	2022/7/12	16:00:00	24.9568	77.6938	74.6008	2022/8/12	16:00:00	26.9060	61.6978	75.7055
2022/4/12	17:00:00	13.7984	85.9066	56.9273	2022/6/12	17:00:00	18.6320	84.8944	64.9114	2022/7/12	17:00:00	23.3426	81.7090	72.4053	2022/8/12	17:00:00	24.5197	68.0181	72.9454
2022/4/12	18:00:00	14.4719	83.1386	58.0449	2022/6/12	18:00:00	18.2917	84.9496	64.3519	2022/7/12	18:00:00	23.6226	80.2359	72.7249	2022/8/12	18:00:00	24.5106	72.8142	73.4099
2022/4/12	19:00:00	15.3460	83.4102	59.4747	2022/6/12	19:00:00	19.1916	79.8815	65.5994	2022/7/12	19:00:00	24.3295	76.9276	73.5352	2022/8/12	19:00:00	23.9370	76.2701	72.8565
2022/4/12	20:00:00	16.1851	80.2524	60.7929	2022/6/12	20:00:00	19.8055	78.0183	66.4832	2022/7/12	20:00:00	24.4064	77.6614	73.7284	2022/8/12	20:00:00	24.0359	76.0214	72.9877
2022/4/12	21:00:00	16.6748	79.1345	61.5540	2022/6/12	21:00:00	19.3023	81.2035	65.8402	2022/7/12	21:00:00	24.7101	74.7421	73.9112	2022/8/12	21:00:00	23.5575	78.3154	72.4471
2022/4/12	22:00:00	14.6950	83.4827	58.4101	2022/6/12	22:00:00	17.3672	90.0197	62.9721	2022/7/12	22:00:00	22.1858	82.5173	70.5945	2022/8/12	22:00:00	20.7422	84.8624	68.3921
2022/4/12	23:00:00	14.6375	83.2213	58.3154	2022/6/12	23:00:00	17.2634	88.4981	62.7532	2022/7/12	23:00:00	23.1829	77.0410	71.7430	2022/8/12	23:00:00	20.6341	83.5725	68.1348
2022/4/13	0:00:00	14.5354	81.3333	58.1469	2022/6/13	0:00:00	17.3639	85.8793	62.8470	2022/7/13	0:00:00	22.9743	77.8789	71.4858	2022/8/13	0:00:00	20.2982	83.9476	67.6064
2022/4/13	1:00:00	14.7708	79.8920	58.5225	2022/6/13	1:00:00	17.6897	83.6289	63.3155	2022/7/13	1:00:00	22.6873	78.9309	71.1178	2022/8/13	1:00:00	19.8498	85.3156	66.9439
2022/4/13	2:00:00	14.8773	77.9099	58.6844	2022/6/13	2:00:00	17.8821	80.8774	63.5370	2022/7/13	2:00:00	22.4712	81.0292	70.9406	2022/8/13	2:00:00	19.9052	85.2879	67.0340
2022/4/13	3:00:00	13.6194	81.9816	56.6621	2022/6/13	3:00:00	17.3688	81.2966	62.7224	2022/7/13	3:00:00	21.5229	83.2981	69.5709	2022/8/13	3:00:00	19.1381	85.6270	65.7806
2022/4/13	4:00:00	13.4377	87.0513	56.3169	2022/6/13	4:00:00	18.4708	74.0324	64.2123	2022/7/13	4:00:00	21.5686	83.2588	69.6428	2022/8/13	4:00:00	19.7530	82.6124	66.6417
2022/4/13	5:00:00	12.8459	90.2081	55.2775	2022/6/13	5:00:00	18.1762	76.8971	63.8636	2022/7/13	5:00:00	21.4563	81.9116	69.3657	2022/8/13	5:00:00	19.5783	85.4734	66.5027
2022/4/13	6:00:00	13.0012	90.8039	55.5336	2022/6/13	6:00:00	17.7101	80.9944	63.2638	2022/7/13	6:00:00	21.5143	81.6964	69.4447	2022/8/13	6:00:00	20.3342	81.4764	67.5214
2022/4/13	7:00:00	12.0600	92.4746	53.8856	2022/6/13	7:00:00	18.5670	80.3257	64.6176	2022/7/13	7:00:00	21.6799	82.8067	69.7923	2022/8/13	7:00:00	21.8722	81.1629	69.9847
2022/4/13	8:00:00	11.2128	94.9150	52.3457	2022/6/13	8:00:00	18.5342	78.0213	64.4717	2022/7/13	8:00:00	21.4977	83.5928	69.5502	2022/8/13	8:00:00	23.0521	71.5650	71.0707
2022/4/13	9:00:00	13.2184	88.1460	55.9370	2022/6/13	9:00:00	20.5597	67.3910	67.0332	2022/7/13	9:00:00	23.9274	77.6135	72.9676	2022/8/13	9:00:00	27.1162	59.9017	75.7788
2022/4/13	10:00:00	12.5470	90.4456	54.7641	2022/6/13	10:00:00	19.5260	73.8172	65.8296	2022/7/13	10:00:00	23.4683	81.7515	72.6128	2022/8/13	10:00:00	26.4856	62.4449	75.1973
2022/4/13	11:00:00	11.9879	93.0699	53.7468	2022/6/13	11:00:00	18.6530	78.2857	64.6706	2022/7/13	11:00:00	23.1754	82.8551	72.2337	2022/8/13	11:00:00	25.1847	68.9722	74.0333
2022/4/13	12:00:00	13.3915	87.7259	56.2326	2022/6/13	12:00:00	20.1839	69.5026	66.5982	2022/7/13	12:00:00	24.6461	80.1113	74.3543	2022/8/13	12:00:00	27.7005	64.5538	77.2092
2022/4/13	13:00:00	13.3517	87.6867	56.1663	2022/6/13	13:00:00	20.0275	69.2571	66.3503	2022/7/13	13:00:00	24.0188	80.1743	73.3546	2022/8/13	13:00:00	27.2264	67.6419	76.9129
2022/4/13	14:00:00	13.1388	86.4285	55.8253	2022/6/13	14:00:00	19.7980	68.2835	65.9554	2022/7/13	14:00:00	23.7984	81.5412	73.1278	2022/8/13	14:00:00	27.2560	67.7933	76.9758
2022/4/13	15:00:00	12.1594	91.7442	54.0737	2022/6/13	15:00:00	19.9501	68.1047	66.1717	2022/7/13	15:00:00	24.7703	81.8568	74.7318	2022/8/13	15:00:00	27.0549	64.7296	76.2956

2022/4/13	16:00:00	12.4094	90.0288	54.5378	2022/6/13	16:00:00	20.1468	68.6960	66.4971	2022/7/13	16:00:00	24.6240	80.8095	74.3893	2022/8/13	16:00:00	27.6656	62.6517	76.9096
2022/4/13	17:00:00	11.3209	90.2038	52.6805	2022/6/13	17:00:00	18.7282	70.8672	64.4753	2022/7/13	17:00:00	23.3130	81.5039	72.3394	2022/8/13	17:00:00	24.9299	71.3013	73.8947
2022/4/13	18:00:00	11.8369	87.0020	53.6419	2022/6/13	18:00:00	18.6261	79.1108	64.6622	2022/7/13	18:00:00	23.0292	81.8048	71.9061	2022/8/13	18:00:00	25.3562	72.9465	74.7186
2022/4/13	19:00:00	12.8308	87.0950	55.3016	2022/6/13	19:00:00	19.0373	75.4498	65.1509	2022/7/13	19:00:00	23.3769	80.5648	72.3598	2022/8/13	19:00:00	24.8944	75.5043	74.2757
2022/4/13	20:00:00	13.3356	84.7080	56.1719	2022/6/13	20:00:00	19.0803	73.9642	65.1497	2022/7/13	20:00:00	23.6371	78.7576	72.6136	2022/8/13	20:00:00	24.8331	76.0993	74.2415
2022/4/13	21:00:00	12.8932	86.6171	55.4133	2022/6/13	21:00:00	18.2149	75.0119	63.8541	2022/7/13	21:00:00	23.6844	78.3790	72.6541	2022/8/13	21:00:00	23.6547	77.8441	72.5583
2022/4/13	22:00:00	11.9906	90.7207	53.8085	2022/6/13	22:00:00	16.1523	78.5548	60.7116	2022/7/13	22:00:00	21.5095	88.2945	69.8984	2022/8/13	22:00:00	20.8320	87.1193	68.6830
2022/4/13	23:00:00	11.8944	92.0424	53.6108	2022/6/13	23:00:00	16.8388	75.1964	61.7218	2022/7/13	23:00:00	20.8661	89.0330	68.8617	2022/8/13	23:00:00	20.2455	89.5791	67.8434
2022/4/14	0:00:00	11.6659	94.0553	53.1622	2022/6/14	0:00:00	16.3448	75.0365	60.9509	2022/7/14	0:00:00	20.9370	88.9052	68.9735	2022/8/14	0:00:00	19.6299	90.2802	66.8349
2022/4/14	1:00:00	11.7423	92.5830	53.3345	2022/6/14	1:00:00	15.9986	73.2933	60.3866	2022/7/14	1:00:00	20.6844	89.5875	68.5887	2022/8/14	1:00:00	20.2627	85.9726	67.6648
2022/4/14	2:00:00	12.0589	91.3452	53.9104	2022/6/14	2:00:00	16.8791	69.5081	61.6474	2022/7/14	2:00:00	20.7892	88.4864	68.6974	2022/8/14	2:00:00	20.6718	82.4616	68.1279
2022/4/14	3:00:00	11.0338	93.4683	52.0814	2022/6/14	3:00:00	15.9438	71.1014	60.2698	2022/7/14	3:00:00	21.4300	83.3364	69.4215	2022/8/14	3:00:00	20.0208	84.8839	67.2029
2022/4/14	4:00:00	10.3253	96.7547	50.7179	2022/6/14	4:00:00	16.2115	70.1841	60.6590	2022/7/14	4:00:00	21.7052	82.7950	69.8326	2022/8/14	4:00:00	20.0353	83.7447	67.1638
2022/4/14	5:00:00	11.0032	95.3087	51.9655	2022/6/14	5:00:00	15.7454	66.6275	59.9119	2022/7/14	5:00:00	22.5303	79.1582	70.8862	2022/8/14	5:00:00	21.0572	83.8379	68.8449
2022/4/14	6:00:00	11.0886	95.5921	52.1060	2022/6/14	6:00:00	16.7012	66.4725	61.3130	2022/7/14	6:00:00	22.1250	81.7175	70.4348	2022/8/14	6:00:00	21.5894	81.6614	69.5638
2022/4/14	7:00:00	11.4069	93.8423	52.7175	2022/6/14	7:00:00	18.2756	78.2818	64.0724	2022/7/14	7:00:00	21.5982	86.7358	69.9374	2022/8/14	7:00:00	22.5135	81.2092	71.0232
2022/4/14	8:00:00	11.1940	93.8038	52.3486	2022/6/14	8:00:00	20.0968	62.9320	66.1000	2022/7/14	8:00:00	20.7553	91.9210	68.8548	2022/8/14	8:00:00	22.7887	72.8926	70.7804
2022/4/14	9:00:00	12.6018	94.0221	54.7923	2022/6/14	9:00:00	23.8430	44.8466	69.7856	2022/7/14	9:00:00	24.5472	77.3084	73.9154	2022/8/14	9:00:00	26.0814	64.4004	74.8452
2022/4/14	10:00:00	12.1148	93.3869	53.9592	2022/6/14	10:00:00	22.9388	50.7759	69.1504	2022/7/14	10:00:00	24.7364	78.0061	74.2846	2022/8/14	10:00:00	25.2895	69.3507	74.2304
2022/4/14	11:00:00	11.8471	95.6857	53.4357	2022/6/14	11:00:00	22.3841	54.7719	68.7363	2022/7/14	11:00:00	24.3311	80.0656	73.8449	2022/8/14	11:00:00	24.9433	73.5544	74.1493
2022/4/14	12:00:00	12.4835	93.9710	54.5874	2022/6/14	12:00:00	23.6473	52.0846	70.1997	2022/7/14	12:00:00	27.0032	69.7329	76.8426	2022/8/14	12:00:00	27.4893	69.9713	77.6027
2022/4/14	13:00:00	12.3739	95.4743	54.3658	2022/6/14	13:00:00	23.4802	52.6032	70.0245	2022/7/14	13:00:00	27.1763	68.6477	76.9656	2022/8/14	13:00:00	27.2291	70.6962	77.3034
2022/4/14	14:00:00	12.5766	96.7099	54.6987	2022/6/14	14:00:00	23.1770	51.4107	69.5179	2022/7/14	14:00:00	27.0151	69.3923	76.8180	2022/8/14	14:00:00	26.1771	71.7079	75.8325
2022/4/14	15:00:00	12.0750	95.0745	53.8506	2022/6/14	15:00:00	23.0114	49.6194	69.1476	2022/7/14	15:00:00	26.9764	70.9536	76.9538	2022/8/14	15:00:00	26.3340	69.7093	75.8358
2022/4/14	16:00:00	11.9213	95.2772	53.5763	2022/6/14	16:00:00	22.7969	49.5293	68.8611	2022/7/14	16:00:00	26.8372	72.7358	76.9619	2022/8/14	16:00:00	26.9555	66.5216	76.3732
2022/4/14	17:00:00	10.2157	99.7418	50.3990	2022/6/14	17:00:00	20.0581	67.6430	66.3064	2022/7/14	17:00:00	24.2854	82.9185	74.0495	2022/8/14	17:00:00	24.4283	72.4647	73.2494
2022/4/14	18:00:00	10.6849	98.5979	51.2851	2022/6/14	18:00:00	19.8173	76.5047	66.4214	2022/7/14	18:00:00	24.2322	81.3076	73.8067	2022/8/14	18:00:00	24.3348	74.0727	73.2640
2022/4/14	19:00:00	10.8801	97.2056	51.6827	2022/6/14	19:00:00	21.6165	58.9636	67.9960	2022/7/14	19:00:00	24.3117	82.3885	74.0407	2022/8/14	19:00:00	23.9063	77.4372	72.9179
2022/4/14	20:00:00	11.1832	96.6271	52.2387	2022/6/14	20:00:00	20.9666	63.3599	67.3740	2022/7/14	20:00:00	25.0030	81.1167	75.0316	2022/8/14	20:00:00	23.6236	77.8686	72.5113
2022/4/14	21:00:00	10.8715	97.5948	51.6537	2022/6/14	21:00:00	19.9533	68.1950	66.1814	2022/7/14	21:00:00	24.9289	79.8873	74.7843	2022/8/14	21:00:00	22.8446	80.8021	71.5238
2022/4/14	22:00:00	9.9039	99.9926	49.8273	2022/6/14	22:00:00	17.5478	74.6208	62.8063	2022/7/14	22:00:00	23.4554	84.0120	72.7935	2022/8/14	22:00:00	21.5550	84.2611	69.6910
2022/4/14	23:00:00	9.9985	100.0000	49.9972	2022/6/14	23:00:00	17.3005	73.9263	62.4037	2022/7/14	23:00:00	23.9392	79.2897	73.1439	2022/8/14	23:00:00	21.7813	82.6999	69.9498
2022/4/15	0:00:00	9.8501	99.9939	49.7305	2022/6/15	0:00:00	16.7366	73.9302	61.5343	2022/7/15	0:00:00	23.9537	77.3147	72.9811	2022/8/15	0:00:00	21.0610	83.4912	68.8284
2022/4/15	1:00:00	9.9910	99.9984	49.9838	2022/6/15	1:00:00	16.4603	73.0756	61.0912	2022/7/15	1:00:00	23.7038	78.4551	72.6919	2022/8/15	1:00:00	21.4652	80.3774	69.2735
2022/4/15	2:00:00	9.8018	99.9849	49.6438	2022/6/15	2:00:00	16.4141	71.8479	60.9965	2022/7/15	2:00:00	23.4538	81.0957	72.5307	2022/8/15	2:00:00	21.4706	80.2820	69.2755
2022/4/15	3:00:00	9.1389	99.7286	48.4643	2022/6/15	3:00:00	15.6540	73.5059	59.8600	2022/7/15	3:00:00	22.5185	81.8131	71.0795	2022/8/15	3:00:00	20.4562	82.8766	67.8020
2022/4/15	4:00:00	9.0798	99.9500	48.3463	2022/6/15	4:00:00	15.7513	71.9559	59.9895	2022/7/15	4:00:00	22.3690	80.2944	70.7183	2022/8/15	4:00:00	20.4600	83.0063	67.8159
2022/4/15	5:00:00	8.3547	99.9437	47.0418	2022/6/15	5:00:00	17.6085	63.2057	62.5428	2022/7/15	5:00:00	23.6506	77.1142	72.4852	2022/8/15	5:00:00	20.8642	86.1435	68.6749
2022/4/15	6:00:00	8.3326	99.9857	46.9996	2022/6/15	6:00:00	18.0445	65.7050	63.2578	2022/7/15	6:00:00	22.8991	83.5323	71.8400	2022/8/15	6:00:00	21.3039	84.0852	69.2663
2022/4/15	7:00:00	8.0440	99.9946	46.4795	2022/6/15	7:00:00	19.0002	67.1804	64.7202	2022/7/15	7:00:00	23.3533	82.5360	72.4956	2022/8/15	7:00:00	21.5002	87.8346	69.8505
2022/4/15	8:00:00	7.6666	99.9975	45.8000	2022/6/15	8:00:00	19.0965	60.3659	64.5483	2022/7/15	8:00:00	23.3162	81.3749	72.3333	2022/8/15	8:00:00	22.1017	77.7949	70.0998
2022/4/15	9:00:00	7.5086	99.4850	45.5508	2022/6/15	9:00:00	21.9342	54.7397	68.1255	2022/7/15	9:00:00	27.0742	71.1258	77.1233	2022/8/15	9:00:00	25.6384	66.2524	74.4092
2022/4/15	10:00:00	7.5096	99.9837	45.5185	2022/6/15	10:00:00	21.3085	61.2649	67.7230	2022/7/15	10:00:00	26.0561	76.9238	76.2482	2022/8/15	10:00:00	25.5121	69.2155	74.5487
2022/4/15	11:00:00	7.2554	99.9929	45.0602	2022/6/15	11:00:00	21.1026	67.2455	67.8256	2022/7/15	11:00:00	25.3696	81.4912	75.6634	2022/8/15	11:00:00	25.2175	70.3848	74.2329

2022/4/15	12:00:00	7.3070	100.0000	45.1525	2022/6/15	12:00:00	21.4918	69.8498	68.5817	2022/7/15	12:00:00	28.0149	77.9172	79.4601	2022/8/15	12:00:00	27.2791	62.7403	76.3680
2022/4/15	13:00:00	7.0409	99.9726	44.6756	2022/6/15	13:00:00	22.1062	74.4891	69.8561	2022/7/15	13:00:00	28.6347	78.7593	80.5585	2022/8/15	13:00:00	27.3033	61.2063	76.2074
2022/4/15	14:00:00	7.2882	99.8769	45.1274	2022/6/15	14:00:00	21.3009	76.4977	68.7464	2022/7/15	14:00:00	28.7357	75.8686	80.3101	2022/8/15	14:00:00	26.5458	63.3661	75.3936
2022/4/15	15:00:00	5.9991	98.8155	42.8975	2022/6/15	15:00:00	20.5538	79.3813	67.7497	2022/7/15	15:00:00	29.1142	73.4121	80.5442	2022/8/15	15:00:00	26.5813	63.8161	75.4987
2022/4/15	16:00:00	5.8615	99.1285	42.6248	2022/6/15	16:00:00	20.3661	79.7013	67.4690	2022/7/15	16:00:00	28.6100	73.9952	79.8511	2022/8/15	16:00:00	26.7372	63.5258	75.6881
2022/4/15	17:00:00	5.1751	100.0000	41.3151	2022/6/15	17:00:00	19.1900	71.7100	65.2129	2022/7/15	17:00:00	24.8579	79.1883	74.5987	2022/8/15	17:00:00	23.7698	74.2820	72.4113
2022/4/15	18:00:00	5.0998	99.8058	41.1976	2022/6/15	18:00:00	19.9458	67.4706	66.1308	2022/7/15	18:00:00	25.3385	78.3766	75.2771	2022/8/15	18:00:00	24.5509	77.4602	73.9364
2022/4/15	19:00:00	5.1235	99.6869	41.2511	2022/6/15	19:00:00	20.9784	64.3237	67.4534	2022/7/15	19:00:00	25.1525	81.6426	75.3284	2022/8/15	19:00:00	25.3841	70.4780	74.4941
2022/4/15	20:00:00	5.2374	99.2691	41.4940	2022/6/15	20:00:00	19.8571	70.9446	66.1858	2022/7/15	20:00:00	24.6789	80.5366	74.4499	2022/8/15	20:00:00	24.5676	77.2055	73.9372
2022/4/15	21:00:00	5.1869	97.0943	41.6027	2022/6/15	21:00:00	18.5922	76.8485	64.5154	2022/7/15	21:00:00	24.5456	79.7432	74.1564	2022/8/15	21:00:00	23.4693	81.5745	72.5985
2022/4/15	22:00:00	4.5848	98.4057	40.4083	2022/6/15	22:00:00	16.8184	81.6928	61.8428	2022/7/15	22:00:00	23.4775	84.5671	72.8793	2022/8/15	22:00:00	21.8437	82.6418	70.0471
2022/4/15	23:00:00	4.6795	98.8658	40.5327	2022/6/15	23:00:00	17.3005	77.1111	62.4938	2022/7/15	23:00:00	23.5425	82.8759	72.8341	2022/8/15	23:00:00	22.2350	79.5816	70.4483
2022/4/16	0:00:00	4.5478	99.2136	40.2630	2022/6/16	0:00:00	16.9721	75.2777	61.9311	2022/7/16	0:00:00	22.8528	85.6689	71.9421	2022/8/16	0:00:00	20.9121	83.5676	68.5896
2022/4/16	1:00:00	4.5198	99.1875	40.2155	2022/6/16	1:00:00	17.2575	73.1061	62.3145	2022/7/16	1:00:00	22.7335	84.7792	71.6713	2022/8/16	1:00:00	20.3315	86.0711	67.7849
2022/4/16	2:00:00	4.4902	98.3222	40.2477	2022/6/16	2:00:00	17.4306	70.3915	62.4998	2022/7/16	2:00:00	22.9313	83.5980	71.8983	2022/8/16	2:00:00	19.8321	86.6569	66.9861
2022/4/16	3:00:00	4.0892	97.9738	39.5683	2022/6/16	3:00:00	16.8318	72.0819	61.6374	2022/7/16	3:00:00	21.3294	88.3204	69.5969	2022/8/16	3:00:00	19.0214	87.4792	65.6712
2022/4/16	4:00:00	4.0285	98.3471	39.4217	2022/6/16	4:00:00	16.9334	71.3170	61.7733	2022/7/16	4:00:00	21.6520	87.5931	70.0883	2022/8/16	4:00:00	18.8709	87.1824	65.4059
2022/4/16	5:00:00	4.0199	96.0822	39.6401	2022/6/16	5:00:00	18.1584	72.3480	63.6685	2022/7/16	5:00:00	21.4359	86.7772	69.6694	2022/8/16	5:00:00	21.3539	77.9870	68.9313
2022/4/16	6:00:00	4.1898	95.9733	39.9504	2022/6/16	6:00:00	17.9558	75.6369	63.4735	2022/7/16	6:00:00	21.3455	84.5900	69.3692	2022/8/16	6:00:00	21.2480	77.9360	68.7603
2022/4/16	7:00:00	4.0817	96.7595	39.6795	2022/6/16	7:00:00	18.2993	74.0212	63.9473	2022/7/16	7:00:00	21.1590	86.5731	69.1937	2022/8/16	7:00:00	22.1194	79.9606	70.2923
2022/4/16	8:00:00	3.9059	97.5739	39.2838	2022/6/16	8:00:00	17.9988	66.8407	63.2310	2022/7/16	8:00:00	21.0671	89.2430	69.2155	2022/8/16	8:00:00	22.4866	71.0874	70.1740
2022/4/16	9:00:00	5.1014	94.0289	41.7349	2022/6/16	9:00:00	20.5349	56.2165	66.3229	2022/7/16	9:00:00	23.0259	80.9067	71.8246	2022/8/16	9:00:00	26.0808	62.8874	74.6701
2022/4/16	10:00:00	4.4445	96.4750	40.3492	2022/6/16	10:00:00	19.9474	60.3966	65.7478	2022/7/16	10:00:00	22.5335	83.6527	71.2512	2022/8/16	10:00:00	25.1912	67.6496	73.9022
2022/4/16	11:00:00	4.5596	94.3181	40.7633	2022/6/16	11:00:00	19.7464	65.1080	65.7120	2022/7/16	11:00:00	21.9654	86.4640	70.5298	2022/8/16	11:00:00	24.3654	70.9928	73.0088
2022/4/16	12:00:00	6.0244	89.0426	43.7573	2022/6/16	12:00:00	20.4624	63.8821	66.6804	2022/7/16	12:00:00	23.5248	82.1425	72.7393	2022/8/16	12:00:00	26.4647	65.5465	75.5364
2022/4/16	13:00:00	5.8352	89.1683	43.4265	2022/6/16	13:00:00	20.5570	65.3053	66.9030	2022/7/16	13:00:00	22.8636	85.0941	71.9120	2022/8/16	13:00:00	27.3635	63.0459	76.5279
2022/4/16	14:00:00	5.7820	89.4731	43.3103	2022/6/16	14:00:00	20.6795	67.0722	67.1906	2022/7/16	14:00:00	22.9835	84.0763	72.0241	2022/8/16	14:00:00	27.0081	64.9123	76.2504
2022/4/16	15:00:00	5.9196	93.3262	43.2185	2022/6/16	15:00:00	20.9784	67.5820	67.6641	2022/7/16	15:00:00	23.8322	79.2155	72.9663	2022/8/16	15:00:00	27.5199	63.7492	76.8433
2022/4/16	16:00:00	5.7642	94.0014	42.8911	2022/6/16	16:00:00	21.1692	66.3667	67.8655	2022/7/16	16:00:00	23.8693	78.8597	72.9923	2022/8/16	16:00:00	27.7941	62.2227	77.0367
2022/4/16	17:00:00	4.2107	100.0000	39.5793	2022/6/16	17:00:00	18.8718	73.9489	64.8273	2022/7/16	17:00:00	21.7804	90.8158	70.5377	2022/8/16	17:00:00	24.8396	70.4844	73.6737
2022/4/16	18:00:00	4.3435	100.0000	39.8183	2022/6/16	18:00:00	19.2002	71.2080	65.2048	2022/7/16	18:00:00	21.9148	90.3932	70.7362	2022/8/16	18:00:00	25.5035	73.3657	74.9902
2022/4/16	19:00:00	4.5343	99.8434	40.1771	2022/6/16	19:00:00	19.5303	69.9505	65.6415	2022/7/16	19:00:00	22.3529	90.9730	71.5285	2022/8/16	19:00:00	24.9390	73.4910	74.1361
2022/4/16	20:00:00	4.6988	99.4211	40.5137	2022/6/16	20:00:00	20.0436	68.1278	66.3118	2022/7/16	20:00:00	22.8469	90.0265	72.2948	2022/8/16	20:00:00	24.4272	76.6015	73.6566
2022/4/16	21:00:00	4.8095	98.3022	40.8191	2022/6/16	21:00:00	19.3599	73.1583	65.5416	2022/7/16	21:00:00	22.4997	87.9735	71.5403	2022/8/16	21:00:00	23.3215	79.2211	72.1525
2022/4/16	22:00:00	4.0446	99.8407	39.2967	2022/6/16	22:00:00	17.2946	80.0703	62.5680	2022/7/16	22:00:00	21.4015	91.3978	69.9301	2022/8/16	22:00:00	19.9455	89.6337	67.3373
2022/4/16	23:00:00	4.3274	99.3394	39.8554	2022/6/16	23:00:00	17.6736	78.6577	63.1302	2022/7/16	23:00:00	22.0659	88.0230	70.8149	2022/8/16	23:00:00	19.8493	89.9322	67.1900
2022/4/17	0:00:00	4.3424	99.6459	39.8517	2022/6/17	0:00:00	17.3215	80.4864	62.6229	2022/7/17	0:00:00	22.1417	84.1089	70.6440	2022/8/17	0:00:00	19.5896	89.2329	66.7129
2022/4/17	1:00:00	4.4177	99.6547	39.9861	2022/6/17	1:00:00	17.0818	81.5176	62.2646	2022/7/17	1:00:00	22.4062	82.5003	70.9517	2022/8/17	1:00:00	19.9304	86.6482	67.1496
2022/4/17	2:00:00	4.7225	99.6938	40.5299	2022/6/17	2:00:00	17.1941	79.5772	62.3934	2022/7/17	2:00:00	22.7152	80.1418	71.2614	2022/8/17	2:00:00	20.1939	84.9027	67.4896
2022/4/17	3:00:00	4.5714	99.6040	40.2672	2022/6/17	3:00:00	14.0693	90.6742	57.3594	2022/7/17	3:00:00	21.6267	82.7450	69.7012	2022/8/17	3:00:00	19.7348	85.0226	66.7381
2022/4/17	4:00:00	4.5537	99.7276	40.2233	2022/6/17	4:00:00	13.9645	90.4620	57.1814	2022/7/17	4:00:00	21.9256	82.8736	70.1976	2022/8/17	4:00:00	20.2046	83.0204	67.4000
2022/4/17	5:00:00	4.4311	99.4124	40.0343	2022/6/17	5:00:00	18.6234	70.6957	64.3098	2022/7/17	5:00:00	22.1653	79.3735	70.3209	2022/8/17	5:00:00	21.9275	81.7178	70.1152
2022/4/17	6:00:00	4.4338	99.3139	40.0488	2022/6/17	6:00:00	17.7564	77.4716	63.2228	2022/7/17	6:00:00	22.0820	80.0186	70.2368	2022/8/17	6:00:00	21.9899	82.2830	70.2583
2022/4/17	7:00:00	4.2548	99.2242	39.7369	2022/6/17	7:00:00	17.8182	77.6788	63.3272	2022/7/17	7:00:00	21.7966	77.3331	69.5840	2022/8/17	7:00:00	22.3995	81.9414	70.8969

2022/4/17	8:00:00	4.4650	98.7794	40.1575	2022/6/17	8:00:00	17.4661	79.9864	62.8403	2022/7/17	8:00:00	21.3751	74.7014	68.7394	2022/8/17	8:00:00	22.8022	77.0449	71.1446
2022/4/17	9:00:00	5.9314	93.8056	43.1986	2022/6/17	9:00:00	20.6930	69.7535	67.3763	2022/7/17	9:00:00	23.0023	71.0218	70.9490	2022/8/17	9:00:00	25.8303	69.9831	75.1111
2022/4/17	10:00:00	5.7761	93.2111	42.9795	2022/6/17	10:00:00	20.2033	75.3876	66.9627	2022/7/17	10:00:00	23.1990	69.2394	71.0922	2022/8/17	10:00:00	25.1702	72.8833	74.4270
2022/4/17	11:00:00	5.5540	94.8841	42.4475	2022/6/17	11:00:00	20.1753	77.5468	67.0417	2022/7/17	11:00:00	22.6846	69.5577	70.3489	2022/8/17	11:00:00	24.8191	75.7830	74.1871
2022/4/17	12:00:00	7.0645	88.3472	45.5675	2022/6/17	12:00:00	21.3601	74.4413	68.6983	2022/7/17	12:00:00	24.0559	64.7775	71.9490	2022/8/17	12:00:00	26.9458	71.0329	76.9174
2022/4/17	13:00:00	6.5055	92.3264	44.3130	2022/6/17	13:00:00	21.9627	76.6710	69.7964	2022/7/17	13:00:00	23.5764	66.2329	71.3847	2022/8/17	13:00:00	26.9248	71.1946	76.9056
2022/4/17	14:00:00	6.6490	93.0383	44.5055	2022/6/17	14:00:00	22.4346	75.2085	70.4213	2022/7/17	14:00:00	23.2705	66.6490	70.9728	2022/8/17	14:00:00	25.1396	70.5349	74.1314
2022/4/17	15:00:00	7.1785	91.1803	45.5558	2022/6/17	15:00:00	21.3794	75.1184	68.7747	2022/7/17	15:00:00	23.5721	63.8284	71.1611	2022/8/17	15:00:00	26.2174	67.9465	75.4554
2022/4/17	16:00:00	6.7893	94.2146	44.6592	2022/6/17	16:00:00	22.0632	75.8118	69.8893	2022/7/17	16:00:00	23.5753	63.4282	71.1296	2022/8/17	16:00:00	26.6383	66.4876	75.9034
2022/4/17	17:00:00	5.3944	98.4536	41.8485	2022/6/17	17:00:00	18.4767	85.7246	64.6882	2022/7/17	17:00:00	21.5880	71.5230	68.8445	2022/8/17	17:00:00	24.0751	73.3993	72.7990
2022/4/17	18:00:00	5.7685	97.8002	42.5723	2022/6/17	18:00:00	18.6363	82.8408	64.8333	2022/7/17	18:00:00	21.7197	73.7965	69.2081	2022/8/17	18:00:00	24.2628	75.8117	73.3218
2022/4/17	19:00:00	6.5216	98.5808	43.8502	2022/6/17	19:00:00	19.3851	82.8646	66.0551	2022/7/17	19:00:00	21.9299	78.3898	69.8723	2022/8/17	19:00:00	23.7677	79.2812	72.8694
2022/4/17	20:00:00	7.1161	97.8609	44.9642	2022/6/17	20:00:00	19.7136	82.3837	66.5655	2022/7/17	20:00:00	22.3115	77.5765	70.4143	2022/8/17	20:00:00	24.1418	77.4356	73.2890
2022/4/17	21:00:00	7.7112	96.1333	46.1379	2022/6/17	21:00:00	19.2093	84.4294	65.8423	2022/7/17	21:00:00	21.8057	77.5139	69.6115	2022/8/17	21:00:00	22.9350	82.5486	71.8160
2022/4/17	22:00:00	6.8646	96.0162	44.6552	2022/6/17	22:00:00	17.8289	88.8915	63.7198	2022/7/17	22:00:00	20.7714	79.1276	68.0812	2022/8/17	22:00:00	20.9594	87.6977	68.9334
2022/4/17	23:00:00	6.9877	93.3808	45.0664	2022/6/17	23:00:00	18.2176	85.9632	64.2673	2022/7/17	23:00:00	20.5704	73.1544	67.3987	2022/8/17	23:00:00	21.0185	86.2783	68.9402
2022/4/18	0:00:00	7.3011	90.0262	45.8473	2022/6/18	0:00:00	18.2595	85.6083	64.3236	2022/7/18	0:00:00	20.2302	74.5204	66.9549	2022/8/18	0:00:00	21.7490	81.9498	69.8430
2022/4/18	1:00:00	7.4623	88.0172	46.2605	2022/6/18	1:00:00	18.6067	83.0146	64.7922	2022/7/18	1:00:00	20.2936	74.3172	67.0412	2022/8/18	1:00:00	21.9576	79.9009	70.0288
2022/4/18	2:00:00	7.5629	86.6861	46.5202	2022/6/18	2:00:00	18.7003	81.3876	64.8763	2022/7/18	2:00:00	20.0200	75.2008	66.6671	2022/8/18	2:00:00	22.3221	77.4854	70.4239
2022/4/18	3:00:00	7.7333	83.3289	47.0275	2022/6/18	3:00:00	17.8940	84.6458	63.6848	2022/7/18	3:00:00	19.5077	79.8564	66.1041	2022/8/18	3:00:00	21.0771	82.0270	68.7586
2022/4/18	4:00:00	7.9924	81.5513	47.5647	2022/6/18	4:00:00	17.5166	85.3516	63.0844	2022/7/18	4:00:00	19.6738	80.1693	66.3862	2022/8/18	4:00:00	21.0561	81.9324	68.7184
2022/4/18	5:00:00	8.0230	79.7456	47.7290	2022/6/18	5:00:00	18.4842	83.8715	64.6265	2022/7/18	5:00:00	20.8526	73.7205	67.8675	2022/8/18	5:00:00	21.7034	81.5649	69.7412
2022/4/18	6:00:00	8.2316	78.5514	48.1361	2022/6/18	6:00:00	18.0149	87.1324	63.9720	2022/7/18	6:00:00	20.1947	82.2636	67.3407	2022/8/18	6:00:00	21.7507	82.5157	69.8865
2022/4/18	7:00:00	8.1359	83.0211	47.7050	2022/6/18	7:00:00	17.7580	85.5482	63.4903	2022/7/18	7:00:00	19.7684	84.4249	66.7622	2022/8/18	7:00:00	22.3071	85.3550	71.0127
2022/4/18	8:00:00	8.1816	75.2452	48.2617	2022/6/18	8:00:00	16.8285	88.1634	62.0120	2022/7/18	8:00:00	19.3335	86.1641	66.1307	2022/8/18	8:00:00	22.7151	78.9717	71.1654
2022/4/18	9:00:00	10.3398	57.7360	52.3291	2022/6/18	9:00:00	19.4738	82.4773	66.1804	2022/7/18	9:00:00	19.8291	85.2881	66.9082	2022/8/18	9:00:00	25.1928	68.1696	73.9600
2022/4/18	10:00:00	9.4185	62.0587	50.8411	2022/6/18	10:00:00	19.0051	84.2247	65.4969	2022/7/18	10:00:00	19.5776	83.3168	66.3918	2022/8/18	10:00:00	24.9955	69.2998	73.7851
2022/4/18	11:00:00	9.4303	62.4612	50.8380	2022/6/18	11:00:00	18.6379	84.7001	64.9131	2022/7/18	11:00:00	19.7845	80.8074	66.5975	2022/8/18	11:00:00	24.6337	70.2727	73.3419
2022/4/18	12:00:00	11.4036	56.2029	53.8450	2022/6/18	12:00:00	19.6931	80.1986	66.4187	2022/7/18	12:00:00	20.1721	81.5948	67.2661	2022/8/18	12:00:00	26.4028	65.7684	75.4725
2022/4/18	13:00:00	11.2144	58.1592	53.5239	2022/6/18	13:00:00	19.4502	81.3173	66.0844	2022/7/18	13:00:00	20.1592	80.1442	67.1632	2022/8/18	13:00:00	25.8550	68.9722	75.0340
2022/4/18	14:00:00	11.0160	59.6317	53.1990	2022/6/18	14:00:00	20.2345	77.8772	67.1539	2022/7/18	14:00:00	20.3925	78.3158	67.4296	2022/8/18	14:00:00	25.8362	70.6008	75.1896
2022/4/18	15:00:00	10.2157	62.5741	51.9550	2022/6/18	15:00:00	19.9979	79.7115	66.8808	2022/7/18	15:00:00	20.2210	77.2308	67.0957	2022/8/18	15:00:00	25.8954	68.6838	75.0615
2022/4/18	16:00:00	10.4436	63.7423	52.2345	2022/6/18	16:00:00	19.3464	83.6268	66.0290	2022/7/18	16:00:00	20.1753	75.4309	66.9216	2022/8/18	16:00:00	26.2206	67.8324	75.4469
2022/4/18	17:00:00	8.0407	75.4026	48.0327	2022/6/18	17:00:00	17.7902	92.6533	63.7790	2022/7/18	17:00:00	18.0628	88.1507	64.0885	2022/8/18	17:00:00	23.6187	76.8523	72.4113
2022/4/18	18:00:00	8.2660	81.3068	48.0221	2022/6/18	18:00:00	18.0730	90.2938	64.1827	2022/7/18	18:00:00	18.1988	89.3369	64.3614	2022/8/18	18:00:00	23.7354	79.8533	72.8706
2022/4/18	19:00:00	8.6433	72.9930	49.1090	2022/6/18	19:00:00	18.3912	90.5280	64.7341	2022/7/18	19:00:00	18.8213	87.4580	65.3348	2022/8/18	19:00:00	23.3338	83.4963	72.5485
2022/4/18	20:00:00	8.8309	80.6097	48.9733	2022/6/18	20:00:00	19.2120	87.3104	65.9827	2022/7/18	20:00:00	19.0239	89.2477	65.7555	2022/8/18	20:00:00	23.9252	80.7839	73.2617
2022/4/18	21:00:00	8.3692	84.8626	47.9749	2022/6/18	21:00:00	19.0362	88.6039	65.7472	2022/7/18	21:00:00	18.9175	87.9804	65.5192	2022/8/18	21:00:00	23.7569	81.4333	73.0507
2022/4/18	22:00:00	6.9076	85.7155	45.4995	2022/6/18	22:00:00	18.5761	86.6143	64.8895	2022/7/18	22:00:00	18.3939	88.2554	64.6498	2022/8/18	22:00:00	21.7464	86.0045	70.1317
2022/4/18	23:00:00	7.3591	82.4289	46.4789	2022/6/18	23:00:00	18.8643	84.0354	65.2571	2022/7/18	23:00:00	18.8153	84.9151	65.2148	2022/8/18	23:00:00	21.9571	84.0437	70.3361
2022/4/19	0:00:00	7.3365	84.5771	46.2911	2022/6/19	0:00:00	18.8497	81.9396	65.1419	2022/7/19	0:00:00	18.7670	81.5072	64.9892	2022/8/19	0:00:00	22.1942	82.2573	70.5883
2022/4/19	1:00:00	6.9033	85.3947	45.5163	2022/6/19	1:00:00	18.9325	80.8632	65.2282	2022/7/19	1:00:00	18.7917	81.6754	65.0364	2022/8/19	1:00:00	22.3114	81.3247	70.7060
2022/4/19	2:00:00	7.4935	82.1350	46.7177	2022/6/19	2:00:00	19.1884	79.0431	65.5548	2022/7/19	2:00:00	18.9013	81.6758	65.2139	2022/8/19	2:00:00	22.3952	80.9354	70.8108
2022/4/19	3:00:00	5.7406	84.9413	43.6306	2022/6/19	3:00:00	18.4767	82.6252	64.5644	2022/7/19	3:00:00	18.2240	81.5749	64.1138	2022/8/19	3:00:00	21.7254	82.9915	69.8797

2022/4/19	4:00:00	5.7212	82.4927	43.8101	2022/6/19	4:00:00	18.8067	80.4544	65.0080	2022/7/19	4:00:00	18.8954	74.5276	64.8893	2022/8/19	4:00:00	21.7270	82.0742	69.8162
2022/4/19	5:00:00	7.5274	73.9118	47.3358	2022/6/19	5:00:00	19.6899	77.1636	66.2559	2022/7/19	5:00:00	17.6026	89.1853	63.3466	2022/8/19	5:00:00	21.4055	79.8079	69.1384
2022/4/19	6:00:00	7.4790	74.3572	47.2305	2022/6/19	6:00:00	18.9932	79.8630	65.2810	2022/7/19	6:00:00	17.4354	90.5329	63.1035	2022/8/19	6:00:00	21.4706	79.7479	69.2384
2022/4/19	7:00:00	7.7333	77.9965	47.3818	2022/6/19	7:00:00	18.0326	84.0812	63.8933	2022/7/19	7:00:00	17.0946	92.8463	62.5826	2022/8/19	7:00:00	21.7905	83.4339	70.0181
2022/4/19	8:00:00	7.6311	73.4101	47.5296	2022/6/19	8:00:00	17.2903	89.3662	62.8230	2022/7/19	8:00:00	16.9113	92.7345	62.2630	2022/8/19	8:00:00	21.9679	81.2413	70.1450
2022/4/19	9:00:00	10.8252	60.8976	52.8864	2022/6/19	9:00:00	20.9311	78.3346	68.2847	2022/7/19	9:00:00	18.8153	86.4449	65.2810	2022/8/19	9:00:00	24.5445	72.6122	73.4415
2022/4/19	10:00:00	10.0560	65.7773	51.5876	2022/6/19	10:00:00	20.6011	80.4156	67.8882	2022/7/19	10:00:00	18.3600	90.6768	64.6866	2022/8/19	10:00:00	24.7240	72.8621	73.7414
2022/4/19	11:00:00	10.2796	69.7439	51.7508	2022/6/19	11:00:00	20.1560	81.1485	67.2148	2022/7/19	11:00:00	17.6483	94.7737	63.6012	2022/8/19	11:00:00	24.6461	74.6779	73.8055
2022/4/19	12:00:00	11.8353	66.6657	54.1646	2022/6/19	12:00:00	22.1782	74.6818	69.9823	2022/7/19	12:00:00	18.3364	92.4627	64.7150	2022/8/19	12:00:00	26.2593	67.8932	75.5113
2022/4/19	13:00:00	12.1728	67.9942	54.6309	2022/6/19	13:00:00	22.1282	75.1272	69.9388	2022/7/19	13:00:00	18.8761	90.4188	65.5566	2022/8/19	13:00:00	25.9964	66.2979	74.9392
2022/4/19	14:00:00	11.9568	70.0665	54.2594	2022/6/19	14:00:00	22.6754	72.7092	70.5920	2022/7/19	14:00:00	19.3572	88.2562	66.2717	2022/8/19	14:00:00	26.1039	64.8013	74.9241
2022/4/19	15:00:00	11.8369	71.1764	54.0505	2022/6/19	15:00:00	22.3884	74.9493	70.3290	2022/7/19	15:00:00	20.2581	86.0515	67.6618	2022/8/19	15:00:00	26.6566	64.4548	75.6844
2022/4/19	16:00:00	12.1696	71.1623	54.5548	2022/6/19	16:00:00	21.9922	77.0167	69.8686	2022/7/19	16:00:00	20.9892	82.1615	68.6247	2022/8/19	16:00:00	26.6679	64.6490	75.7243
2022/4/19	17:00:00	10.0442	81.1412	50.9011	2022/6/19	17:00:00	20.2624	85.1591	67.6175	2022/7/19	17:00:00	19.6561	89.7301	66.8510	2022/8/19	17:00:00	23.9934	75.1454	72.8386
2022/4/19	18:00:00	10.1995	76.9393	51.3283	2022/6/19	18:00:00	19.8082	84.5029	66.8318	2022/7/19	18:00:00	19.5464	87.0724	66.5305	2022/8/19	18:00:00	24.1375	79.6921	73.4987
2022/4/19	19:00:00	11.0962	80.1009	52.6327	2022/6/19	19:00:00	20.3747	84.2614	67.7505	2022/7/19	19:00:00	19.0997	87.7691	65.8158	2022/8/19	19:00:00	23.8123	80.1457	73.0208
2022/4/19	20:00:00	11.8030	76.1749	53.8685	2022/6/19	20:00:00	20.8645	81.8360	68.4016	2022/7/19	20:00:00	19.6157	86.6678	66.6258	2022/8/19	20:00:00	23.3424	81.3359	72.3723
2022/4/19	21:00:00	11.5875	76.9517	53.5093	2022/6/19	21:00:00	20.5790	83.0277	68.0114	2022/7/19	21:00:00	19.1959	86.1146	65.8995	2022/8/19	21:00:00	22.2103	83.9590	70.7453
2022/4/19	22:00:00	6.8737	88.9888	45.1980	2022/6/19	22:00:00	19.2330	90.0379	66.1471	2022/7/19	22:00:00	17.2989	91.4168	62.8955	2022/8/19	22:00:00	20.4696	87.4370	68.0960
2022/4/19	23:00:00	7.4301	80.5105	46.7275	2022/6/19	23:00:00	19.7415	85.9934	66.8002	2022/7/19	23:00:00	17.1156	90.4203	62.5547	2022/8/19	23:00:00	20.1127	87.5657	67.5051
2022/4/20	0:00:00	7.0452	77.3520	46.3404	2022/6/20	0:00:00	19.1217	87.0404	65.8190	2022/7/20	0:00:00	17.6746	88.2671	63.4392	2022/8/20	0:00:00	19.7299	88.4095	66.9074
2022/4/20	1:00:00	7.4553	72.2162	47.3420	2022/6/20	1:00:00	19.8184	80.7639	66.6497	2022/7/20	1:00:00	17.6558	87.7001	63.3895	2022/8/20	1:00:00	19.9407	86.3190	67.1488
2022/4/20	2:00:00	6.7538	71.9571	46.2920	2022/6/20	2:00:00	19.8646	78.3730	66.5958	2022/7/20	2:00:00	17.7682	85.6493	63.5105	2022/8/20	2:00:00	20.0831	85.1189	67.3189
2022/4/20	3:00:00	5.1590	74.3059	43.6481	2022/6/20	3:00:00	18.3509	85.5032	64.4709	2022/7/20	3:00:00	16.5221	86.7189	61.4667	2022/8/20	3:00:00	18.9843	88.0446	65.6344
2022/4/20	4:00:00	4.8913	70.3258	43.6108	2022/6/20	4:00:00	17.9606	86.2551	63.8507	2022/7/20	4:00:00	15.9438	87.4913	60.5131	2022/8/20	4:00:00	19.1988	86.3246	65.9142
2022/4/20	5:00:00	3.2534	78.1690	40.2747	2022/6/20	5:00:00	19.8587	79.3731	66.6400	2022/7/20	5:00:00	17.2516	84.0428	62.6094	2022/8/20	5:00:00	19.2139	86.3783	65.9418
2022/4/20	6:00:00	2.9528	77.9326	39.8257	2022/6/20	6:00:00	19.8555	79.8939	66.6628	2022/7/20	6:00:00	18.0020	81.3725	63.7476	2022/8/20	6:00:00	19.6558	83.9216	66.5508
2022/4/20	7:00:00	4.0446	83.4256	40.9868	2022/6/20	7:00:00	19.2034	84.2844	65.8257	2022/7/20	7:00:00	18.0698	81.6913	63.8685	2022/8/20	7:00:00	21.1110	84.4865	68.9759
2022/4/20	8:00:00	7.4016	77.2232	46.9110	2022/6/20	8:00:00	18.7180	88.3336	65.1989	2022/7/20	8:00:00	17.8907	85.2054	63.6986	2022/8/20	8:00:00	22.6006	80.2288	71.0846
2022/4/20	9:00:00	14.3741	58.3836	57.9024	2022/6/20	9:00:00	20.7344	84.0627	68.3294	2022/7/20	9:00:00	21.4359	77.1964	69.0062	2022/8/20	9:00:00	26.5518	62.0827	75.2483
2022/4/20	10:00:00	14.9423	54.6446	58.6726	2022/6/20	10:00:00	19.7136	88.1695	66.8673	2022/7/20	10:00:00	21.2020	82.4487	68.9895	2022/8/20	10:00:00	25.6556	66.3883	74.4495
2022/4/20	11:00:00	15.0960	56.5121	58.8923	2022/6/20	11:00:00	20.2522	91.3781	67.9582	2022/7/20	11:00:00	20.3807	86.9721	67.9196	2022/8/20	11:00:00	25.1906	69.8128	74.1316
2022/4/20	12:00:00	18.0789	51.9218	62.8121	2022/6/20	12:00:00	22.6679	82.9527	71.4144	2022/7/20	12:00:00	21.9019	82.3917	70.1234	2022/8/20	12:00:00	27.6221	63.4870	76.9563
2022/4/20	13:00:00	17.9042	55.5714	62.7058	2022/6/20	13:00:00	22.6873	80.1413	71.2166	2022/7/20	13:00:00	21.3278	83.7511	69.2828	2022/8/20	13:00:00	27.3382	65.5125	76.8065
2022/4/20	14:00:00	18.0079	56.5127	62.8801	2022/6/20	14:00:00	22.7599	78.2812	71.1798	2022/7/20	14:00:00	21.5289	82.8669	69.5503	2022/8/20	14:00:00	27.7790	64.9139	77.3704
2022/4/20	15:00:00	19.0019	58.0243	64.3095	2022/6/20	15:00:00	22.3325	78.6257	70.5293	2022/7/20	15:00:00	22.1428	82.1939	70.4999	2022/8/20	15:00:00	28.5461	61.6344	78.0270
2022/4/20	16:00:00	19.0846	58.5415	64.4478	2022/6/20	16:00:00	23.3517	73.1738	71.6674	2022/7/20	16:00:00	21.8664	84.8174	70.2440	2022/8/20	16:00:00	28.3989	63.0171	78.0088
2022/4/20	17:00:00	15.1863	71.7837	59.1282	2022/6/20	17:00:00	21.6740	81.0461	69.6566	2022/7/20	17:00:00	20.6177	90.9252	68.5573	2022/8/20	17:00:00	24.4267	73.6856	73.3676
2022/4/20	18:00:00	15.0681	75.4776	58.9711	2022/6/20	18:00:00	21.6106	80.8882	69.5431	2022/7/20	18:00:00	20.6709	88.2336	68.4824	2022/8/20	18:00:00	24.7976	76.0782	74.1838
2022/4/20	19:00:00	16.1142	70.9041	60.5245	2022/6/20	19:00:00	22.0970	77.5674	70.0752	2022/7/20	19:00:00	21.8455	86.5686	70.3377	2022/8/20	19:00:00	24.5084	77.0697	73.8305
2022/4/20	20:00:00	16.1346	67.9811	60.5065	2022/6/20	20:00:00	21.7509	80.4752	69.7393	2022/7/20	20:00:00	21.3945	86.4863	69.5802	2022/8/20	20:00:00	24.7197	75.9059	74.0445
2022/4/20	21:00:00	14.9735	68.8094	58.7889	2022/6/20	21:00:00	21.7197	81.4485	69.7593	2022/7/20	21:00:00	20.1947	86.8188	67.6000	2022/8/20	21:00:00	23.9241	78.0156	73.0002
2022/4/20	22:00:00	12.5749	77.7128	55.0474	2022/6/20	22:00:00	20.3038	86.3624	67.7558	2022/7/20	22:00:00	17.9219	95.3771	64.1003	2022/8/20	22:00:00	21.9372	82.3762	70.1797
2022/4/20	23:00:00	12.0766	75.1954	54.3194	2022/6/20	23:00:00	21.0569	82.0962	68.7304	2022/7/20	23:00:00	18.3611	90.7147	64.6900	2022/8/20	23:00:00	21.8480	82.0515	70.0108

2022/4/21	0:00:00	11.3891	71.1484	53.3731	2022/6/21	0:00:00	20.8897	82.3873	68.4777	2022/7/21	0:00:00	18.6336	86.5496	64.9827	2022/8/21	0:00:00	21.2147	83.0107	69.0478
2022/4/21	1:00:00	10.9913	66.2009	52.9398	2022/6/21	1:00:00	21.1445	80.5108	68.7674	2022/7/21	1:00:00	19.7077	80.3751	66.4512	2022/8/21	1:00:00	21.2792	83.5720	69.1910
2022/4/21	2:00:00	10.7914	61.7386	52.8082	2022/6/21	2:00:00	21.0671	79.5004	68.5767	2022/7/21	2:00:00	20.0984	77.0837	66.8945	2022/8/21	2:00:00	21.9689	80.6902	70.1056
2022/4/21	3:00:00	9.1271	61.4829	50.4564	2022/6/21	3:00:00	20.6854	81.8919	68.1150	2022/7/21	3:00:00	19.0422	81.1193	65.4165	2022/8/21	3:00:00	21.1900	81.8532	68.9301
2022/4/21	4:00:00	9.5883	54.9131	51.4266	2022/6/21	4:00:00	20.8172	80.6934	68.2528	2022/7/21	4:00:00	18.9900	80.1679	65.2896	2022/8/21	4:00:00	21.2808	80.5537	68.9894
2022/4/21	5:00:00	9.2158	64.2478	50.4391	2022/6/21	5:00:00	20.6414	87.1386	68.3654	2022/7/21	5:00:00	19.7759	89.6976	67.0529	2022/8/21	5:00:00	21.6630	79.3960	69.5210
2022/4/21	6:00:00	9.3325	60.2944	50.8079	2022/6/21	6:00:00	20.5747	86.5460	68.2180	2022/7/21	6:00:00	19.2491	93.0140	66.3161	2022/8/21	6:00:00	21.9523	78.9783	69.9516
2022/4/21	7:00:00	10.6301	64.3956	52.4787	2022/6/21	7:00:00	20.1812	89.1365	67.7092	2022/7/21	7:00:00	18.5331	91.3384	65.0090	2022/8/21	7:00:00	22.9656	80.9457	71.7307
2022/4/21	8:00:00	12.2153	71.5885	54.6146	2022/6/21	8:00:00	19.3303	93.0802	66.4598	2022/7/21	8:00:00	18.3955	91.8909	64.7947	2022/8/21	8:00:00	23.2984	73.2427	71.5917
2022/4/21	9:00:00	17.9870	52.0795	62.6959	2022/6/21	9:00:00	21.4977	85.5956	69.6900	2022/7/21	9:00:00	22.3766	81.4358	70.8200	2022/8/21	9:00:00	27.5973	58.5366	76.2761
2022/4/21	10:00:00	17.7564	59.1404	62.6218	2022/6/21	10:00:00	21.2241	90.0248	69.5338	2022/7/21	10:00:00	22.9609	81.7351	71.7897	2022/8/21	10:00:00	26.5802	62.7948	75.3744
2022/4/21	11:00:00	17.6736	63.5709	62.6479	2022/6/21	11:00:00	21.1606	92.2416	69.5733	2022/7/21	11:00:00	22.3207	85.3579	71.0355	2022/8/21	11:00:00	25.8260	65.9764	74.6531
2022/4/21	12:00:00	20.0614	61.0902	65.9468	2022/6/21	12:00:00	23.1754	85.3080	72.4458	2022/7/21	12:00:00	23.2571	83.0370	72.3828	2022/8/21	12:00:00	28.2612	60.8350	77.5130
2022/4/21	13:00:00	19.9490	62.7516	65.8783	2022/6/21	13:00:00	22.6120	89.0668	71.8176	2022/7/21	13:00:00	23.1533	84.0495	72.3008	2022/8/21	13:00:00	27.7817	63.2190	77.1505
2022/4/21	14:00:00	19.4711	65.5812	65.3352	2022/6/21	14:00:00	23.2291	91.6158	73.0833	2022/7/21	14:00:00	23.1474	81.9430	72.1096	2022/8/21	14:00:00	27.6221	64.9560	77.1479
2022/4/21	15:00:00	19.6028	67.0313	65.6015	2022/6/21	15:00:00	22.8647	85.0626	71.9112	2022/7/21	15:00:00	23.0012	79.9582	71.7044	2022/8/21	15:00:00	28.3951	63.6641	78.0927
2022/4/21	16:00:00	19.2034	70.2420	65.1641	2022/6/21	16:00:00	23.1388	80.4457	71.9668	2022/7/21	16:00:00	21.4149	81.4403	69.2661	2022/8/21	16:00:00	28.3101	63.2453	77.9129
2022/4/21	17:00:00	15.7158	83.6299	60.0825	2022/6/21	17:00:00	22.0261	84.7875	70.5051	2022/7/21	17:00:00	18.9943	87.9179	65.6455	2022/8/21	17:00:00	25.3938	67.5009	74.1860
2022/4/21	18:00:00	16.6420	76.9851	61.4549	2022/6/21	18:00:00	22.2615	83.1704	70.7683	2022/7/21	18:00:00	19.5319	87.3168	66.5186	2022/8/21	18:00:00	25.7395	70.7125	75.0561
2022/4/21	19:00:00	16.8614	75.5804	61.7661	2022/6/21	19:00:00	22.3427	81.4371	70.7654	2022/7/21	19:00:00	19.9458	88.1888	67.2592	2022/8/21	19:00:00	25.5535	74.2214	75.1611
2022/4/21	20:00:00	17.3183	74.6597	62.4519	2022/6/21	20:00:00	22.8636	77.0728	71.2435	2022/7/21	20:00:00	20.6295	83.6899	68.1345	2022/8/21	20:00:00	25.6513	73.1844	75.1972
2022/4/21	21:00:00	17.0414	77.0416	62.0843	2022/6/21	21:00:00	22.1755	81.4856	70.4989	2022/7/21	21:00:00	20.5882	83.0353	68.0268	2022/8/21	21:00:00	24.8353	76.8145	74.3184
2022/4/21	22:00:00	15.0547	85.2864	59.0095	2022/6/21	22:00:00	20.4355	87.4309	68.0384	2022/7/21	22:00:00	18.8540	87.1487	65.3762	2022/8/21	22:00:00	21.7980	83.7437	70.0529
2022/4/21	23:00:00	15.0310	87.3453	58.9823	2022/6/21	23:00:00	22.0067	79.0234	70.0417	2022/7/21	23:00:00	18.9959	86.3915	65.5795	2022/8/21	23:00:00	21.6340	82.4860	69.6947
2022/4/22	0:00:00	15.1020	85.8627	59.0915	2022/6/22	0:00:00	21.5009	82.3575	69.4691	2022/7/22	0:00:00	18.6659	86.4382	65.0318	2022/8/22	0:00:00	20.9798	83.5984	68.7024
2022/4/22	1:00:00	15.0547	85.0666	59.0082	2022/6/22	1:00:00	21.8014	82.1011	69.9389	2022/7/22	1:00:00	19.1959	84.6706	65.8316	2022/8/22	1:00:00	21.2147	81.5913	68.9526
2022/4/22	2:00:00	15.2675	81.6160	59.3317	2022/6/22	2:00:00	21.5391	84.4050	69.6750	2022/7/22	2:00:00	19.8883	77.8221	66.6036	2022/8/22	2:00:00	21.3539	80.6084	69.1106
2022/4/22	3:00:00	15.0014	80.4161	58.8946	2022/6/22	3:00:00	21.2273	87.5928	69.3760	2022/7/22	3:00:00	18.7315	81.4198	64.9281	2022/8/22	3:00:00	20.7110	82.4127	68.1887
2022/4/22	4:00:00	15.0874	78.1148	59.0181	2022/6/22	4:00:00	21.3101	86.5592	69.4446	2022/7/22	4:00:00	19.0330	81.6351	65.4252	2022/8/22	4:00:00	20.9820	80.4700	68.5035
2022/4/22	5:00:00	14.9805	78.5805	58.8512	2022/6/22	5:00:00	20.7166	81.9182	68.1672	2022/7/22	5:00:00	18.4235	81.0117	64.4142	2022/8/22	5:00:00	21.0814	86.6506	69.0694
2022/4/22	6:00:00	14.8208	77.5438	58.5938	2022/6/22	6:00:00	20.8838	84.1065	68.5777	2022/7/22	6:00:00	17.6983	85.8345	63.4006	2022/8/22	6:00:00	21.0384	85.8785	68.9472
2022/4/22	7:00:00	14.3295	83.5335	57.8118	2022/6/22	7:00:00	20.7258	87.8275	68.5494	2022/7/22	7:00:00	17.6897	92.6093	63.6040	2022/8/22	7:00:00	21.9792	83.1391	70.3048
2022/4/22	8:00:00	14.1091	79.3051	57.4651	2022/6/22	8:00:00	21.5745	84.6169	69.7483	2022/7/22	8:00:00	18.3745	90.5314	64.7058	2022/8/22	8:00:00	22.9785	76.1240	71.3441
2022/4/22	9:00:00	17.2075	65.5687	62.0317	2022/6/22	9:00:00	23.3990	81.5053	72.4786	2022/7/22	9:00:00	19.2889	88.8099	66.1833	2022/8/22	9:00:00	26.6399	63.3191	75.5232
2022/4/22	10:00:00	17.1038	67.5899	61.9335	2022/6/22	10:00:00	23.2259	81.1717	72.1697	2022/7/22	10:00:00	19.1174	92.2397	66.0524	2022/8/22	10:00:00	26.1228	64.6166	74.9301
2022/4/22	11:00:00	17.0000	68.7125	61.8085	2022/6/22	11:00:00	22.3454	86.6065	71.1741	2022/7/22	11:00:00	18.5831	93.0734	65.1658	2022/8/22	11:00:00	25.7862	65.9654	74.5937
2022/4/22	12:00:00	20.5882	60.9187	66.6817	2022/6/22	12:00:00	23.9048	81.5414	73.2999	2022/7/22	12:00:00	19.3910	91.5733	66.4912	2022/8/22	12:00:00	27.6532	59.7397	76.5111
2022/4/22	13:00:00	20.3350	64.2767	66.5197	2022/6/22	13:00:00	22.4997	84.8408	71.2905	2022/7/22	13:00:00	19.0728	93.9972	66.0560	2022/8/22	13:00:00	27.1108	60.4286	75.8374
2022/4/22	14:00:00	20.8349	66.2642	67.3685	2022/6/22	14:00:00	22.5674	84.9659	71.4123	2022/7/22	14:00:00	18.8556	96.8302	65.8017	2022/8/22	14:00:00	27.0721	61.0481	75.8603
2022/4/22	15:00:00	20.0436	65.6141	66.1725	2022/6/22	15:00:00	22.1105	88.0278	70.8903	2022/7/22	15:00:00	19.4754	94.2051	66.7671	2022/8/22	15:00:00	27.8924	58.5115	76.6828
2022/4/22	16:00:00	19.1782	67.6297	65.0037	2022/6/22	16:00:00	22.2196	88.1273	71.0814	2022/7/22	16:00:00	19.4163	92.2183	66.5663	2022/8/22	16:00:00	27.9113	58.9234	76.7639
2022/4/22	17:00:00	16.0577	73.6037	60.4823	2022/6/22	17:00:00	20.8172	97.4847	69.3122	2022/7/22	17:00:00	18.4337	96.3496	65.0365	2022/8/22	17:00:00	25.1438	67.0062	73.7641
2022/4/22	18:00:00	16.2147	72.6276	60.7067	2022/6/22	18:00:00	21.0214	95.3127	69.5334	2022/7/22	18:00:00	18.6186	94.2283	65.2749	2022/8/22	18:00:00	25.1863	69.3875	74.0799
2022/4/22	19:00:00	16.9087	72.7490	61.7708	2022/6/22	19:00:00	20.9623	94.3875	69.3699	2022/7/22	19:00:00	18.9266	94.2518	65.8128	2022/8/22	19:00:00	25.2637	71.9327	74.4684

2022/4/22	20:00:00	17.4441	68.8515	62.4743	2022/6/22	20:00:00	21.7595	93.4927	70.6958	2022/7/22	20:00:00	19.7877	91.5941	67.1733	2022/8/22	20:00:00	25.4675	70.3797	74.6090
2022/4/22	21:00:00	16.8242	68.5050	61.5416	2022/6/22	21:00:00	21.6117	95.4889	70.5809	2022/7/22	21:00:00	20.0038	89.9821	67.4555	2022/8/22	21:00:00	24.2461	73.9153	73.1118
2022/4/22	22:00:00	14.8918	74.6383	58.6929	2022/6/22	22:00:00	20.0995	97.5296	68.0408	2022/7/22	22:00:00	18.2401	94.5080	64.6259	2022/8/22	22:00:00	21.9571	79.3789	69.9891
2022/4/22	23:00:00	15.1966	72.1069	59.1461	2022/6/22	23:00:00	20.1710	95.5773	68.0571	2022/7/22	23:00:00	18.2665	93.6596	64.6398	2022/8/22	23:00:00	21.9206	78.6005	69.8732
2022/4/23	0:00:00	14.6477	74.4354	58.3145	2022/6/23	0:00:00	19.5921	95.0398	67.0130	2022/7/23	0:00:00	16.3168	99.2988	61.3572	2022/8/23	0:00:00	21.1072	81.6363	68.7817
2022/4/23	1:00:00	14.4053	77.7360	57.9382	2022/6/23	1:00:00	20.1350	91.2474	67.7499	2022/7/23	1:00:00	16.5458	99.4686	61.7714	2022/8/23	1:00:00	21.3738	81.4343	69.1993
2022/4/23	2:00:00	14.4112	77.1337	57.9477	2022/6/23	2:00:00	20.1635	90.5863	67.7613	2022/7/23	2:00:00	16.2190	99.2534	61.1811	2022/8/23	2:00:00	21.0449	82.4190	68.7319
2022/4/23	3:00:00	13.9215	75.6126	57.1850	2022/6/23	3:00:00	19.7060	88.7804	66.8865	2022/7/23	3:00:00	15.8137	96.3748	60.4154	2022/8/23	3:00:00	20.5460	83.2186	67.9691
2022/4/23	4:00:00	13.1802	76.9125	56.0134	2022/6/23	4:00:00	20.6000	83.0978	68.0499	2022/7/23	4:00:00	15.4943	93.4200	59.8214	2022/8/23	4:00:00	20.6970	82.8257	68.1916
2022/4/23	5:00:00	13.5350	75.4750	56.5838	2022/6/23	5:00:00	19.6630	92.4105	67.0014	2022/7/23	5:00:00	17.8198	87.2736	63.6504	2022/8/23	5:00:00	22.0087	80.2659	70.1379
2022/4/23	6:00:00	13.4554	74.0585	56.4738	2022/6/23	6:00:00	19.6620	90.2449	66.8876	2022/7/23	6:00:00	18.2165	84.5523	64.2128	2022/8/23	6:00:00	21.8302	81.0041	69.9054
2022/4/23	7:00:00	13.6108	77.2827	56.6869	2022/6/23	7:00:00	19.9668	93.8431	67.6036	2022/7/23	7:00:00	18.0375	85.2996	63.9446	2022/8/23	7:00:00	21.7055	85.1543	70.0028
2022/4/23	8:00:00	13.9586	76.5528	57.2382	2022/6/23	8:00:00	19.1975	96.6073	66.3959	2022/7/23	8:00:00	17.5241	89.5814	63.2258	2022/8/23	8:00:00	21.6050	78.9309	69.3954
2022/4/23	9:00:00	18.7358	61.9411	64.1075	2022/6/23	9:00:00	21.3767	85.5231	69.4846	2022/7/23	9:00:00	20.7376	82.4297	68.2330	2022/8/23	9:00:00	26.2469	64.7548	75.1263
2022/4/23	10:00:00	18.1289	66.4638	63.4087	2022/6/23	10:00:00	21.1918	85.1818	69.1554	2022/7/23	10:00:00	20.1560	85.6569	67.4697	2022/8/23	10:00:00	25.9674	64.4544	74.6864
2022/4/23	11:00:00	17.6112	69.6884	62.7499	2022/6/23	11:00:00	21.2967	84.3866	69.2748	2022/7/23	11:00:00	19.9474	86.9502	67.1944	2022/8/23	11:00:00	25.3831	65.6884	73.9738
2022/4/23	12:00:00	20.2151	64.2034	66.3422	2022/6/23	12:00:00	23.4329	74.5903	71.9181	2022/7/23	12:00:00	22.4508	79.7979	70.8101	2022/8/23	12:00:00	27.6677	59.3156	76.4759
2022/4/23	13:00:00	20.1710	66.2741	66.3958	2022/6/23	13:00:00	23.3458	75.5575	71.8684	2022/7/23	13:00:00	22.1755	81.6192	70.5091	2022/8/23	13:00:00	27.7242	59.5347	76.5836
2022/4/23	14:00:00	19.9205	67.6340	66.1023	2022/6/23	14:00:00	23.2243	75.0008	71.6308	2022/7/23	14:00:00	21.9686	82.6843	70.2536	2022/8/23	14:00:00	27.7989	58.9829	76.6152
2022/4/23	15:00:00	19.2491	70.4189	65.2414	2022/6/23	15:00:00	24.2322	71.4243	72.8490	2022/7/23	15:00:00	23.0840	77.3265	71.6119	2022/8/23	15:00:00	28.2978	57.9149	77.1641
2022/4/23	16:00:00	19.1040	72.4517	65.1164	2022/6/23	16:00:00	23.9688	72.8570	72.5845	2022/7/23	16:00:00	22.7109	80.0398	71.2462	2022/8/23	16:00:00	27.8742	59.3699	76.7716
2022/4/23	17:00:00	15.7158	81.3620	60.0539	2022/6/23	17:00:00	21.7347	77.0686	69.4675	2022/7/23	17:00:00	20.9623	87.4560	68.9227	2022/8/23	17:00:00	24.9229	71.1567	73.8691
2022/4/23	18:00:00	16.3270	83.0869	61.0734	2022/6/23	18:00:00	21.9697	77.5676	69.8742	2022/7/23	18:00:00	21.1633	83.6781	69.0083	2022/8/23	18:00:00	25.4089	71.6578	74.6595
2022/4/23	19:00:00	17.0887	83.1263	62.3180	2022/6/23	19:00:00	22.1282	77.4200	70.1132	2022/7/23	19:00:00	21.9729	83.9986	70.3586	2022/8/23	19:00:00	25.2589	75.0466	74.7944
2022/4/23	20:00:00	17.2263	82.4110	62.5230	2022/6/23	20:00:00	22.3631	79.0564	70.6118	2022/7/23	20:00:00	22.4228	81.4677	70.8973	2022/8/23	20:00:00	25.4288	72.8583	74.8203
2022/4/23	21:00:00	17.4086	82.5307	62.8228	2022/6/23	21:00:00	22.2734	76.8834	70.3004	2022/7/23	21:00:00	22.0718	83.2289	70.4628	2022/8/23	21:00:00	24.9890	72.3437	74.0931
2022/4/23	22:00:00	15.4868	87.0067	59.7422	2022/6/23	22:00:00	20.6011	80.7250	67.9071	2022/7/23	22:00:00	19.1250	93.5278	66.1250	2022/8/23	22:00:00	23.0091	78.2274	71.5703
2022/4/23	23:00:00	15.3922	87.8678	59.5922	2022/6/23	23:00:00	20.9902	77.0637	68.2961	2022/7/23	23:00:00	19.6512	89.2399	66.8175	2022/8/23	23:00:00	23.1134	77.0138	71.6314
2022/4/24	0:00:00	15.3815	87.5378	59.5710	2022/6/24	0:00:00	21.2757	73.7021	68.5177	2022/7/24	0:00:00	19.5657	89.1979	66.6706	2022/8/24	0:00:00	22.6952	78.0124	71.0553
2022/4/24	1:00:00	16.1749	81.2812	60.7941	2022/6/24	1:00:00	21.5616	70.3239	68.7200	2022/7/24	1:00:00	19.4534	90.7619	66.5580	2022/8/24	1:00:00	22.4672	78.2445	70.7131
2022/4/24	2:00:00	15.9911	85.1417	60.5564	2022/6/24	2:00:00	20.6785	72.9949	67.5546	2022/7/24	2:00:00	18.9519	92.3863	65.7736	2022/8/24	2:00:00	22.3356	79.1878	70.5781
2022/4/24	3:00:00	15.2514	88.6347	59.3617	2022/6/24	3:00:00	19.4265	77.2265	65.8445	2022/7/24	3:00:00	18.0966	95.5324	64.4124	2022/8/24	3:00:00	21.6287	81.7844	69.6360
2022/4/24	4:00:00	14.9025	88.9437	58.7744	2022/6/24	4:00:00	18.4267	82.1554	64.4645	2022/7/24	4:00:00	17.7580	94.3068	63.7776	2022/8/24	4:00:00	21.9813	81.5756	70.1916
2022/4/24	5:00:00	15.3089	82.9343	59.4100	2022/6/24	5:00:00	22.0159	69.2987	69.3273	2022/7/24	5:00:00	18.7965	87.1203	65.2788	2022/8/24	5:00:00	22.4855	79.9798	70.8802
2022/4/24	6:00:00	15.0294	83.2311	58.9558	2022/6/24	6:00:00	21.7256	72.3570	69.1135	2022/7/24	6:00:00	18.5643	87.4208	64.9027	2022/8/24	6:00:00	22.5200	80.5543	70.9813
2022/4/24	7:00:00	15.6879	86.6362	60.0737	2022/6/24	7:00:00	20.7419	79.0496	68.0293	2022/7/24	7:00:00	18.4192	89.8752	64.7561	2022/8/24	7:00:00	22.1979	81.3092	70.5216
2022/4/24	8:00:00	16.2899	85.7767	61.0620	2022/6/24	8:00:00	20.2538	83.8113	67.5258	2022/7/24	8:00:00	18.0343	92.0041	64.1775	2022/8/24	8:00:00	21.8958	79.8901	69.9290
2022/4/24	9:00:00	18.9116	76.4213	64.9980	2022/6/24	9:00:00	22.9313	78.9499	71.5078	2022/7/24	9:00:00	22.6400	76.2303	70.8234	2022/8/24	9:00:00	24.7514	67.1852	73.2041
2022/4/24	10:00:00	18.3375	82.0501	64.3156	2022/6/24	10:00:00	21.7939	85.9284	70.2051	2022/7/24	10:00:00	22.5868	77.1462	70.8139	2022/8/24	10:00:00	23.7918	68.4550	71.9062
2022/4/24	11:00:00	17.9972	90.3629	64.0560	2022/6/24	11:00:00	21.7788	86.7242	70.2379	2022/7/24	11:00:00	22.1239	81.2217	70.3954	2022/8/24	11:00:00	23.9897	69.2682	72.2773
2022/4/24	12:00:00	19.0304	94.4540	66.0029	2022/6/24	12:00:00	24.3101	77.2807	73.5393	2022/7/24	12:00:00	23.7172	76.5287	72.5363	2022/8/24	12:00:00	25.7921	63.1730	74.2886
2022/4/24	13:00:00	17.8198	93.6442	63.8632	2022/6/24	13:00:00	23.9107	77.4204	72.9232	2022/7/24	13:00:00	23.6624	76.6384	72.4604	2022/8/24	13:00:00	26.1604	61.6316	74.6384
2022/4/24	14:00:00	17.6854	93.5013	63.6252	2022/6/24	14:00:00	23.8473	78.6494	72.9377	2022/7/24	14:00:00	23.2039	78.1809	71.8748	2022/8/24	14:00:00	26.5227	60.4877	75.0162
2022/4/24	15:00:00	18.0671	88.5335	64.1095	2022/6/24	15:00:00	24.6343	74.3475	73.7539	2022/7/24	15:00:00	23.4538	78.2768	72.2793	2022/8/24	15:00:00	26.8743	58.9998	75.3284

2022/4/24	16:00:00	17.6983	88.2465	63.4783	2022/6/24	16:00:00	24.6090	74.9335	73.7738	2022/7/24	16:00:00	23.7038	77.8425	72.6357	2022/8/24	16:00:00	27.1420	59.2346	75.7311
2022/4/24	17:00:00	15.4245	91.3787	59.6804	2022/6/24	17:00:00	22.2465	87.8611	71.1061	2022/7/24	17:00:00	21.5407	86.6074	69.8323	2022/8/24	17:00:00	24.4896	71.3427	73.2314
2022/4/24	18:00:00	14.9450	94.0063	58.8713	2022/6/24	18:00:00	22.0852	84.7440	70.5994	2022/7/24	18:00:00	21.9148	84.4491	70.2966	2022/8/24	18:00:00	25.2169	71.5658	74.3581
2022/4/24	19:00:00	15.3299	94.2114	59.5430	2022/6/24	19:00:00	22.2524	84.5719	70.8618	2022/7/24	19:00:00	22.2497	85.8207	70.9538	2022/8/24	19:00:00	25.9276	66.5732	74.8696
2022/4/24	20:00:00	15.7884	91.3950	60.3046	2022/6/24	20:00:00	23.0679	78.8270	71.7146	2022/7/24	20:00:00	23.4329	80.9761	72.4863	2022/8/24	20:00:00	26.5593	64.3336	75.5290
2022/4/24	21:00:00	15.7411	90.2916	60.2093	2022/6/24	21:00:00	22.9184	81.1097	71.6684	2022/7/24	21:00:00	23.5038	79.8201	72.4970	2022/8/24	21:00:00	26.0061	68.2207	75.1735
2022/4/24	22:00:00	14.5488	91.1758	58.1788	2022/6/24	22:00:00	20.8618	86.2967	68.6806	2022/7/24	22:00:00	20.9118	89.5551	68.9724	2022/8/24	22:00:00	22.3646	81.1446	70.7778
2022/4/24	23:00:00	14.6999	91.1288	58.4373	2022/6/24	23:00:00	20.7817	85.8777	68.5210	2022/7/24	23:00:00	21.1295	86.6889	69.1521	2022/8/24	23:00:00	22.4931	80.4085	70.9264
2022/4/25	0:00:00	14.1360	92.6699	57.4671	2022/6/25	0:00:00	18.7874	95.0885	65.6061	2022/7/25	0:00:00	20.7344	85.5366	68.4212	2022/8/25	0:00:00	21.4324	84.9426	69.5367
2022/4/25	1:00:00	14.1269	90.6642	57.4577	2022/6/25	1:00:00	19.0922	92.0105	65.9983	2022/7/25	1:00:00	19.9619	87.1808	67.2312	2022/8/25	1:00:00	21.6432	82.2455	69.6924
2022/4/25	2:00:00	14.7337	86.5675	58.4822	2022/6/25	2:00:00	19.1572	89.6007	65.9978	2022/7/25	2:00:00	20.5258	84.0620	67.9869	2022/8/25	2:00:00	21.7055	80.6338	69.6778
2022/4/25	3:00:00	13.8693	89.0909	57.0269	2022/6/25	3:00:00	18.3127	90.9970	64.6181	2022/7/25	3:00:00	19.5700	85.7623	66.5036	2022/8/25	3:00:00	21.5335	80.1715	69.3687
2022/4/25	4:00:00	13.5350	89.8769	56.4541	2022/6/25	4:00:00	19.2550	87.4241	66.0601	2022/7/25	4:00:00	19.8007	85.0263	66.8472	2022/8/25	4:00:00	21.7862	79.6827	69.7384
2022/4/25	5:00:00	14.4660	85.4467	58.0357	2022/6/25	5:00:00	19.5389	87.7139	66.5503	2022/7/25	5:00:00	21.3853	76.7004	68.8926	2022/8/25	5:00:00	20.6524	85.9597	68.3114
2022/4/25	6:00:00	14.1892	84.2655	57.5803	2022/6/25	6:00:00	20.1103	83.6063	67.2790	2022/7/25	6:00:00	21.2789	75.7721	68.6627	2022/8/25	6:00:00	20.8115	85.9892	68.5776
2022/4/25	7:00:00	14.7558	82.8541	58.5075	2022/6/25	7:00:00	19.6722	90.4794	66.9172	2022/7/25	7:00:00	20.9284	79.2584	68.3397	2022/8/25	7:00:00	20.6959	88.0690	68.5143
2022/4/25	8:00:00	14.4273	80.9569	57.9724	2022/6/25	8:00:00	19.5700	90.4100	66.7394	2022/7/25	8:00:00	20.6650	80.0880	67.9708	2022/8/25	8:00:00	20.2955	89.5844	67.9285
2022/4/25	9:00:00	17.6806	66.0354	62.7369	2022/6/25	9:00:00	21.0424	84.5339	68.8661	2022/7/25	9:00:00	24.4510	69.7640	73.0165	2022/8/25	9:00:00	22.0426	82.2320	70.3401
2022/4/25	10:00:00	16.9291	68.8551	61.7062	2022/6/25	10:00:00	22.2922	80.3604	70.6001	2022/7/25	10:00:00	24.4983	71.7654	73.2866	2022/8/25	10:00:00	21.9001	84.2938	70.2609
2022/4/25	11:00:00	15.9099	70.6282	60.2117	2022/6/25	11:00:00	22.2245	81.7046	70.5949	2022/7/25	11:00:00	24.1446	74.5914	73.0202	2022/8/25	11:00:00	21.1975	85.5595	69.1901
2022/4/25	12:00:00	18.3289	59.6234	63.4392	2022/6/25	12:00:00	23.1947	78.3707	71.8768	2022/7/25	12:00:00	26.2797	68.6254	75.6274	2022/8/25	12:00:00	22.8419	81.6020	71.5860
2022/4/25	13:00:00	18.3762	58.7327	63.4708	2022/6/25	13:00:00	23.5038	77.8185	72.3174	2022/7/25	13:00:00	25.8787	72.1799	75.4324	2022/8/25	13:00:00	22.9430	78.6323	71.4996
2022/4/25	14:00:00	17.7886	61.5380	62.7461	2022/6/25	14:00:00	23.0335	79.7560	71.7389	2022/7/25	14:00:00	25.4379	73.0514	74.8553	2022/8/25	14:00:00	22.8973	79.3627	71.4882
2022/4/25	15:00:00	18.0595	62.6380	63.1700	2022/6/25	15:00:00	23.1904	79.6952	71.9847	2022/7/25	15:00:00	26.1803	70.8495	75.7376	2022/8/25	15:00:00	22.5479	80.2858	71.0047
2022/4/25	16:00:00	18.4976	60.8677	63.7255	2022/6/25	16:00:00	23.2022	78.7147	71.9185	2022/7/25	16:00:00	26.7264	70.3752	76.5054	2022/8/25	16:00:00	22.8898	78.4651	71.4011
2022/4/25	17:00:00	15.3847	72.0512	59.4323	2022/6/25	17:00:00	21.7923	87.9546	70.3498	2022/7/25	17:00:00	24.4451	80.6197	74.0823	2022/8/25	17:00:00	21.2964	87.3694	69.4767
2022/4/25	18:00:00	15.5546	76.5334	59.7403	2022/6/25	18:00:00	22.2674	84.6870	70.8955	2022/7/25	18:00:00	24.8622	77.0600	74.3860	2022/8/25	18:00:00	21.1626	89.3775	69.3861
2022/4/25	19:00:00	16.1701	74.0646	60.6631	2022/6/25	19:00:00	22.8410	83.7303	71.7614	2022/7/25	19:00:00	25.8814	73.5404	75.5906	2022/8/25	19:00:00	21.4319	88.5907	69.7882
2022/4/25	20:00:00	16.2324	75.1439	60.7784	2022/6/25	20:00:00	23.1754	80.2952	72.0125	2022/7/25	20:00:00	25.8577	73.4060	75.5390	2022/8/25	20:00:00	22.0592	82.3449	70.3757
2022/4/25	21:00:00	15.4438	80.3611	59.6046	2022/6/25	21:00:00	22.7453	83.0472	71.5484	2022/7/25	21:00:00	23.9392	79.3300	73.1477	2022/8/25	21:00:00	22.1582	81.4705	70.4697
2022/4/25	22:00:00	13.7527	81.7364	56.8800	2022/6/25	22:00:00	21.2864	88.5388	69.5392	2022/7/25	22:00:00	21.7095	86.0329	70.0725	2022/8/25	22:00:00	20.9045	86.5502	68.7680
2022/4/25	23:00:00	14.1150	78.5845	57.4769	2022/6/25	23:00:00	21.3810	87.0102	69.5938	2022/7/25	23:00:00	21.9315	83.8961	70.2830	2022/8/25	23:00:00	20.1675	88.0096	67.6222
2022/4/26	0:00:00	13.6490	79.1151	56.7326	2022/6/26	0:00:00	21.0763	90.0866	69.2864	2022/7/26	0:00:00	22.4524	78.8412	70.7368	2022/8/26	0:00:00	20.3503	87.3977	67.8937
2022/4/26	1:00:00	13.4463	79.2275	56.4086	2022/6/26	1:00:00	21.5203	87.3200	69.8482	2022/7/26	1:00:00	22.4239	76.6284	70.5167	2022/8/26	1:00:00	20.1052	87.5823	67.4934
2022/4/26	2:00:00	13.5280	78.5448	56.5451	2022/6/26	2:00:00	21.0510	91.2846	69.3218	2022/7/26	2:00:00	21.8766	77.7467	69.7406	2022/8/26	2:00:00	19.8197	87.7852	67.0254
2022/4/26	3:00:00	12.5276	82.0386	54.8906	2022/6/26	3:00:00	20.2479	94.8202	68.1486	2022/7/26	3:00:00	21.0687	79.2759	68.5646	2022/8/26	3:00:00	19.2235	88.3825	66.0527
2022/4/26	4:00:00	13.2367	78.9616	56.0775	2022/6/26	4:00:00	20.1898	96.3034	68.1315	2022/7/26	4:00:00	21.3676	77.9635	68.9513	2022/8/26	4:00:00	19.3880	86.9266	66.2586
2022/4/26	5:00:00	13.0620	75.3148	55.8494	2022/6/26	5:00:00	19.8496	93.7242	67.3934	2022/7/26	5:00:00	19.1943	82.9786	65.7493	2022/8/26	5:00:00	18.7220	94.3731	65.4613
2022/4/26	6:00:00	13.7231	72.3347	56.8992	2022/6/26	6:00:00	19.7238	94.9472	67.2387	2022/7/26	6:00:00	19.5109	80.4979	66.1415	2022/8/26	6:00:00	18.9929	93.2331	65.8825
2022/4/26	7:00:00	15.1213	76.3370	59.0598	2022/6/26	7:00:00	19.5243	98.3657	67.0616	2022/7/26	7:00:00	20.5951	79.1870	67.8039	2022/8/26	7:00:00	19.4687	90.7957	66.5858
2022/4/26	8:00:00	16.2281	66.9794	60.6275	2022/6/26	8:00:00	19.9264	96.8945	67.6991	2022/7/26	8:00:00	21.8734	78.0381	69.7570	2022/8/26	8:00:00	19.5224	91.0201	66.6889
2022/4/26	9:00:00	19.7786	56.6176	65.3106	2022/6/26	9:00:00	20.4387	95.9109	68.5470	2022/7/26	9:00:00	26.2765	66.4742	75.3705	2022/8/26	9:00:00	21.9555	82.4559	70.2153
2022/4/26	10:00:00	18.5105	66.0510	63.9524	2022/6/26	10:00:00	20.4102	98.6976	68.6615	2022/7/26	10:00:00	25.6819	70.7083	74.9688	2022/8/26	10:00:00	21.7716	87.1868	70.2595
2022/4/26	11:00:00	18.4159	68.2664	63.9010	2022/6/26	11:00:00	20.1457	100.0000	68.2623	2022/7/26	11:00:00	25.5959	73.1642	75.1100	2022/8/26	11:00:00	21.8663	87.8868	70.4692

2022/4/26	12:00:00	20.2834	64.7123	66.4703
2022/4/26	13:00:00	20.5984	63.0935	66.8286
2022/4/26	14:00:00	20.4269	63.3345	66.5969
2022/4/26	15:00:00	20.7198	60.8664	66.8645
2022/4/26	16:00:00	20.7876	60.7343	66.9519
2022/4/26	17:00:00	17.4441	65.1541	62.3645
2022/4/26	18:00:00	17.4650	70.3347	62.5499
2022/4/26	19:00:00	18.7492	58.9327	63.9984
2022/4/26	20:00:00	17.7757	64.9141	62.8392
2022/4/26	21:00:00	17.4838	69.8099	62.5625
2022/4/26	22:00:00	14.6198	76.3397	58.2745
2022/4/26	23:00:00	15.1154	73.6359	59.0326
2022/4/27	0:00:00	14.9203	75.7435	58.7422
2022/4/27	1:00:00	15.5486	72.5550	59.6875
2022/4/27	2:00:00	16.0061	69.3057	60.3364
2022/4/27	3:00:00	15.4540	72.6335	59.5437
2022/4/27	4:00:00	15.1509	75.0521	59.0971
2022/4/27	5:00:00	15.2396	82.0307	59.2898
2022/4/27	6:00:00	15.5132	81.1866	59.7247
2022/4/27	7:00:00	15.2143	85.2751	59.2735
2022/4/27	8:00:00	14.7396	84.4953	58.4861
2022/4/27	9:00:00	18.4337	70.9488	64.0333
2022/4/27	10:00:00	17.9354	74.7114	63.4097
2022/4/27	11:00:00	17.6365	79.1107	63.0856
2022/4/27	12:00:00	19.1959	75.2996	65.3908
2022/4/27	13:00:00	19.4308	76.0622	65.7938
2022/4/27	14:00:00	19.7152	75.8343	66.2263
2022/4/27	15:00:00	18.6987	77.7273	64.7195
2022/4/27	16:00:00	18.9621	75.2373	65.0243
2022/4/27	17:00:00	17.1409	77.8139	62.2613
2022/4/27	18:00:00	17.2844	78.2921	62.5016
2022/4/27	19:00:00	17.2801	79.4384	62.5269
2022/4/27	20:00:00	17.9945	77.4258	63.5967
2022/4/27	21:00:00	17.4542	80.2365	62.8288
2022/4/27	22:00:00	14.7396	92.6436	58.5099
2022/4/27	23:00:00	14.8789	94.7932	58.7596
2022/4/28	0:00:00	14.7590	98.4445	58.5613
2022/4/28	1:00:00	15.1063	99.1314	59.1856
2022/4/28	2:00:00	15.3342	98.0201	59.5841
2022/4/28	3:00:00	13.4108	100.0000	56.1395
2022/4/28	4:00:00	13.4554	99.6686	56.2230
2022/4/28	5:00:00	13.1609	94.9049	55.7543
2022/4/28	6:00:00	13.2453	90.9690	55.9487
2022/4/28	7:00:00	13.4286	91.1632	56.2603
2022/6/26	12:00:00	21.1531	97.0227	69.8779
2022/6/26	13:00:00	20.9462	99.4564	69.6681
2022/6/26	14:00:00	20.8145	99.8282	69.4552
2022/6/26	15:00:00	20.5747	99.9588	69.0320
2022/6/26	16:00:00	20.2775	99.9964	68.4992
2022/6/26	17:00:00	20.1990	98.8820	68.2945
2022/6/26	18:00:00	19.5760	99.3094	67.2016
2022/6/26	19:00:00	19.3539	99.6924	66.8221
2022/6/26	20:00:00	20.4312	93.8383	68.4109
2022/6/26	21:00:00	21.0660	91.7437	69.3776
2022/6/26	22:00:00	19.6308	92.3614	66.9432
2022/6/26	23:00:00	19.3185	92.8104	66.4263
2022/6/27	0:00:00	18.9857	95.0954	65.9538
2022/6/27	1:00:00	18.1617	95.9637	64.5425
2022/6/27	2:00:00	17.8122	95.5312	63.9131
2022/6/27	3:00:00	17.4161	91.7037	63.1049
2022/6/27	4:00:00	17.7591	87.8446	63.5674
2022/6/27	5:00:00	18.4751	91.4335	64.9133
2022/6/27	6:00:00	19.5184	88.0865	66.5347
2022/6/27	7:00:00	20.7553	89.5993	68.7098
2022/6/27	8:00:00	21.3483	82.6599	69.2417
2022/6/27	9:00:00	24.7810	70.4819	73.5852
2022/6/27	10:00:00	24.3402	74.1680	73.2817
2022/6/27	11:00:00	24.0086	75.4699	72.8928
2022/6/27	12:00:00	26.2781	63.1210	74.9801
2022/6/27	13:00:00	25.7040	65.2246	74.3908
2022/6/27	14:00:00	25.3358	63.4842	73.6671
2022/6/27	15:00:00	24.8165	61.1551	72.6810
2022/6/27	16:00:00	24.5902	59.8607	72.2307
2022/6/27	17:00:00	21.3531	71.6676	68.4977
2022/6/27	18:00:00	21.8396	72.4990	69.2978
2022/6/27	19:00:00	22.4051	72.5411	70.1651
2022/6/27	20:00:00	22.1191	73.0182	69.7643
2022/6/27	21:00:00	22.3013	70.2506	69.8284
2022/6/27	22:00:00	19.6797	79.9667	66.3852
2022/6/27	23:00:00	19.9576	79.2570	66.7915
2022/6/28	0:00:00	19.4249	80.4034	65.9986
2022/6/28	1:00:00	19.7120	78.4149	66.3559
2022/6/28	2:00:00	19.8791	78.4611	66.6236
2022/6/28	3:00:00	17.7757	86.7262	63.5585
2022/6/28	4:00:00	18.0536	81.3548	63.8303
2022/6/28	5:00:00	19.3808	79.1501	65.8665
2022/6/28	6:00:00	19.9608	80.0675	66.8410
2022/6/28	7:00:00	21.0849	80.9346	68.6994
2022/7/26	12:00:00	27.4618	67.9403	77.2996
2022/7/26	13:00:00	27.0161	70.6337	76.9741
2022/7/26	14:00:00	27.5225	70.5590	77.7287
2022/7/26	15:00:00	26.7044	70.7182	76.5138
2022/7/26	16:00:00	26.9839	70.2850	76.8822
2022/7/26	17:00:00	25.0208	74.6773	74.3860
2022/7/26	18:00:00	25.9287	71.8160	75.4673
2022/7/26	19:00:00	26.5888	69.4523	76.1871
2022/7/26	20:00:00	27.1833	71.0320	77.2767
2022/7/26	21:00:00	26.6581	71.9474	76.5926
2022/7/26	22:00:00	24.0752	74.3921	72.8938
2022/7/26	23:00:00	22.6018	78.6428	70.9585
2022/7/27	0:00:00	21.2773	82.6796	69.1275
2022/7/27	1:00:00	22.2674	74.7808	70.1282
2022/7/27	2:00:00	21.7052	75.1813	69.2853
2022/7/27	3:00:00	20.5392	76.1824	67.5335
2022/7/27	4:00:00	20.1517	77.6522	67.0103
2022/7/27	5:00:00	19.4991	85.4790	66.3717
2022/7/27	6:00:00	19.1056	89.8679	65.9226
2022/7/27	7:00:00	19.5077	87.8175	66.5032
2022/7/27	8:00:00	19.8985	87.9229	67.1652
2022/7/27	9:00:00	21.7729	80.8312	69.8005
2022/7/27	10:00:00	21.1015	86.1492	69.0699
2022/7/27	11:00:00	20.9564	93.6956	69.3151
2022/7/27	12:00:00	20.8365	88.5127	68.7788
2022/7/27	13:00:00	19.9221	90.7869	67.3602
2022/7/27	14:00:00	20.3425	87.4654	67.8846
2022/7/27	15:00:00	21.1692	83.3528	68.9963
2022/7/27	16:00:00	21.6697	82.3780	69.7450
2022/7/27	17:00:00	20.2076	89.2632	67.7610
2022/7/27	18:00:00	20.7198	87.1051	68.4946
2022/7/27	19:00:00	21.1886	85.5784	69.1766
2022/7/27	20:00:00	21.5498	83.7090	69.6437
2022/7/27	21:00:00	20.1205	86.8144	67.4759
2022/7/27	22:00:00	18.6793	90.3555	65.2184
2022/7/27	23:00:00	18.5536	86.8185	64.8602
2022/7/28	0:00:00	18.4708	84.6644	64.6361
2022/7/28	1:00:00	18.8110	80.9531	65.0365
2022/7/28	2:00:00	19.2282	77.2695	65.5342
2022/7/28	3:00:00	19.0905	74.5731	65.1934
2022/7/28	4:00:00	19.1825	73.4712	65.2841
2022/7/28	5:00:00	18.8852	80.0408	65.1159
2022/7/28	6:00:00	19.4486	78.5196	65.9433
2022/7/28	7:00:00	20.4651	78.9385	67.5817
2022/8/26	12:00:00	24.8299	81.3627	74.7776
2022/8/26	13:00:00	25.2589	81.0390	75.4360
2022/8/26	14:00:00	23.6166	81.7816	72.8556
2022/8/26	15:00:00	24.2214	78.9019	73.5563
2022/8/26	16:00:00	24.4423	79.2063	73.9380
2022/8/26	17:00:00	20.8605	86.7289	68.7059
2022/8/26	18:00:00	21.5786	86.0964	69.8596
2022/8/26	19:00:00	21.3636	89.3744	69.7267
2022/8/26	20:00:00	21.9426	85.9646	70.4548
2022/8/26	21:00:00	21.3690	86.8627	69.5636
2022/8/26	22:00:00	19.9568	91.5828	67.4629
2022/8/26	23:00:00	20.2331	89.5280	67.8195
2022/8/27	0:00:00	20.0348	90.5969	67.5422
2022/8/27	1:00:00	20.2353	88.0366	67.7376
2022/8/27	2:00:00	20.7696	85.3790	68.4698
2022/8/27	3:00:00	19.8009	87.5372	66.9807
2022/8/27	4:00:00	19.6767	88.8368	66.8399
2022/8/27	5:00:00	20.3444	83.8265	67.6752
2022/8/27	6:00:00	20.0143	88.3405	67.3828
2022/8/27	7:00:00	19.7068	89.4063	66.9204
2022/8/27	8:00:00	19.2993	90.3009	66.2726
2022/8/27	9:00:00	21.5819	84.3670	69.7427
2022/8/27	10:00:00	21.5502	86.1733	69.8176
2022/8/27	11:00:00	21.7695	86.3722	70.1968
2022/8/27	12:00:00	23.7316	81.5930	73.0245
2022/8/27	13:00:00	23.6854	83.5094	73.1251
2022/8/27	14:00:00	23.7558	83.0941	73.2021
2022/8/27	15:00:00	22.2194	84.8589	70.8296
2022/8/27	16:00:00	22.2194	82.9890	70.6856
2022/8/27	17:00:00	20.9105	87.0202	68.8079
2022/8/27	18:00:00	20.7556	89.3734	68.6962
2022/8/27	19:00:00	20.7728	91.9175	68.8847
2022/8/27	20:00:00	21.2841	88.9974	69.5663
2022/8/27	21:00:00	21.4066	90.0852	69.8486
2022/8/27	22:00:00	20.3148	91.7225	68.0856
2022/8/27	23:00:00	20.8099	87.6234	68.6779
2022/8/28	0:00:00	20.6218	86.0160	68.2640
2022/8/28	1:00:00	20.6126	87.9641	68.3678
2022/8/28	2:00:00	21.0126	82.9098	68.7114
2022/8/28	3:00:00	20.9196	85.5334	68.7279
2022/8/28	4:00:00	20.7040	90.0445	68.6503
2022/8/28	5:00:00	20.2928	89.7281	67.9323
2022/8/28	6:00:00	20.3616	89.8655	68.0572
2022/8/28	7:00:00	20.2165	92.8348	67.9802

2022/4/28	8:00:00	13.5769	90.8241	56.5173	2022/6/28	8:00:00	22.1164	75.7435	69.9672	2022/7/28	8:00:00	20.9951	71.4654	67.9406	2022/8/28	8:00:00	19.5654	91.9158	66.8079
2022/4/28	9:00:00	17.1909	76.6299	62.3081	2022/6/28	9:00:00	25.5030	56.0848	73.0975	2022/7/28	9:00:00	25.4422	58.0387	73.2273	2022/8/28	9:00:00	19.5488	92.4477	66.8061
2022/4/28	10:00:00	16.3802	78.9063	61.0802	2022/6/28	10:00:00	25.0267	57.4175	72.5869	2022/7/28	10:00:00	24.2333	65.6023	72.2864	2022/8/28	10:00:00	20.0568	87.8664	67.4280
2022/4/28	11:00:00	15.3508	83.8009	59.4861	2022/6/28	11:00:00	23.9935	61.6697	71.5648	2022/7/28	11:00:00	23.8398	69.1796	72.0449	2022/8/28	11:00:00	19.8391	89.2841	67.1380
2022/4/28	12:00:00	18.7519	77.3432	64.7872	2022/6/28	12:00:00	25.7115	53.6155	73.1068	2022/7/28	12:00:00	25.4793	61.6018	73.6679	2022/8/28	12:00:00	20.0116	87.8221	67.3497
2022/4/28	13:00:00	17.9961	79.3658	63.6675	2022/6/28	13:00:00	25.6271	51.7041	72.7821	2022/7/28	13:00:00	24.7853	66.0152	73.1344	2022/8/28	13:00:00	19.6772	88.7789	66.8377
2022/4/28	14:00:00	18.7680	76.1743	64.7626	2022/6/28	14:00:00	25.4879	52.9613	72.7355	2022/7/28	14:00:00	23.8043	70.0056	72.0684	2022/8/28	14:00:00	20.1256	89.7066	67.6472
2022/4/28	15:00:00	19.0051	71.6288	64.9282	2022/6/28	15:00:00	25.8653	52.4774	73.1843	2022/7/28	15:00:00	25.2530	66.2273	73.8415	2022/8/28	15:00:00	18.9160	93.8401	65.7762
2022/4/28	16:00:00	17.8967	71.0665	63.2251	2022/6/28	16:00:00	24.7203	57.9619	72.2199	2022/7/28	16:00:00	25.5293	64.2582	74.0304	2022/8/28	16:00:00	19.7498	91.0029	67.0772
2022/4/28	17:00:00	14.9584	80.5489	58.8262	2022/6/28	17:00:00	22.9609	63.5053	70.2527	2022/7/28	17:00:00	23.0749	74.3684	71.3447	2022/8/28	17:00:00	19.0257	97.2968	66.1237
2022/4/28	18:00:00	14.9498	74.2432	58.7808	2022/6/28	18:00:00	23.2259	62.3528	70.5337	2022/7/28	18:00:00	23.3028	74.0830	71.6721	2022/8/28	18:00:00	19.2993	98.0062	66.6430
2022/4/28	19:00:00	15.8610	75.7385	60.2095	2022/6/28	19:00:00	23.7790	60.2084	71.1250	2022/7/28	19:00:00	24.3462	68.8769	72.7722	2022/8/28	19:00:00	19.7530	95.8106	67.3353
2022/4/28	20:00:00	16.1910	72.5425	60.6691	2022/6/28	20:00:00	23.2275	63.7216	70.6550	2022/7/28	20:00:00	23.8398	75.9400	72.6737	2022/8/28	20:00:00	19.9299	94.8747	67.5955
2022/4/28	21:00:00	15.8701	81.5035	60.3051	2022/6/28	21:00:00	22.2744	66.3422	69.4849	2022/7/28	21:00:00	23.4893	75.9198	72.1245	2022/8/28	21:00:00	19.7719	95.0577	67.3287
2022/4/28	22:00:00	13.9774	84.9442	57.2290	2022/6/28	22:00:00	19.3142	71.4250	65.3879	2022/7/28	22:00:00	20.4194	83.4952	67.7786	2022/8/28	22:00:00	18.0253	99.9969	64.4454
2022/4/28	23:00:00	14.2129	83.2951	57.6214	2022/6/28	23:00:00	19.2948	68.9961	65.2419	2022/7/28	23:00:00	20.3264	81.2382	67.4950	2022/8/28	23:00:00	18.0027	99.0771	64.3724
2022/4/29	0:00:00	13.7688	84.7523	56.8859	2022/6/29	0:00:00	19.4813	66.5234	65.3971	2022/7/29	0:00:00	19.6781	82.0163	66.4888	2022/8/29	0:00:00	17.1620	99.8113	62.8864
2022/4/29	1:00:00	13.8693	84.1851	57.0549	2022/6/29	1:00:00	18.9191	67.7323	64.6249	2022/7/29	1:00:00	19.9119	78.2745	66.6655	2022/8/29	1:00:00	17.2754	99.3924	63.0787
2022/4/29	2:00:00	14.3015	80.5192	57.7703	2022/6/29	2:00:00	19.1276	65.6319	64.8363	2022/7/29	2:00:00	20.2151	73.3580	66.8652	2022/8/29	2:00:00	17.1824	99.5651	62.9165
2022/4/29	3:00:00	13.3001	82.8831	56.1341	2022/6/29	3:00:00	17.7827	69.7899	63.0105	2022/7/29	3:00:00	19.0051	75.4739	65.1018	2022/8/29	3:00:00	16.7657	99.9814	62.1779
2022/4/29	4:00:00	13.4700	81.9642	56.4199	2022/6/29	4:00:00	17.9853	67.6151	63.2384	2022/7/29	4:00:00	17.6322	80.8430	63.1334	2022/8/29	4:00:00	16.5964	100.0000	61.8736
2022/4/29	5:00:00	13.9140	82.1646	57.1388	2022/6/29	5:00:00	18.4089	70.9070	63.9942	2022/7/29	5:00:00	17.1199	80.9321	62.3108	2022/8/29	5:00:00	16.5701	99.9887	61.8259
2022/4/29	6:00:00	12.9464	89.0051	55.4665	2022/6/29	6:00:00	18.7342	72.6661	64.5607	2022/7/29	6:00:00	17.0995	80.4604	62.2655	2022/8/29	6:00:00	16.5163	100.0000	61.7294
2022/4/29	7:00:00	11.9449	89.0703	53.7713	2022/6/29	7:00:00	20.5333	67.3885	66.9942	2022/7/29	7:00:00	18.1111	86.3074	64.1030	2022/8/29	7:00:00	16.4792	99.9829	61.6623
2022/4/29	8:00:00	10.7010	93.2469	51.5121	2022/6/29	8:00:00	22.0761	61.0574	68.7947	2022/7/29	8:00:00	20.0157	72.4773	66.5102	2022/8/29	8:00:00	16.3099	100.0000	61.3578
2022/4/29	9:00:00	15.0149	80.3959	58.9160	2022/6/29	9:00:00	26.0238	47.1579	72.7853	2022/7/29	9:00:00	24.1150	63.6148	71.9236	2022/8/29	9:00:00	17.5802	99.8960	63.6411
2022/4/29	10:00:00	14.9364	82.6684	58.8011	2022/6/29	10:00:00	25.4363	53.1820	72.6906	2022/7/29	10:00:00	23.4506	71.1688	71.6404	2022/8/29	10:00:00	17.8888	99.6703	64.1885
2022/4/29	11:00:00	14.3859	84.8268	57.9035	2022/6/29	11:00:00	24.1091	61.2877	71.6924	2022/7/29	11:00:00	22.9243	75.9112	71.2415	2022/8/29	11:00:00	17.4221	100.0000	63.3599
2022/4/29	12:00:00	15.5486	82.1263	59.7922	2022/6/29	12:00:00	26.9941	50.0498	74.3835	2022/7/29	12:00:00	24.6004	70.5222	73.3169	2022/8/29	12:00:00	17.6103	99.2328	63.6745
2022/4/29	13:00:00	15.3654	81.3004	59.4871	2022/6/29	13:00:00	26.6038	52.0106	74.1101	2022/7/29	13:00:00	24.5913	71.5033	73.4017	2022/8/29	13:00:00	17.6012	99.9016	63.6790
2022/4/29	14:00:00	15.7207	80.0395	60.0450	2022/6/29	14:00:00	26.2942	54.4379	73.9846	2022/7/29	14:00:00	24.5810	72.1516	73.4512	2022/8/29	14:00:00	17.6968	99.1943	63.8284
2022/4/29	15:00:00	14.3961	86.2487	57.9196	2022/6/29	15:00:00	27.1140	52.8975	74.8972	2022/7/29	15:00:00	23.9021	75.5218	72.7319	2022/8/29	15:00:00	17.0910	99.2557	62.7443
2022/4/29	16:00:00	14.3714	85.3052	57.8792	2022/6/29	16:00:00	26.5635	56.0575	74.5422	2022/7/29	16:00:00	22.9727	76.5331	71.3696	2022/8/29	16:00:00	16.8781	99.5155	62.3689
2022/4/29	17:00:00	12.8770	89.1285	55.3474	2022/6/29	17:00:00	24.5902	55.8381	71.8266	2022/7/29	17:00:00	22.1266	80.1709	70.3198	2022/8/29	17:00:00	15.5460	99.9906	59.9827
2022/4/29	18:00:00	13.0216	88.5745	55.5999	2022/6/29	18:00:00	24.7558	53.7231	71.8363	2022/7/29	18:00:00	22.5395	77.2677	70.7492	2022/8/29	18:00:00	15.8621	99.9817	60.5515
2022/4/29	19:00:00	14.0000	87.6921	57.2541	2022/6/29	19:00:00	24.7128	55.1407	71.9227	2022/7/29	19:00:00	23.1963	77.0361	71.7637	2022/8/29	19:00:00	16.1819	100.0000	61.1275
2022/4/29	20:00:00	14.2618	89.3259	57.6905	2022/6/29	20:00:00	24.3150	61.0537	71.9612	2022/7/29	20:00:00	23.5603	74.7894	72.1333	2022/8/29	20:00:00	16.3642	99.8910	61.4535
2022/4/29	21:00:00	13.9865	92.0751	57.2117	2022/6/29	21:00:00	22.5540	69.3280	70.1347	2022/7/29	21:00:00	22.9668	76.1583	71.3287	2022/8/29	21:00:00	16.3249	99.5293	61.3761
2022/4/29	22:00:00	11.8337	98.1528	53.3484	2022/6/29	22:00:00	19.6604	68.4256	65.7582	2022/7/29	22:00:00	20.0807	84.8750	67.3013	2022/8/29	22:00:00	15.8884	98.5095	60.5779
2022/4/29	23:00:00	11.6659	97.4283	53.0694	2022/6/29	23:00:00	19.4475	66.4143	65.3420	2022/7/29	23:00:00	20.4995	82.5344	67.8521	2022/8/29	23:00:00	15.8809	97.5303	60.5505
2022/4/30	0:00:00	11.3875	96.6151	52.5999	2022/6/30	0:00:00	18.9400	66.9907	64.6229	2022/7/30	0:00:00	20.4371	82.5331	67.7505	2022/8/30	0:00:00	15.5702	97.0245	59.9932
2022/4/30	1:00:00	11.5133	100.0000	52.7239	2022/6/30	1:00:00	18.5820	66.8428	64.0895	2022/7/30	1:00:00	20.9166	78.2744	68.2578	2022/8/30	1:00:00	15.5686	96.5447	59.9850
2022/4/30	2:00:00	11.9831	99.9887	53.5699	2022/6/30	2:00:00	18.5949	67.5459	64.1373	2022/7/30	2:00:00	21.7729	73.0634	69.2369	2022/8/30	2:00:00	15.5084	96.7477	59.8808
2022/4/30	3:00:00	10.7956	99.9984	51.4322	2022/6/30	3:00:00	17.6720	70.0618	62.8529	2022/7/30	3:00:00	21.3708	72.6862	68.5945	2022/8/30	3:00:00	14.9982	97.8572	58.9851

2022/4/30	4:00:00	10.6715	99.9955	51.2089
2022/4/30	5:00:00	10.1210	99.9869	50.2184
2022/4/30	6:00:00	9.9834	99.9794	49.9711
2022/4/30	7:00:00	9.5174	99.9943	49.1316
2022/4/30	8:00:00	9.3298	99.9848	48.7943
2022/4/30	9:00:00	10.6580	99.9033	51.1881
2022/4/30	10:00:00	10.2527	92.8696	50.7508
2022/4/30	11:00:00	10.1641	90.7353	50.6879
2022/4/30	12:00:00	11.7095	82.9859	53.5378
2022/4/30	13:00:00	11.3714	82.7178	52.9942
2022/4/30	14:00:00	10.4893	82.1374	51.5801
2022/4/30	15:00:00	10.1506	83.1555	50.9871
2022/4/30	16:00:00	8.9669	84.6828	48.9711
2022/4/30	17:00:00	7.4860	88.3790	46.2753
2022/4/30	18:00:00	7.4623	87.5217	46.2947
2022/4/30	19:00:00	7.8870	86.3243	47.0844
2022/4/30	20:00:00	8.1284	85.4438	47.5412
2022/4/30	21:00:00	7.6354	90.3984	46.3910
2022/4/30	22:00:00	6.5039	94.7508	44.1197
2022/4/30	23:00:00	6.7614	95.6981	44.4977
		Month	Mean	**53.7156**
			SD	

2022/6/30	4:00:00	16.9087	70.5981	61.7183
2022/6/30	5:00:00	19.0422	66.0232	64.7294
2022/6/30	6:00:00	19.6410	64.3259	65.5185
2022/6/30	7:00:00	20.2904	69.5335	66.7594
2022/6/30	8:00:00	21.1386	63.1266	67.6058
2022/6/30	9:00:00	24.0886	46.1831	70.2213
2022/6/30	10:00:00	23.6801	50.1822	70.0692
2022/6/30	11:00:00	23.2555	54.3111	69.8744
2022/6/30	12:00:00	23.8430	53.3974	70.5812
2022/6/30	13:00:00	23.5882	55.6627	70.4452
2022/6/30	14:00:00	23.8016	52.7434	70.4652
2022/6/30	15:00:00	23.4936	54.2559	70.1904
2022/6/30	16:00:00	23.6565	54.6619	70.4469
2022/6/30	17:00:00	22.8206	56.7000	69.4864
2022/6/30	18:00:00	22.5335	63.7916	69.6607
2022/6/30	19:00:00	23.7704	47.0232	69.8956
2022/6/30	20:00:00	23.2469	47.7577	69.2917
2022/6/30	21:00:00	23.3103	45.1803	69.1469
2022/6/30	22:00:00	21.8057	47.3816	67.4156
2022/6/30	23:00:00	20.8172	52.9903	66.5051
		Month	Mean	**66.4331**
			SD	

2022/7/30	4:00:00	21.5143	71.1162	68.7042
2022/7/30	5:00:00	21.0542	77.0678	68.3970
2022/7/30	6:00:00	20.6295	78.2716	67.8027
2022/7/30	7:00:00	20.4742	82.7204	67.8221
2022/7/30	8:00:00	20.4091	80.4689	67.5831
2022/7/30	9:00:00	24.0650	71.4992	72.6025
2022/7/30	10:00:00	23.5560	76.7861	72.3067
2022/7/30	11:00:00	22.5868	82.8462	71.2734
2022/7/30	12:00:00	24.6848	75.7189	73.9711
2022/7/30	13:00:00	25.3239	73.2757	74.7047
2022/7/30	14:00:00	24.9493	70.6767	73.8591
2022/7/30	15:00:00	26.0206	65.7813	74.9155
2022/7/30	16:00:00	25.8964	65.8347	74.7401
2022/7/30	17:00:00	24.0355	68.3582	72.2594
2022/7/30	18:00:00	24.0650	67.2871	72.2013
2022/7/30	19:00:00	25.1213	61.4644	73.1451
2022/7/30	20:00:00	24.3902	66.3578	72.5899
2022/7/30	21:00:00	22.9211	74.1119	71.0855
2022/7/30	22:00:00	19.5760	84.8653	66.4678
2022/7/30	23:00:00	19.3927	84.7267	66.1586
2022/7/31	0:00:00	18.3745	88.8648	64.6409
2022/7/31	1:00:00	18.7148	84.7949	65.0438
2022/7/31	2:00:00	18.7519	83.2217	65.0379
2022/7/31	3:00:00	18.4976	80.6953	64.5211
2022/7/31	4:00:00	18.7992	78.5537	64.9139
2022/7/31	5:00:00	19.3201	81.6788	65.8918
2022/7/31	6:00:00	19.1690	81.6326	65.6451
2022/7/31	7:00:00	20.1291	83.7778	67.3194
2022/7/31	8:00:00	21.2687	73.3037	68.4801
2022/7/31	9:00:00	25.3266	62.4304	73.5404
2022/7/31	10:00:00	24.2247	70.7878	72.7760
2022/7/31	11:00:00	23.6640	75.4629	72.3556
2022/7/31	12:00:00	25.5857	73.9480	75.1808
2022/7/31	13:00:00	25.1224	73.3576	74.4039
2022/7/31	14:00:00	25.9496	70.4353	75.3419
2022/7/31	15:00:00	25.9470	68.7451	75.1454
2022/7/31	16:00:00	24.8461	66.3707	73.2599
2022/7/31	17:00:00	23.1549	75.0562	71.5279
2022/7/31	18:00:00	22.6416	69.8098	70.3049
2022/7/31	19:00:00	22.5986	73.9718	70.5763
2022/7/31	20:00:00	22.3234	75.1663	70.2450
2022/7/31	21:00:00	23.0453	69.3330	70.8703
2022/7/31	22:00:00	21.3203	72.9855	68.5376
2022/7/31	23:00:00	20.2006	78.6298	67.1433

2022/8/30	4:00:00	14.8364	98.5415	58.6999
2022/8/30	5:00:00	15.0224	99.0188	59.0347
2022/8/30	6:00:00	15.0036	99.4183	59.0033
2022/8/30	7:00:00	15.0122	99.2478	59.0177
2022/8/30	8:00:00	14.9310	99.1376	58.8717
2022/8/30	9:00:00	16.1379	96.1240	60.9832
2022/8/30	10:00:00	15.7293	96.0642	60.2627
2022/8/30	11:00:00	15.3648	96.5255	59.6250
2022/8/30	12:00:00	16.6292	93.2238	61.7860
2022/8/30	13:00:00	16.3749	94.5855	61.3714
2022/8/30	14:00:00	16.4867	93.7698	61.5502
2022/8/30	15:00:00	16.8491	92.9304	62.1601
2022/8/30	16:00:00	16.7856	93.4298	62.0619
2022/8/30	17:00:00	15.4885	96.2221	59.8402
2022/8/30	18:00:00	15.8277	95.5887	60.4294
2022/8/30	19:00:00	15.8814	94.4801	60.5081
2022/8/30	20:00:00	16.0820	93.2377	60.8379
2022/8/30	21:00:00	15.9761	94.4967	60.6735
2022/8/30	22:00:00	15.5847	94.4250	59.9895
2022/8/30	23:00:00	15.8874	93.0384	60.4978
2022/8/31	0:00:00	16.2862	89.2640	61.1195
2022/8/31	1:00:00	16.3749	89.3645	61.2716
2022/8/31	2:00:00	16.4405	88.7348	61.3703
2022/8/31	3:00:00	16.0567	88.7082	60.7218
2022/8/31	4:00:00	16.0744	88.3010	60.7452
2022/8/31	5:00:00	15.6137	92.8727	60.0222
2022/8/31	6:00:00	15.7100	93.3669	60.1948
2022/8/31	7:00:00	15.6906	92.0449	60.1449
2022/8/31	8:00:00	15.4261	91.9419	59.6887
2022/8/31	9:00:00	16.6534	83.7859	61.6215
2022/8/31	10:00:00	16.5142	84.1123	61.4000
2022/8/31	11:00:00	16.1524	86.7763	60.8507
2022/8/31	12:00:00	17.2813	82.6506	62.6190
2022/8/31	13:00:00	17.2985	81.8509	62.6245
2022/8/31	14:00:00	17.5651	81.5466	63.0471
2022/8/31	15:00:00	16.9700	82.9435	62.1196
2022/8/31	16:00:00	16.8292	84.7287	61.9320
2022/8/31	17:00:00	15.7971	89.5691	60.2950
2022/8/31	18:00:00	15.8282	89.8158	60.3513
2022/8/31	19:00:00	15.6250	93.5787	60.0500
2022/8/31	20:00:00	15.9013	90.3349	60.4830
2022/8/31	21:00:00	16.1314	88.4986	60.8445
2022/8/31	22:00:00	15.3256	90.7146	59.5051
2022/8/31	23:00:00	15.5342	88.7643	59.8403

Month	Mean<br>SD	**69.8107**	Month	Mean<br>SD	**69.8743**

Table S2: Sequencing data quality

Month(Group)	Sample	Raw reads	Clean reads	Q30(%)	GC content(%)	Mapped reads	Mapped rate(%)
Apr.(G1)	A1	55,677,474	54,825,748	90.8	52.07	51787845	94.46%
	A2	54,894,932	54,076,920	89.54	52.24	50257880	92.94%
	A3	54,771,118	53,992,268	90.57	51.55	49827689	92.29%
	A4	56,925,594	56,040,942	90.5	51.87	52351574	93.42%
	A5	49,602,752	48,855,874	91.91	51.9	46187842	94.54%
	A6	47,589,144	46,715,524	90.36	49.77	43417542	92.94%
Jun.(G2)	B1	64,377,504	62,239,172	93.82	51.44	60150133	96.64%
	B2	50,499,402	49,903,440	95.07	54.95	48424156	97.04%
	B3	87,896,688	85,297,886	94.47	49.42	80034924	93.83%
	B4	63,786,512	63,074,576	93.63	51.89	60700588	96.24%
	B5	91,709,868	89,976,950	95.02	52.22	86031294	95.61%
	B6	55,563,264	55,053,174	91.94	52.5	53129466	96.51%
Jul.(G3)	C1	49,738,336	49,311,764	94.28	55.37	48000567	97.34%
	C2	58,684,314	58,115,100	93.56	55.89	56363120	96.99%
	C3	57,024,762	56,156,590	92.25	55.47	53149056	94.64%
	C4	59,667,310	59,068,554	93.34	57.47	56335855	95.37%
	C5	26,235,178	25,883,186	92.74	55.64	23697926	91.56%
	C6	46,968,278	46,301,214	93.99	55.67	44649640	96.43%

Table S3: The DEGs and and their FDR values

gene_id	gene_name	trez	transcript_id	GO	KEGG	KO_ENTRY	EC	Description	trans_type	FPKM. A2	FPKM. A3	FPKM. A4
Mberchr1G0138	Mberchr1G0138		Mberchr1T0138	GO:0005524(ATP binding);GO:00057	NA	NA	NA	heat shock 70 kDa protein 13 isoform X2 [Odocoileus virginianus texanus]	mRNA	13.37	14.90	13.79
Mberchr1G0364	Mberchr1G0364		Mberchr1T0364	GO:0005737(cytoplasm)	NA	NA	NA	HSPB1-associated protein 1 isoform X1 [Odocoileus virginianus texanus]	mRNA	1.73	2.70	2.09
Mberchr2G0457	Mberchr2G0457		Mberchr2T0457	GO:0001530(lipopolysaccharide bindi	03018(RNA degradation);04940(	K04077	NA	60 kDa heat shock protein, mitochondrial isoform X1 [Bos taurus]	mRNA	103.18	126.64	123.42
Mberchr6G0595	Mberchr6G0595		Mberchr6T0595	GO:0001666(response to hypoxia);GO	04141(Protein processing in endo	K09487	NA	endoplasmin [Dasypus novemcinctus]	mRNA	87.41	123.13	128.56
Mberchr7G0599	Mberchr7G0599		Mberchr7T0599	GO:0005737(cytoplasm);GO:0006457(	NA	NA	NA	PREDICTED: hsp90 co-chaperone Cdc37-like 1 [Bison bison bison]	mRNA	7.36	7.45	7.79
Mberchr8G0877	Mberchr8G0877		Mberchr8T0877	GO:0005524(ATP binding);GO:00058	04530(Tight junction);04612(An	K09489	NA	PREDICTED: heat shock 70 kDa protein 4 [Capra hircus]	mRNA	161.35	188.70	198.59
Mberchr8G0931	Mberchr8G0931		Mberchr8T0931	GO:0005524(ATP binding);GO:00057	03018(RNA degradation);05152(	K04043	NA	stress-70 protein, mitochondrial [Ovis aries]	mRNA	153.74	164.02	169.74
Mberchr13G0281	Mberchr13G0281		Mberchr13T0281	GO:0005524(ATP binding);GO:00056	04141(Protein processing in endo	K09485	NA	heat shock protein 105 kDa isoform X3 [Bubalus	mRNA	26.48	42.80	31.10
Mberchr15G0618	Mberchr15G0618		Mberchr15T0618	GO:0005524(ATP binding);GO:00058	03040(Spliceosome);04141(Prote	K03283	NA	heat shock 70 kDa protein 14 isoform X1 [Bos taurus]	mRNA	4.03	4.32	4.56
Mberchr15G0708	Mberchr15G0708		Mberchr15T0708	GO:0000151(ubiquitin ligase complex	03040(Spliceosome);04141(Prote	K03283	NA	Heat shock cognate 71 kDa protein [Fukomys	mRNA	35.79	81.19	41.68
Mberchr16G0524	Mberchr16G0524		Mberchr16T0524	GO:0005515(protein binding);GO:000	04141(Protein processing in endo	K04079	NA	PREDICTED: heat shock protein HSP 90-alpha [Camelus bactrianus]	mRNA	252.69	482.62	328.57
Mberchr18G0244	Mberchr18G0244		Mberchr18T0244	GO:0000151(ubiquitin ligase complex	03040(Spliceosome);04141(Prote	K03283	NA	Heat shock cognate 71 kDa protein [Fukomys	mRNA	2505.39	4055.91	3363.70
Mberchr19G0169	Mberchr19G0169		Mberchr19T0169	GO:0005524(ATP binding);GO:00058	04141(Protein processing in endo	K09485	NA	PREDICTED: heat shock 70 kDa protein 4L isoform X1 [Ovis aries musimon]	mRNA	4.73	7.11	5.60
MberchrXG0214	MberchrXG0214		MberchrXT0214	GO:0005515(protein binding);GO:000	04141(Protein processing in endo	K04079	NA	PREDICTED: heat shock protein HSP 90-alpha isoform X1 [Pantholops	mRNA	0.50	0.90	0.30
MberchrXG0213	MberchrXG0213		MberchrXT0213	GO:0005634(nucleus);GO:0005737(cy	04141(Protein processing in endo	K04079	NA	PREDICTED: heat shock protein HSP 90-alpha isoform X2 [Nomascus	mRNA	5.26	8.77	6.55
Mberchr23G0102	Mberchr23G0102		Mberchr23T0102	GO:0005524(ATP binding);GO:00064	04141(Protein processing in endo	K04079	NA	Hsp90ab1 protein, partial [Mus musculus]	mRNA	0.29	0.18	0.17

Mberchr25G0227	Mberchr25G0227	Mberchr25T0227	GO:0005524(ATP binding);GO:00057	04141(Protein processing in endo	K04079	NA	heat shock protein HSP 90-beta [Bos taurus]	mRNA	303.27	375.09	326.01
Mberchr22G1060	Mberchr22G1060	Mberchr22T1060	GO:0004857(enzyme inhibitor activity	04141(Protein processing in endo	K09562	NA	hsp70-binding protein 1 [Bubalus bubalis]	mRNA	17.71	41.37	31.27

FPKM. A5	FPKM. A6	FPKM. A8	FPKM. C2	FPKM. C3	FPKM. C4	FPKM. C5	FPKM. C6	FPKM. C8	fc	log2(fc)	pval	qval	regulation	significant
11.96	13.99	25.49	0.44	0.32	1.28	0.67	0.48	0.87	23.00	4.52	0.00	0.00	up	yes
2.14	2.67	3.71	0.25	0.07	0.16	0.08	0.18	0.30	14.37	3.84	0.00	0.00	up	yes
117.51	132.31	137.76	7.46	1.05	11.57	3.88	10.74	12.15	15.81	3.98	0.00	0.00	up	yes
107.79	135.14	153.18	0.94	0.92	1.22	5.56	0.52	1.50	69.02	6.11	0.00	0.00	up	yes
7.47	9.72	14.72	0.55	0	3.21	0.77	3.02	1.96	5.74	2.52	0.02	0.04	up	yes
148.30	197.49	271.54	1.65	0.91	4.93	6.75	3.39	4.00	53.89	5.75	0.00	0.00	up	yes
151.88	177.12	176.57	3.34	0.18	8.27	4.30	11.59	10.25	26.18	4.71	0.00	0.00	up	yes
21.05	36.15	47.90	0.12	0	0.27	0.92	0.21	0.49	101.73	6.67	0.00	0.00	up	yes
3.90	4.26	7.17	0.67	0.17	1.06	0.42	1.01	1.31	6.10	2.61	0.00	0.00	up	yes
37.58	48.45	44.83	2.94	0.44	5.96	1.89	4.88	6.12	13.02	3.70	0.00	0.00	up	yes
256.82	317.00	303.73	3.16	1.55	6.68	9.44	7.14	5.74	57.60	5.85	0.00	0.00	up	yes
2719.51	3616.67	2933.96	240.15	21.73	475.34	163.33	501.52	518.00	10.00	3.32	0.00	0.00	up	yes
2.67	5.86	9.03	0.28	0	0.74	0	0	0.87	18.47	4.21	0.00	0.01	up	yes
0.17	0.13	0.66	0	0	0.03	0	0	0	103.60	6.69	0.00	0.00	up	yes
5.08	6.69	6.87	0.23	0	0.88	0.81	0	0	20.49	4.36	0.00	0.00	up	yes
0.27	0.16	0.17	0.02	0	0.05	0	0.05	0.03	8.09	3.02	0.00	0.01	up	yes

317.18	390.78	290.40	53.89	11.58	108.77	41.28	98.14	117.09	4.65	2.22	0.00	0.00	up	yes
56.47	71.47	15.52	25.48	112.59	51.35	208.73	241.96	47.41	0.34	-1.56	0.00	0.00	down	yes
